# Bioinformatics and systems biology approach to identify the pathogenetic link of Long COVID and Myalgic Encephalomyelitis/Chronic Fatigue Syndrome

**DOI:** 10.3389/fimmu.2022.952987

**Published:** 2022-09-16

**Authors:** Yongbiao Lv, Tian Zhang, Junxiang Cai, Chushuan Huang, Shaofeng Zhan, Jianbo Liu

**Affiliations:** ^1^The First Clinical Medical College, Guangzhou University of Chinese Medicine, Guangzhou, China; ^2^Guangdong Provincial Hospital of Chinese Medicine, Guangzhou, China; ^3^The First Affiliated Hospital of Guangzhou University of Chinese Medicine, Guangzhou, China

**Keywords:** Long COVID, myalgic encephalomyelitis/chronic fatigue syndrome, ME/CFS, systems biology, bioinformatics analyses, protein–protein interaction network

## Abstract

**Background:**

The COVID-19 pandemic, caused by severe acute respiratory syndrome coronavirus 2 (SARS-CoV-2), is a global crisis. Although many people recover from COVID-19 infection, they are likely to develop persistent symptoms similar to those of myalgic encephalomyelitis/chronic fatigue syndrome (ME/CFS) after discharge. Those constellations of symptoms persist for months after infection, called Long COVID, which may lead to considerable financial burden and healthcare challenges. However, the mechanisms underlying Long COVID and ME/CFS remain unclear.

**Methods:**

We collected the genes associated with Long COVID and ME/CFS in databases by restricted screening conditions and clinical sample datasets with limited filters. The common genes for Long COVID and ME/CFS were finally obtained by taking the intersection. We performed several advanced bioinformatics analyses based on common genes, including gene ontology and pathway enrichment analyses, protein–protein interaction (PPI) analysis, transcription factor (TF)–gene interaction network analysis, transcription factor–miRNA co-regulatory network analysis, and candidate drug analysis prediction.

**Results:**

We found nine common genes between Long COVID and ME/CFS and gained a piece of detailed information on their biological functions and signaling pathways through enrichment analysis. Five hub proteins (IL-6, IL-1B, CD8A, TP53, and CXCL8) were collected by the PPI network. The TF–gene and TF–miRNA coregulatory networks were demonstrated by NetworkAnalyst. In the end, 10 potential chemical compounds were predicted.

**Conclusion:**

This study revealed common gene interaction networks of Long COVID and ME/CFS and predicted potential therapeutic drugs for clinical practice. Our findings help to identify the potential biological mechanism between Long COVID and ME/CFS. However, more laboratory and multicenter evidence is required to explore greater mechanistic insight before clinical application in the future.

## Introduction

COVID-19 is a highly contagious viral pneumonia, which has hitherto caused an exceeding 6 million worldwide death toll among over 500 million confirmed cases (1). In addition to fever, dry cough, and shortness of breath, fatigue (38%) and myalgia (15%–44%) are also commonplace in COVID-19 (2). With the prevalence and transmission of the Omicron BA.2 variant virulent strain, influenza-like and cold-like symptoms such as cough, fever, and fatigue/myalgia become more prevalent (3). Even though many patients with COVID-19 have received treatment, approximately one in three patients still develop persisting symptoms within 12 weeks after onset (4). Regardless of the severity of severe acute respiratory syndrome coronavirus 2 (SARS-CoV-2) infection, Long COVID, typically manifested as fatigue or muscle weakness similar to the symptoms of myalgic encephalomyelitis/chronic fatigue syndrome (ME/CFS) (5, 6), can be experienced by all age groups (7, 8). Fatigue being the most common symptom of Long COVID could be a potential threat to public health and the economy due to restriction of daily working abilities and social participation (7, 9).

Similar to Long COVID with fatigue, ME/CFS, an intractable heterogeneous disease, has posed a non-negligible influence on millions of people globally, although a recent study shows the worrisome fact that up to 91% of patients remain undiagnosed or misdiagnosed owing to other clinical conditions (10–12). A prospective observational study presents that almost half of the patients with Long COVID at 6 months after SARS-CoV-2 infection fulfilled the diagnostic criteria of ME/CFS (13). If left ignored, cases of ME/CFS could double in the United States on account of the unprecedented COVID-19 pandemic (14).

People with acute COVID-19 and with ME/CFS have similar pathological mechanisms such as redox imbalance, systemic inflammation and neuroinflammation, impaired energy metabolism, and a hypometabolic state (15). Research shows that during the acute phase of COVID-19, the activation of immune-inflammatory pathways due to lung lesions and hypoxemia may lead to chronic fatigue syndrome-like symptoms such as fatigue and myalgia (16), while the pathophysiological mechanisms of Long COVID and ME/CFS remain ambiguous/elusive by far. Previous studies have indicated that autonomic dysfunction after a viral illness, tissue scarring, organ damage, immune system dysregulation, and autoantibodies could potentially be the underlying mechanisms that explain the development of Long COVID (17–20). ME/CFS gradually manifests as abnormalities of the central and autonomic nervous system including general downregulation of the hypothalamic–pituitary–adrenal axis, cognitive impairment manifested by slowed information processing speed and impaired memory and attention, abnormal signals in magnetic resonance imaging, a widespread state of neuroinflammation, and changes in systemic and cerebral hemodynamics that correlate with symptoms (21). Aside from the mechanisms mentioned above, infection, sleep disturbance, energy metabolism impairment, and impaired immune function are also involved in the progression of ME/CFS (21–25). Of particular concern is that compared with uninfected individuals, patients with COVID-19 exhibit increasing autoantibody reactivities (26), which may help to explain that patients suffering from Long COVID could manifest characteristics typically found in ME/CFS.

Bioinformatics analysis refers to the integration and analysis of biological data through a variety of bioinformatics tools, which is one of the important means of life science research. To exemplify, Hasan Mahmud et al. identified 10 hub genes related to SARS-CoV-2, idiopathic pulmonary fibrosis, and chronic obstructive pulmonary disease *via* bioinformatics and systems biology methods, which provides new perspectives for further in-depth research on comorbidity mechanisms of COVID-19, idiopathic pulmonary fibrosis (IPF), and chronic obstructive pulmonary disease (COPD); meanwhile, they predicted 10 possible drugs for clinical reference accordingly (27). A more accurate prediction could be performed by utilizing bioinformatics analysis for the purpose of understanding (physio)pathological molecular mechanisms of diseases and expediting the implementation of precision medicine (28).

**Figure 1:**
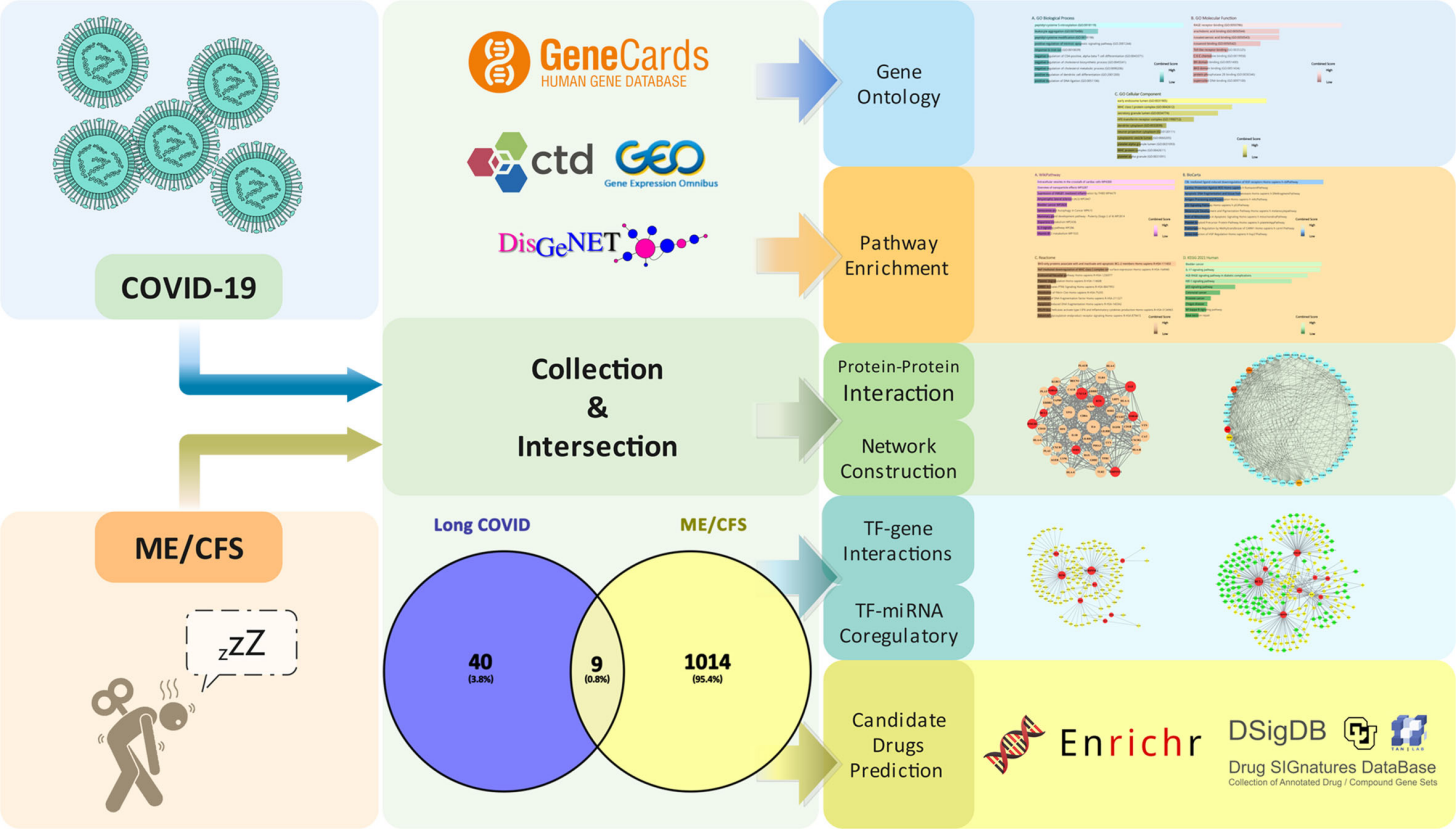
Workflow of our research.

Aiming to discover the mechanisms of ME/CFS associated with Long COVID, common genes of aforementioned diseases were located from acknowledged databases, the basis on which the common molecular pathogenesis and potential therapeutic drugs were predicted. The workflow of our research is presented in **Figure 1**.

## Materials and methods

### Collection of Long COVID and Myalgic Encephalomyelitis/Chronic Fatigue Syndrome-related genes

By searching CTD (http://ctdbase.org/) (29), GeneCards (https://www.genecards.org/) (30), and DisGeNET (https://www.disgenet.org/) (31) databases, we collected the genes related to COVID-19 and ME/CFS. In addition, we selected a relevant dataset, acquired from the Gene Expression Omnibus (GEO) database (https://www.ncbi.nlm.nih.gov/geo/ ) (32), for supplementary validation of disease genes. Dataset (GSE169687) (33) provides the total RNA sequenced from recovered COVID-19 patients’ blood several weeks post-infection. By performing differential gene analysis separately, we obtained the expression of differential genes in convalescent individuals at 12, 16, and 24 weeks post-infection compared with healthy controls and then merged and deduplicated the differentially expressed genes (DEGs). In the expression profiling datasets related to ME/CFS (including GSE130353, GSE128078, GSE14577, and GSE16059) (34–37), principal component analysis (PCA) shows that the heterogeneity between the disease group and the healthy control group was small after adjusting for batch effect. We found that the differential genetic results between the disease and normal control groups were disappointing, which may be attributed to the limitations of previous study designs on the one hand and the nature of the disease itself on the other.

According to the scoring rules of each database, we gathered the top 500 genes of each database. We analyzed the dataset by R programming language, DESeq2 (38), and limma (39) package. The adjusted *p*-value (false discovery rate (FDR)) <0.05 and fold change > 1.5-fold are used as cutoff criteria for DEGs. Subsequently, we obtained the related genes of Long COVID by taking the intersection of the two parts of the genes. We then overlapped the related genes of Long COVID and ME/CFS to obtain common genes for further analysis. We accessed to these websites on 27 April 2022.

### Gene ontology and pathway enrichment analyses

To understand a functional characteristic of the common genes in Long COVID and ME/CFS, a series of enrichment analyses were conducted utilizing Enrichr, a comprehensive gene set enrichment web tool (https://maayanlab.cloud/Enrichr/) (40), to gain detailed information on characterizing biological mechanisms and signaling pathways. Gene ontology (GO) (41, 42) includes three terms: biological process, molecular function, and cellular component. The Kyoto Encyclopedia of Genes and Genomes (KEGG) pathway (43) was used to recognize a metabolic pathway. For a more comprehensive understanding of the relevant signaling pathways, WikiPathways (44), Reactome (45), and BioCarta (46) databases were also used alongside the KEGG pathway.

### Protein–protein interaction analysis and network construction

In cellular as well as systems biology, understanding the interaction of an intracellular protein with another protein through the assessment and analysis of protein–protein interaction (PPI) networks can contribute to improved comprehension of protein function. Common genes were used to construct a PPI network by STRING (47) with a confidence score (0.4) as the minimum required interaction score, and all other parameters were set to their default. The PPI results were analyzed and visualized *via* Cytoscape (V3.8.2) (48–50). Through the cytoHubba (51), a plug-in of Cytoscape, the five hub proteins with the highest degree values were obtained by using the degree topological algorithm.

### Transcription factor-gene interactions

Transcription factors (TFs) are proteins that can bind to specific DNA sequences and regulate the expression of genes. NetworkAnalyst 3.0 (https://www.networkanalyst.ca/) (52) was used to analyze the interaction of the common genes and transcription factors and to assess the impact of the TF on the expression and functional pathways of the common genes. Transcription factor and gene target data were derived from the ENCODE ChIP-seq data (53–55). Only peak intensity signal <500 and the predicted regulatory potential score <1 are used (using the BETA Minus algorithm). TF–gene regulatory network was constructed and visualized by Cytoscape.

### Transcription factor–miRNA coregulatory network

MicroRNAs (miRNAs), which mediate target mRNA degradation or translation inhibition, are one class of endogenous short non-coding RNAs (56). We must understand the deregulation of gene expression in different physiological and disease conditions by comprehending transcriptional networks of regulation between TFs and miRNAs (56–60). The common genes were submitted to NetworkAnalyst 3.0 to generate a TF–miRNA coregulatory network. The literature-curated regulatory interaction information was collected from RegNetwork (http://www.regnetworkweb.org/) (61). Relevant results were also visualized by Cytoscape.

### Prediction of candidate drugs

Evaluating protein–drug interactions is important for understanding the structural features recommended for receptor sensitivity. The common genes were uploaded to the Drug Signatures Database (DSigDB, http://dsigdb.tanlab.org/DSigDBv1.0/) (62), which consists of 22,527 gene sets for further candidate drug prediction. Access to DSigDB is acquired through the Enrichr platform. The candidate drugs were sorted by adjusted *p*-value from small to large, and the adjusted *p*-value <0.01 was considered statistically significant.

## Result

### Collection of Long COVID and Myalgic Encephalomyelitis/Chronic Fatigue Syndrome-related genes

By searching CTD, GeneCards, and DisGeNET databases, we collected the genes related to COVID-19 and ME/CFS. To improve the credibility of the data, we gathered the top 500 genes of each database according to the scoring rules of each database. If the raw data are less than 500, we included all retrieved data. On this basis, we obtained 500, 500, and 118 ME/CFS-related genes from CTD, GeneCards, and DisGeNET, respectively. After that, we gained 1,023 ME/CFS-related genes by merging and deduplicating the results collected from three databases.

Analogously, we gained 1,233 COVID-19-related genes by CTD, GeneCards, and DisGeNET. At the same time, we collected 1,186 Long COVID-related genes using the cutoff criteria (FDR < 0.05 and fold change > 1.5-fold). By taking the intersection of the results of the database and the dataset, we included 49 Long COVID-related genes. Details on genes collected from above can be found in the **Supplementary Material**.

**Figure 2:**
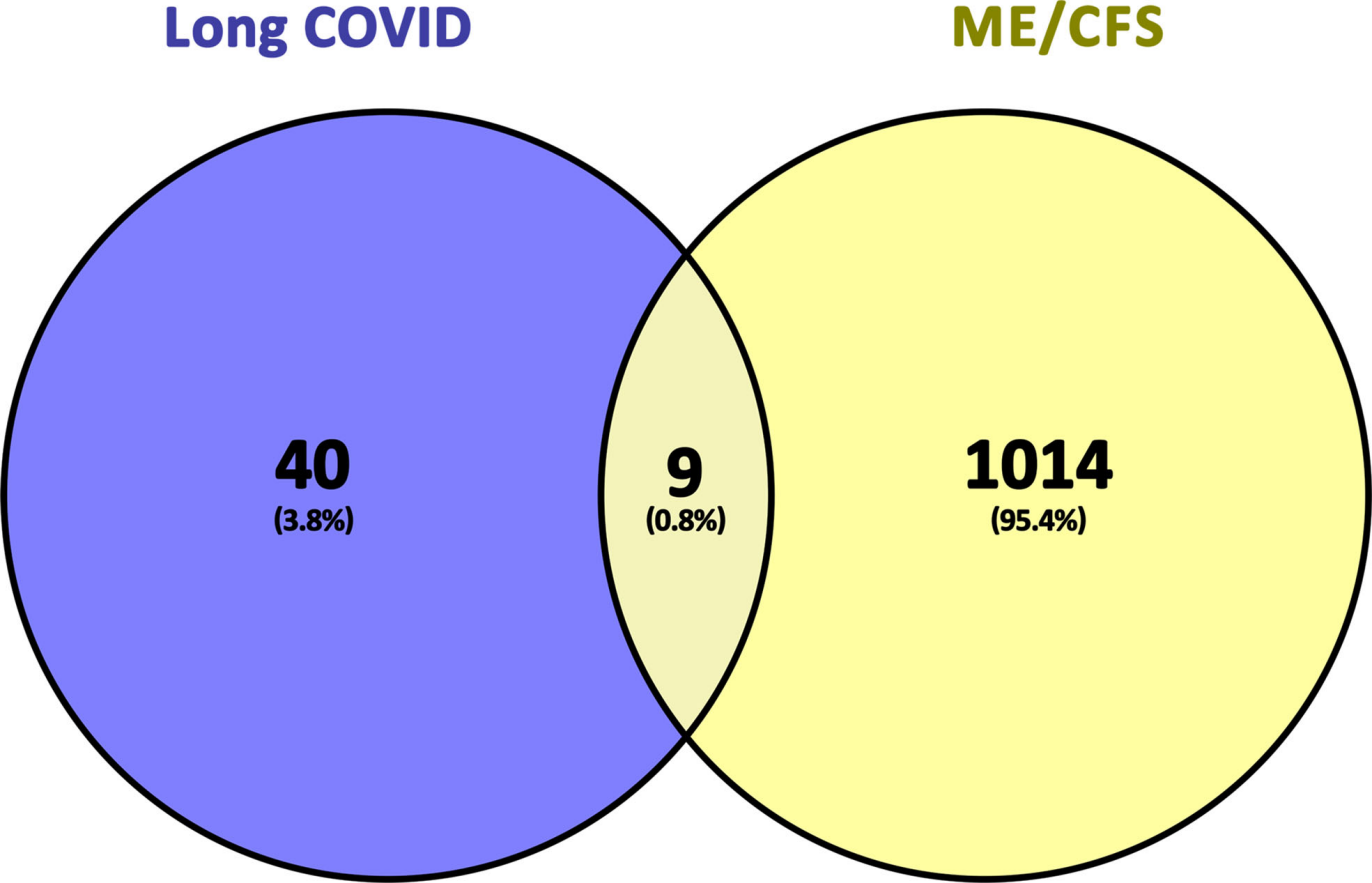
Common genes representation through a Venn diagram. 9 genes were found as common genes from 49 related genes of long COVID and 1023 related genes of ME/CFS.

Finally, we obtained nine common genes of ME/CFS and Long COVID by cross-processing the related genes of the two diseases. The screening process and results are shown in **Table 1** and **Figure 2**.

**Table 1:** Collection of Long COVID and ME/CFS-related genes.

Disease	Database or GEO	Data sources	Amount of raw data	Filter condition	Amount of data after filtering and deduplication	Merge		Common genes
ME/CFS	Database	CTD	532	If the raw data are greater than 500, then 500 are taken; if the raw data are less than 500, then all are included	500	1,023	1,023	9
		GeneCards	6,468		500			
		DisGeNET	118		118			
Long COVID	Database	CTD	9,879		500	1,233	49	
		GeneCards	4,600		500			
		DisGeNET	1,843		500			
	GEO	GSE169687	19,452	FDR < 0.05 and fold change > 1.5-fold	1,186	1,186		

ME/CFS, myalgic encephalomyelitis/chronic fatigue syndrome; GEO, Gene Expression Omnibus.

### Gene ontology and pathway enrichment analyses

**Figure 3:**
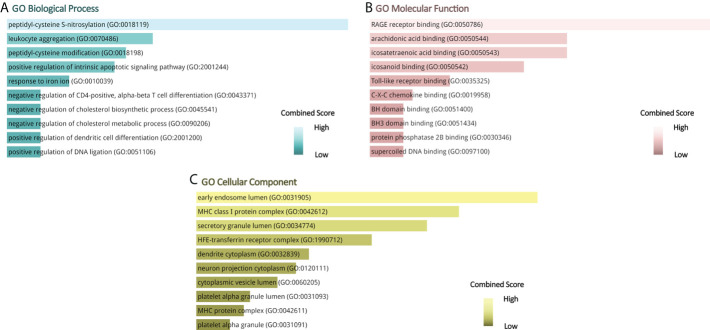
GO terms of common genes between long COVID and ME/CFS. **(A)** Biological Processes, **(B)** Molecular Function, **(C)** Cellular Component.

**Figure 4:**
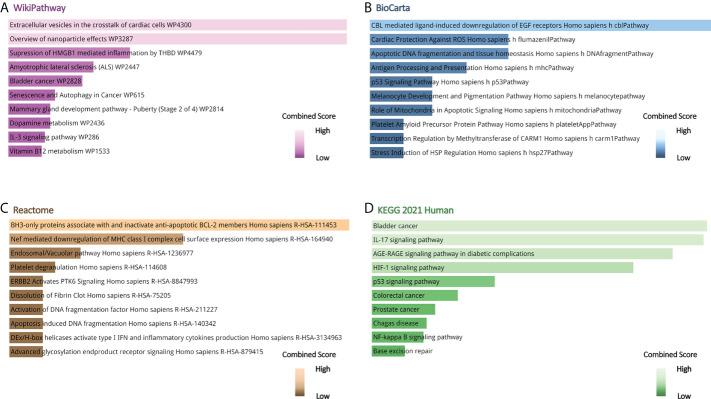
Pathway enrichment analysis of common genes between long COVID and ME/CFS. **(A)** Wikipathway, **(B)** BioCarta Pathway, **(C)** Reactome Pathway, **(D)** KEGG Human Pathway.

Gene ontology, pathway enrichment analysis, and the visualization of results were executed by Enrichr. A combined score was performed by the Enrichr web tool, which is determined by the log of the *p*-value and *Z*-score. The gene ontology analysis of common genes (CXCL8, B2M, SOD1, BCL2, EGF, SERPINE1, S100A8, S100A9, and HMGB1), using the GO database as an annotation source, was acquired within three categories (biological process, cellular component, and molecular function). The most impacted pathways of the common genes among ME/CFS and Long COVID were gathered from four global databases, including KEGG, WikiPathways, Reactome, and BioCarta. The top 10 GO terms and pathways are summarized in **Tables 2**, **3**, respectively, and presented in the form of bar graphs in **Figures 3**, **4**.

**Table 2:** Ontological analysis of common genes among Long COVID and ME/CFS.

Category	GO ID	Term	*p*-Values	Genes
GO Biological process	GO:0018119	Peptidyl-cysteine *S*-nitrosylation	1.80E−06	S100A9, S100A8
	GO:0070486	Leukocyte aggregation	5.03E−06	S100A9, S100A8
	GO:0018198	Peptidyl-cysteine modification	6.47E−06	S100A9, S100A8
	GO:2001244	Positive regulation of intrinsic apoptotic signaling pathway	1.72E−09	BCL2, S100A9, S100A8, SOD1
	GO:0010039	Response to iron ion	1.40E−05	BCL2, B2M
	GO:0043371	Negative regulation of CD4-positive, alpha-beta T-cell differentiation	0.002248161	HMGB1
	GO:0045541	Negative regulation of cholesterol biosynthetic process	0.002248161	SOD1
	GO:0090206	Negative regulation of cholesterol metabolic process	0.002248161	SOD1
	GO:2001200	Positive regulation of dendritic cell differentiation	0.002248161	HMGB1
	GO:0051106	Positive regulation of DNA ligation	0.002248161	HMGB1
GO Molecular function	GO:0050786	RAGE receptor binding	5.28E−09	HMGB1, S100A9, S100A8
	GO:0050544	Arachidonic acid binding	2.70E−06	S100A9, S100A8
	GO:0050543	Icosatetraenoic acid binding	2.70E−06	S100A9, S100A8
	GO:0050542	Icosanoid binding	3.78E−06	S100A9, S100A8
	GO:0035325	Toll-like receptor binding	9.88E−06	S100A9, S100A8
	GO:0019958	C-X-C chemokine binding	0.002248161	HMGB1
	GO:0051400	BH domain binding	0.002697256	BCL2
	GO:0051434	BH3 domain binding	0.002697256	BCL2
	GO:0030346	Protein phosphatase 2B binding	0.002697256	SOD1
	GO:0097100	Supercoiled DNA binding	0.002697256	HMGB1
GO Cellular component	GO:0031905	Early endosome lumen	0.002248161	B2M
	GO:0042612	MHC class I protein complex	0.002697256	B2M
	GO:0034774	Secretory granule lumen	1.20E−09	EGF, SERPINE1, HMGB1, B2M, S100A9, S100A8
	GO:1990712	HFE-transferrin receptor complex	0.003594909	B2M
	GO:0032839	Dendrite cytoplasm	0.004940043	SOD1
	GO:0120111	Neuron projection cytoplasm	0.005388063	SOD1
	GO:0060205	Cytoplasmic vesicle lumen	1.52E−05	HMGB1, S100A9, S100A8
	GO:0031093	Platelet alpha granule lumen	3.92E−04	EGF, SERPINE1
	GO:0042611	MHC protein complex	0.008965773	B2M
	GO:0031091	Platelet alpha granule	7.06E−04	EGF, SERPINE1

ME/CFS, myalgic encephalomyelitis/chronic fatigue syndrome.

**Table 3:** Pathway enrichment analysis of common genes among Long COVID and ME/CFS.

Category	Pathways	*p*-Values	Genes
WikiPathways Human	Extracellular vesicles in the crosstalk of cardiac cells WP4300	3.07E−05	EGF, SOD1
	Overview of nanoparticle effects WP3287	3.07E−05	CXCL8, BCL2
	Suppression of HMGB1 mediated inflammation by THBD WP4479	0.004043466	HMGB1
	Amyotrophic lateral sclerosis (ALS) WP2447	1.25E−04	BCL2, SOD1
	Bladder cancer WP2828	1.39E−04	CXCL8, EGF
	Senescence and autophagy in cancer WP615	1.15E−05	CXCL8, SERPINE1, BCL2
	Mammary gland development pathway—puberty (stage 2 of 4) WP2814	0.005835903	EGF
	Dopamine metabolism WP2436	0.005835903	SOD1
	IL-3 signaling pathway WP286	2.09E−04	CXCL8, BCL2
	Vitamin B12 metabolism WP1533	2.18E−04	SERPINE1, SOD1
BioCarta	CBL-mediated ligand-induced downregulation of EGF receptors *Homo sapiens* h cbl Pathway	0.003594909	EGF
	Cardiac protection against ROS *Homo sapiens* h flumazenil pathway	0.004940043	SOD1
	Apoptotic DNA fragmentation and tissue homeostasis *Homo sapiens* h DNA fragment pathway	0.004940043	HMGB1
	Antigen processing and presentation *Homo sapiens* h MHC pathway	0.005388063	B2M
	p53 signaling pathway *Homo sapiens* h p53 pathway	0.005835903	BCL2
	Melanocyte development and pigmentation pathway *Homo sapiens* h melanocyte pathway	0.005835903	BCL2
	Role of mitochondria in apoptotic signaling *Homo sapiens* h mitochondria pathway	0.005835903	BCL2
	Platelet amyloid precursor protein pathway *Homo sapiens* h platelet App pathway	0.006283564	SERPINE1
	Transcription regulation by methyltransferase of CARM1 *Homo sapiens* h carm1 pathway	0.006283564	BCL2
	Stress induction of HSP regulation *Homo sapiens* h hsp27 pathway	0.006283564	BCL2
Reactome	BH3-only proteins associate with and inactivate anti-apoptotic BCL-2 members *Homo sapiens* R-HSA-111453	0.003594909	BCL2
	Nef mediated downregulation of MHC class I complex cell surface expression *Homo sapiens* R-HSA-164940	0.004491844	B2M
	Endosomal/vacuolar pathway *Homo sapiens* R-HSA-1236977	0.005388063	B2M
	Platelet degranulation *Homo sapiens* R-HSA-114608	1.15E−05	EGF, SERPINE1, SOD1
	ERBB2 activates PTK6 signaling *Homo sapiens* R-HSA-8847993	0.005835903	EGF
	Dissolution of fibrin clot *Homo sapiens* R-HSA-75205	0.005835903	SERPINE1
	Activation of DNA fragmentation factor *Homo sapiens* R-HSA-211227	0.005835903	HMGB1
	Apoptosis-induced DNA fragmentation *Homo sapiens* R-HSA-140342	0.005835903	HMGB1
	DEx/H-box helicases activate type I IFN and inflammatory cytokines production *Homo sapiens* R-HSA-3134963	0.005835903	HMGB1
	Advanced glycosylation endproduct receptor signaling *Homo sapiens* R-HSA-879415	0.005835903	HMGB1
KEGG 2019 Human	Bladder cancer	1.46E−04	CXCL8, EGF
	IL-17 signaling pathway	8.27E−06	CXCL8, S100A9, S100A8
	AGE-RAGE signaling pathway in diabetic complications	9.97E−06	CXCL8, SERPINE1, BCL2
	HIF-1 signaling pathway	1.29E−05	EGF, SERPINE1, BCL2
	p53 signaling pathway	4.65E−04	SERPINE1, BCL2
	Colorectal cancer	6.45E−04	EGF, BCL2
	Prostate cancer	8.20E−04	EGF, BCL2
	Chagas disease	9.06E−04	CXCL8, SERPINE1
	NF-kappa B signaling pathway	9.41E−04	CXCL8, BCL2
	Base excision repair	0.01475517	HMGB1

ME/CFS, myalgic encephalomyelitis/chronic fatigue syndrome; GEO, Gene Expression Omnibus.

### Protein–protein interaction analysis and network construction

**Figure 5:**
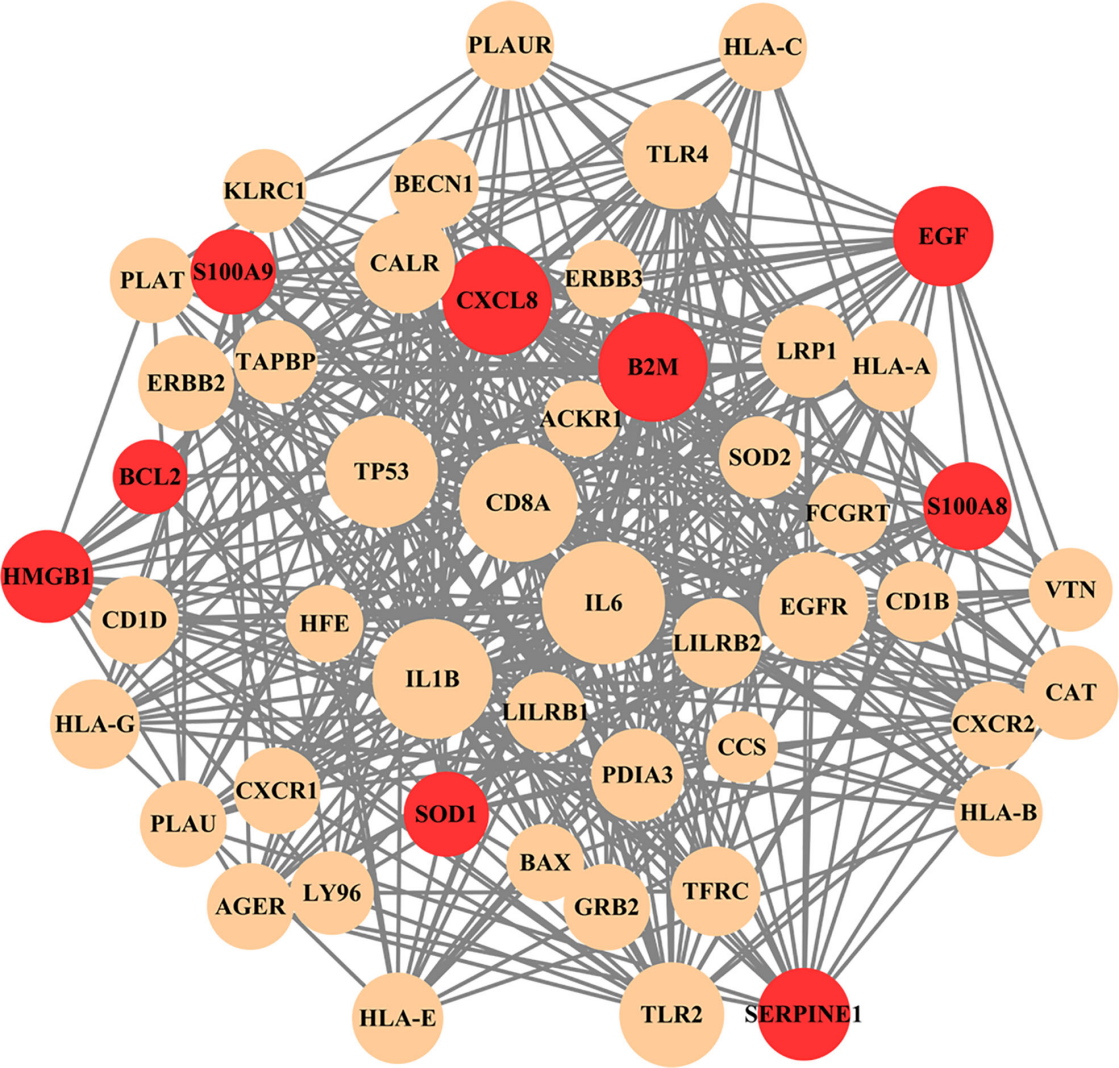
PPI network of common genes among long COVID and ME/CFS. In the figure, nodes in red color represent common genes and edges represent the interactions between nodes. The analyzed network holds 50 nodes and 375 edges.

**Figure 6:**
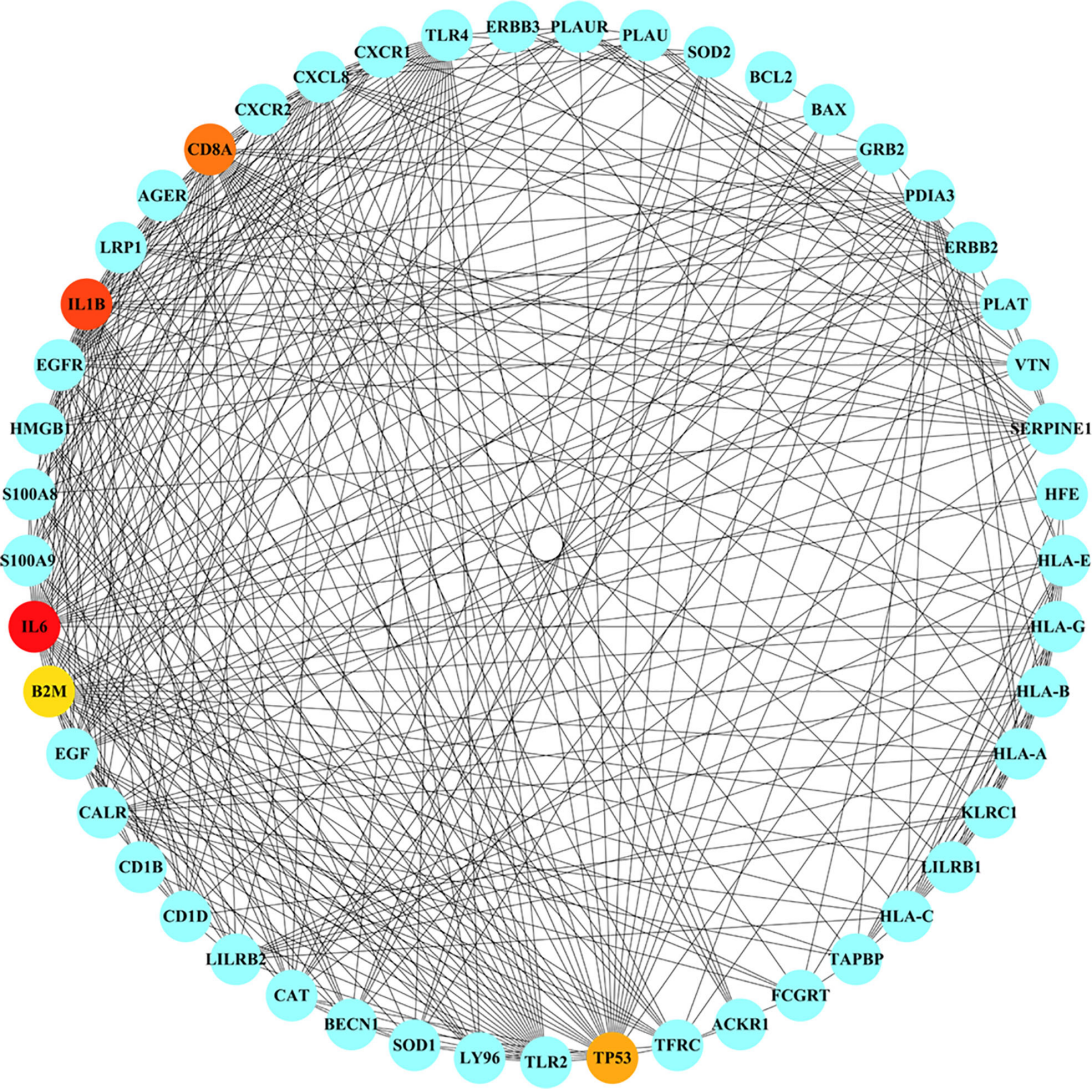
Detection of hub genes from the PPIs network of common genes. The highlighted 5 hub genes, based on their degree, are IL6, IL1B, CD8A, TP53, CXCL8. The network has 49 nodes and 373 edges.

The nine common genes were provided as input in STRING, and the file generated from the analysis is reintroduced into Cytoscape for visual representation. As shown in **Figure 5**, the PPI network of common genes consists of 50 nodes and 375 edges. According to the degree in the PPI network, the top 5 hub proteins as IL-6, IL-1B, CD8A, TP53, and CXCL8 were listed through the cytoHubba plugin. The interaction of hub proteins with other proteins in the PPI network is demonstrated in **Figure 6**.

### Transcription factor–gene interactions

**Figure 7:**
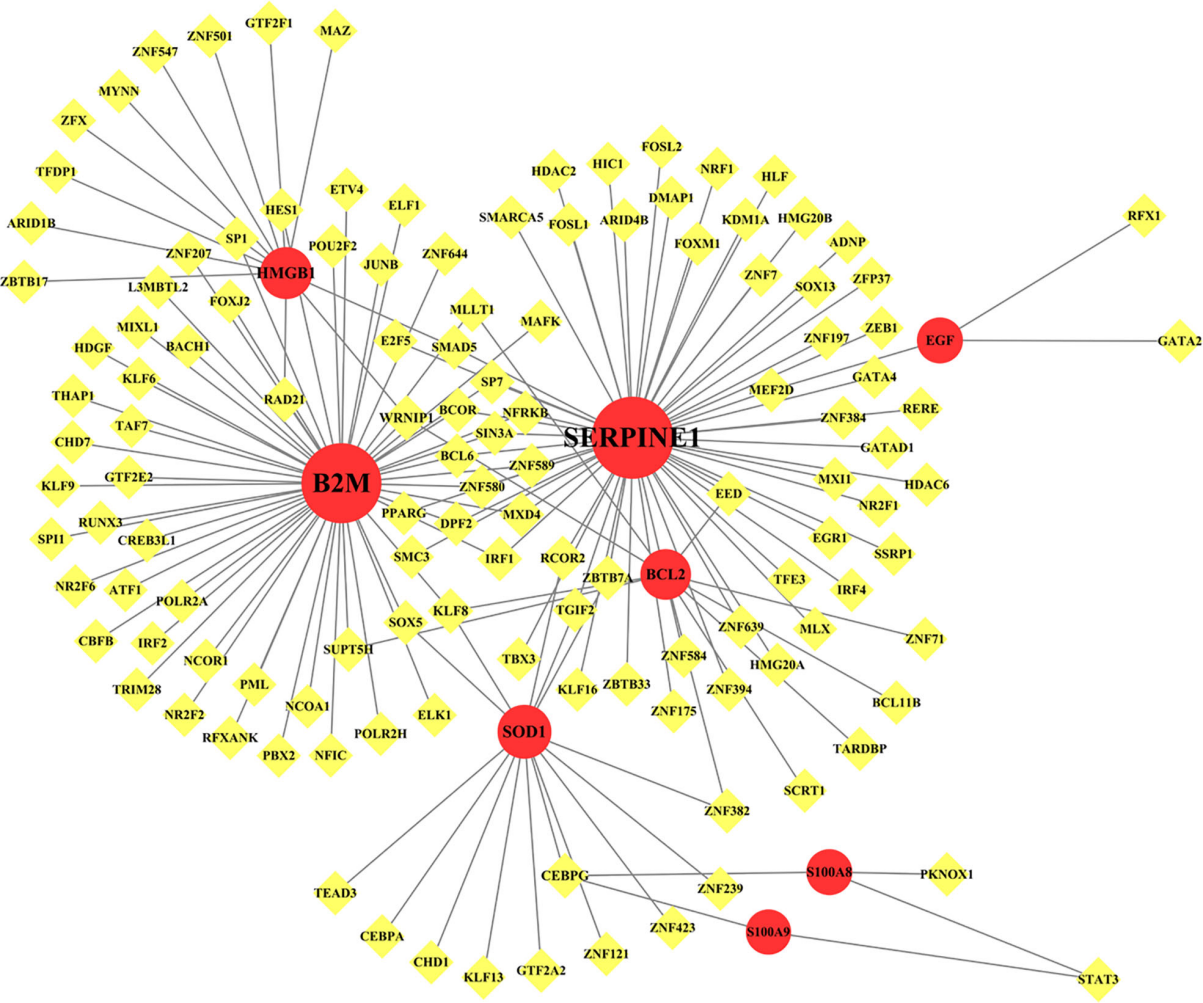
Network for TF-gene interaction with common differentially expressed genes. The highlighted red color node represents the common genes and other nodes represent TF-genes. The network consists of 136 nodes and 156 edges.

TF–gene interactions showed the interaction of nine common genes and TF genes by using NetworkAnalyst. The TF–gene interaction network consists of 136 nodes and 156 edges (**Figure 7**). Among them, SERPINE1 is regulated by 57 TF genes, and B2M is regulated by 54 genes (**Table 4**). The degree values of transcription factors CEBPG, KLF8, and WRNIP1 in the TF–gene interaction network were all 3.

**Table 4:** TF–gene interaction.

Identified genes	Transcription factors
B2M	POLR2H, CHD7, NFIC, SP7, PBX2, BACH1, ELF, WRNIP1, KLF6, NR2F2, CREB3L1, ZNF589, THAP1 SIN3A, BCOR, HES1, ZNF644, IRF2, SP1, MIXL1, CBFB, ZNF580, MAFK, MXD4, FOXJ2, JUNB, NCOR1, SPI1, IRF1, GTF2E2, DPF2, BCL6, POU2F2, ELK1, NFRKB, POLR2A, NR2F6, HDGF ETV4, RAD21, ATF1, KLF8, KLF9, SUPT5H, SOX5, PML, TRIM28, L3MBTL2, RFXANK, RUNX3, MLLT1, ZNF207, NCOA1, TAF7
BCL2	WRNIP1, KLF8, SUPT5H, MLLT1, BCL11B, ZNF382, EED, TARDBP, ZNF71, SCRT1
EGF	MEF2D, RFX1, GATA2
HMGB1	WRNIP1, RAD21, ZNF547, TFDP1, ZFX, ZBTB17, MYNN, SMAD5, ARID1B, GTF2F1, MAZ, ZNF501
S100A8	PKNOX1, CEBPG, STAT3
S100A9	CEBPG, STAT3
SERPINE1	SP7, ZNF589, SIN3A, BCOR, ZNF580, MXD4, IRF1, DPF2, BCL6, NFRKB, EED, MEF2D, SMAD5, ZNF197, TBX3, NR2F1, E2F5, ZBTB7A, GATA4, FOSL2, TGIF2, HIC1, ZEB1, IRF4, FOXM1, HMG20A, MXI1, SMARCA5, ZFP37, PPARG, ZNF584, EGR1, MLX, ARID4B, TFE3, ZNF394, RERE, HLF, ZBTB33, HMG20B, ZNF175, NRF1, ZNF7, HDAC2, FOSL1, SMC3, ADNP, HDAC6, ZNF639, SOX13, KLF16, RCOR2, KDM1A, GATAD1, SSRP1, DMAP1, ZNF384
SOD1	KLF8, SOX5, ZNF382, CEBPG, ZBTB7A, TGIF2, RCOR2 KLF13, ZNF121, GTF2A2, ZNF239, ZNF423 TEAD3, CHD1, CEBPA

### Transcription factor–miRNA coregulatory network

**Figure 8:**
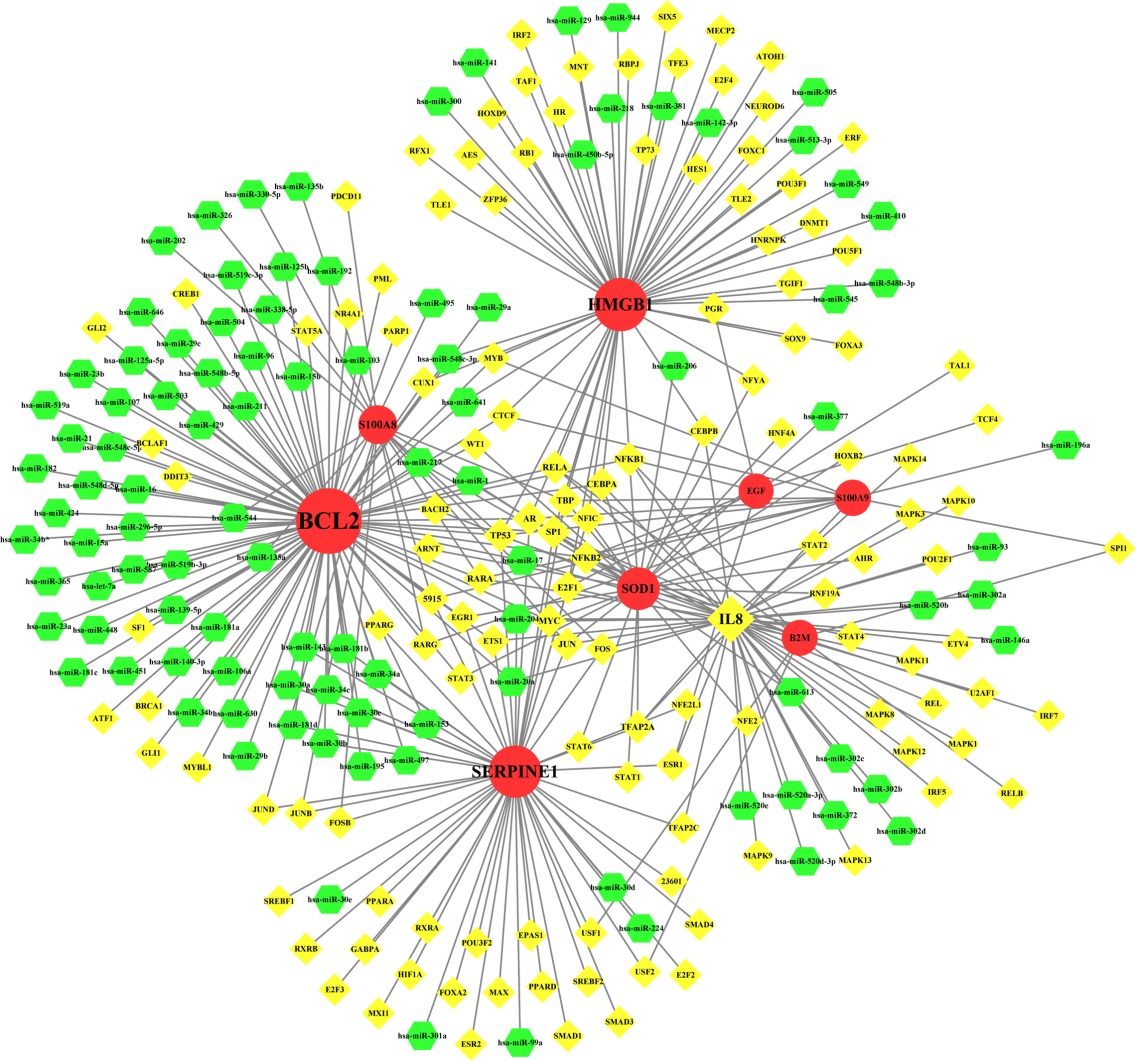
The network presents the TF-miRNA coregulatory network. The network consists of 240 nodes and 327 edges including 130 TF-genes, 102 miRNA and 9 common genes. The nodes in red color are the common genes, yellow nodes represent miRNA and green nodes indicate TF-genes.

TF–miRNA coregulatory network is also generated using NetworkAnalyst 3.0. The TF–miRNA coregulatory network comprises 240 nodes and 327 edges (**Figure 8**). A total of 130 TF genes and 102 miRNAs have interacted with the nine common genes (**Table 5**).

**Table 5:** TF–miRNA coregulatory interaction.

Hub genes	Transcription factors or miRNA
B2M	USF1, RELA, NFKB2, NFKB1, USF2, SPI1, SP1, RELB, IRF7, E2F1
BCL2	RELA, NFKB2, NFKB1, MYC, WT1, TP53, STAT5A, STAT3, SP1, 5915, RARG, RARA, PPARG, MYBL1, MYB, GLI2, GLI1, ETS1, EGR1, CUX1, CTCF, CREB1, CEBPA, BRCA1, ATF1, AR, BCLAF1, DDIT3, NR4A1, PARP1, PML, SF1, hsa-let-7a, hsa-miR-1, hsa-miR-103, hsa-miR-106a, hsa-miR-107, hsa-miR-125a-5p, hsa-miR-125b, hsa-miR-139-5p, hsa-miR-140-3p, hsa-miR-143, hsa-miR-153, hsa-miR-15a, hsa-miR-15b, hsa-miR-16, hsa-miR-17, hsa-miR-181a, hsa-miR-181b, hsa-miR-181c, hsa-miR-181d, hsa-miR-182, hsa-miR-192, hsa-miR-195, hsa-miR-204, hsa-miR-20a, hsa-miR-21, hsa-miR-211, hsa-miR-217, hsa-miR-23a, hsa-miR-23b, hsa-miR-296-5p, hsa-miR-29a, hsa-miR-29b, hsa-miR-29c, hsa-miR-30a, hsa-miR-30b, hsa-miR-30c, hsa-miR-338-5p, hsa-miR-34a, hsa-miR-34b, hsa-miR-34b*, hsa-miR-34c, hsa-miR-365, hsa-miR-424, hsa-miR-429, hsa-miR-448, hsa-miR-451, hsa-miR-495, hsa-miR-497, hsa-miR-503, hsa-miR-504, hsa-miR-519a, hsa-miR-519b-3p, hsa-miR-519c-3p, hsa-miR-548b-5p, hsa-miR-548c-3p, hsa-miR-548c-5p, hsa-miR-548d-5p, hsa-miR-587, hsa-miR-630, hsa-miR-641, hsa-miR-646, hsa-miR-96
EGF	NFKB2, NFKB1, TCF4, TAL1, PGR, ESR1, E2F1
HMGB1	RELA, NFKB1, TP53, TBP, SP1, SOX9, POU3F1, NFYA, NFIC, MYB, E2F4, E2F1, CUX1, CTCF, CEBPB, CEBPA, AR, AES, ATOH1, DNMT1, ERF, FOXA3, FOXC1, HES1, HNRNPK, HOXD9, HR, IRF2, MECP2, MNT, NEUROD6, POU5F1, RB1, RBPJ, RFX1 SIX5, TAF1, TFE3, TGIF1, TLE1, TLE2, TP73, ZFP36, hsa-miR-129, hsa-miR-141, hsa-miR-142-3p, hsa-miR-218, hsa-miR-300, hsa-miR-381, hsa-miR-410, hsa-miR-450b-5p, hsa-miR-505, hsa-miR-513-3p, hsa-miR-545, hsa-miR-548b-3p, hsa-miR-548c-3p, hsa-miR-549, hsa-miR-641, hsa-miR-944
S100A8	JUN, TP53, TBP, 5915, RARG, RARA, JUND, JUNB, FOSB, FOS, AR, PDCD11, hsa-miR-135a, hsa-miR-135b, hsa-miR-202, hsa-miR-326, hsa-miR-330-5p, hsa-miR-544
S100A9	TFAP2A, TP53, TBP, SPI1, 5915, RARG, RARA, MYB, CTCF, AR, hsa-miR-196a
SERPINE1	USF1, TFAP2A, MYC, JUN, USF2, TP53, TFAP2C, TBP, SREBF2, SREBF1, SP1, SMAD4, SMAD3, SMAD1, RXRB, 5915, RXRA, PPARG, PPARD, PPARA, POU3F2, NFIC, NFE2L1, MXI1, 23601, MAX, JUND, JUNB, HIF1A, GABPA, FOXA2, FOSB, FOS, ETS1, ESR2, ESR1, EPAS1, EGR1, E2F3, E2F2, E2F1, AR, hsa-miR-143, hsa-miR-181b, hsa-miR-181d, hsa-miR-204, hsa-miR-224, hsa-miR-301a, hsa-miR-30a, hsa-miR-30b, hsa-miR-30c, hsa-miR-30d, hsa-miR-30e, hsa-miR-34a, hsa-miR-34c, hsa-miR-99a
SOD1	TFAP2A, NFKB1, MYC, JUN, WT1, TBP, STAT6, STAT4, STAT3, STAT2, STAT1, SP1, NFYA, NFIC, NFE2, HNF4A, EGR1, CEBPB, CEBPA, BACH2, ARNT, AHR, HOXB2, RNF19A, hsa-miR-1, hsa-miR-206, hsa-miR-217, hsa-miR-377, hsa-miR-613
IL-8	TFAP2A, RELA, NFKB2, NFKB1, MYC, JUN, U2AF1, TP53, TFAP2C, REL, RARA, POU2F1, MAPK9, MAPK8, MAPK3, MAPK14, MAPK13, MAPK12, MAPK11, MAPK10, MAPK1, FOS, ETV4, ETS1, ESR1, CEBPB, CEBPA, AR, IRF5, hsa-miR-146a, hsa-miR-17, hsa-miR-204, hsa-miR-20a, hsa-miR-302a, hsa-miR-302b, hsa-miR-302c, hsa-miR-302d, hsa-miR-372, hsa-miR-520a-3p, hsa-miR-520b, hsa-miR-520d-3p, hsa-miR-520e, hsa-miR-93

### Prediction of candidate drugs

We made predictions about possible effective intervention drugs with the use of the Enrichr platform, which is based on the DSigDB database. The top 10 potential chemical compounds are extracted based on their adjusted *p*-value (**Table 6**). The results showed that phorbol 12-myristate 13-acetate (CTD 00006852) and dexamethasone (CTD 00005779) are the two drug molecules that interacted with most genes.

**Table 6:** Prediction of top 10 candidate drugs for Long COVID and ME/CFS.

Name of drugs	*p*-Values	Adjusted *p*-value	Genes
Phorbol 12-myristate 13-acetate CTD 00006852	9.63E−13	1.42E−09	CXCL8, EGF, SERPINE1, BCL2, HMGB1, S100A9, S100A8, SOD1
Dexamethasone CTD 00005779	6.05E−11	4.45E−08	CXCL8, EGF, SERPINE1, BCL2, B2M, S100A9, S100A8
Palmitic acid CTD 00007272	1.11E−09	5.42E−07	CXCL8, SERPINE1, BCL2, SOD1
Pyrrolidine dithiocarbamate CTD 00001021	3.66E−09	1.17E−06	CXCL8, EGF, BCL2, HMGB1, SOD1
Lactacystin CTD 00002698	3.97E−09	1.17E−06	CXCL8, SERPINE1, BCL2, SOD1
CALCIUM CTD 00005559	6.46E−09	1.58E−06	CXCL8, EGF, S100A9, S100A8, SOD1
COPPER CTD 00005706	2.11E−08	4.43E−06	CXCL8, EGF, SERPINE1, BCL2, HMGB1, B2M, S100A8, SOD1
SILVER CTD 00006742	3.59E−08	6.15E−06	CXCL8, SERPINE1, BCL2, SOD1
TPEN CTD 00001994	3.76E−08	6.15E−06	CXCL8, BCL2, S100A9, S100A8, SOD1
PD 98059 CTD 00003206	4.48E−08	6.59E−06	CXCL8, EGF, SERPINE1, BCL2, SOD1

ME/CFS, myalgic encephalomyelitis/chronic fatigue syndrome.

## Discussion

Several studies have shown that a large proportion of patients with SARS-CoV-2 infection will experience a series of symptoms such as fatigue, dyspnea, and sleep difficulties after acute onset, which is called Long COVID (5, 63–66). Cardinal clinical features manifested by individuals with Long COVID present analogously to ME/CFS, which is also known as a post-infectious syndrome caused by many types of infectious agents (67). Given the substantial public health burden that Long COVID and ME/CFS could impose, increasing investment and proactive advances in potential mechanisms are in urgent need considering that the optimal therapeutic regimen’s current status is undetermined (68–73). In this study, we identified genes associated with Long COVID and ME/CFS. Afterward, we performed a series of bioinformatics analyses grounding on nine common genes that we found between Long COVID and ME/CFS.

The nine identified common genes were applied for detecting GO terms. Regarding the GO biological process terms, peptidyl-cysteine *S*-nitrosylation, leukocyte aggregation, peptidyl-cysteine modification, positive regulation of intrinsic apoptotic signaling pathway, and response to iron ion are the most significant. Among them, leukocyte aggregation is intimately related to microvascular plugging (74). Leukocyte aggregation to platelets has been demonstrated by post-mortem studies of deceased patients with COVID-19 (75). However, whether the widespread vascular dysfunction is a potential cause of neurological deterioration in COVID-19 patients remains uncertain. As for the molecular function, the top GO terms are as follows: RAGE receptor binding, arachidonic acid binding, and icosatetraenoic acid binding. RAGE, presenting on the surface of various cell types in atherosclerotic lesions, is a multiligand transmembrane receptor in the immunoglobulin superfamily (76–78). The binding of RAGE to advanced glycation end products (AGEs) plays an important role in the development of late atherosclerosis complications in diabetes (79–81). The dense linkage and underlying mechanisms among COVID-19, atherosclerosis, and diabetes have been shown in the relevant literature (82–84). In terms of cellular components, the early endosome lumen and MHC class I protein complex rank the top 2. β2-Microglobulin is the essential conformation of the MHC class I protein complex, the level of which acts as an early indicator for disease severity and outcome prediction of COVID-19 (85).

The KEGG pathway enrichment analysis was performed to identify the common pathway of Long COVID and ME/CFS. The top 10 KEGG Human pathways include bladder cancer, IL-17 signaling pathway, AGE-RAGE signaling pathway in diabetic complications, HIF-1 signaling pathway, p53 signaling pathway, colorectal cancer, prostate cancer, Chagas disease, NF-kappa B signaling pathway, and base excision repair. IL-17, a member of the multifunctional cytokine family, serves as both a severity biomarker of COVID-19 and a promising therapeutic target to mitigate the lung damage of COVID-19 (86–88). Intriguingly, as a key modulator of upstream inflammatory pathways, IL-17 contributes to the production of IL-6, the elevated peripheral levels of which may initiate ME/CFS through neuroinflammation (6). Meanwhile, results from WikiPathways show that most interacted gene pathways are extracellular vesicles in the crosstalk of cardiac cells and the overview of nanoparticle effects. Results from BioCarta and Reactome separately produce CBL-mediated ligand-induced downregulation of EGF receptors *Homo sapiens* h-cbl Pathway and BH3-only proteins associate with and inactivate anti-apoptotic BCL-2 members *Homo sapiens* R-HSA-111453.

According to the PPI network, IL-6, IL-1B, CD8A, TP53, and CXCL8 were declared as hub proteins on account of their high degrees. On the one hand, IL-6, as a multifunctional molecule, plays a crucial role in COVID-19-related hyperinflammation (89), which may lead to ME/CFS through neuroinflammation (90, 91). On the other hand, as an energy distributor in muscle tissue (92), IL-6 may get involved in the occurrence of ME/CFS through related pathways of energy metabolism (93). The results of the study showed that the levels of inflammatory cytokines such as IL-6, IL-10, and TNF-α increased in ME/CFS patients, suggesting that ME/CFS patients were in a low-grade systemic inflammatory state for a long time (94). In infection-triggered ME/CFS, IL-1b release is inversely correlated with sCD26 expression (95), while the expression level of sCD26 has been linked to health-related quality of life (96). IL-1b was associated with the altered regulation of several genes involved in the myogenic processes, elucidating the mechanism of muscle loss in COVID-19 (97). A substantial reduction of CD8A was found in spleen autopsy specimens from patients who died of COVID-19 (98). In addition to being known as a tumor suppressor, the P53 protein, encoded by the TP53 gene, is a crucial component of the body’s antiviral response (99). The higher expression level of CXCL8, a chemokine also known as IL-8, was correlated with greater clinical severity of COVID-19 (100). Interestingly, the levels of CXCL8 showed a decrease of 42% in CFS patients, compared with control subjects (101).

Transcription factors leverage a prominent role in regulating gene expression. From the TF–gene interaction network, SERPINE1 and BCL2 showed a high interaction rate with other TF genes. Among them, SERPINE1 is regulated by 57 TF genes, and BCL2 is regulated by 54 TF genes. SERPINE1, a member of the Serpin family of proteins, prevents the formation of plasmin and inhibits fibrinolysis and blood clot dissolution (102), contributing to coagulopathy associated with COVID-19. Evidence suggests that all COVID-19 patients regardless of disease severity have elevated levels of SERPINE1 (103). In addition, SERPINE1 impedes the regeneration of skeletal muscle strength (104). Among the regulators, CEBPG, KLF8, and WRNIP1 were the regulators with the highest degree in the TF–gene interaction network, all of which were involved in the expression of three common genes.

TFs and miRNAs not only can co-regulate the expression of target genes but also can mutually regulate each other, standing out as a pivot in various biological processes and different diseases (105). By complying TF–miRNA coregulatory network, our analysis revealed the relationship between shared genes, TFs, and miRNAs. Among the identified transcription factors, IL-8 showed the highest degree at 43. As a member of the cytokine storm caused by COVID-19 (106), IL-8 is closely related to the severity and prognosis of the disease (107). IL-8 is expressed at high levels for ME/CFS in the recently afflicted, and the adjustment of IL-1α, IL-6, and IL-8 for illness duration may serve as powerful biomarkers for screening ME/CFS (108). Among the identified MicroRNAs, hsa-miR-204 showed the highest degree at 3. As a regulator of gene expression, miR-204 may engage in mediating the expression of neurotransmitter and ion channel-related gene sets by regulating non-coding RNAs (ncRNAs) (109).

By screening the DSigDB database, nine common genes were applied to predict candidate drugs, and the top 10 significant drugs were highlighted. Phorbol 12-myristate 13-acetate (TPA), used for the treatment of various tumors, can induce the differentiation or apoptosis of various cell lines at low concentrations (110). Dexamethasone, a synthetic adrenal corticosteroid that exerts anti-inflammatory and immunosuppressive effects through the glucocorticoid receptor (GR), was demonstrated to relieve inflammation in COVID-19 by pro-resolving lipid mediators (111). Decreased hypothalamic–pituitary–adrenal (HPA) axis function in patients with chronic fatigue syndrome (CFS) suggests that CFS is associated with hypocortisolism (112). Lactacystin, a component of the ubiquitin–proteasome complex degrading unnecessary cellular proteins, shows the influence on mitochondrial metabolism *via* modulation of reactive oxygen species (ROS) and glutathione (GSH) (113), which also get involved in sleep homeostasis and CFS development (114).

This study included several limitations, which should be acknowledged and taken into consideration. On the one hand, the studies of Long COVID and ME/CFS remained inadequate so far as compared with abundant data in the acute phase of COVID-19, causing the paucity of available datasets for the aforementioned diseases. On the other hand, the differential expression gene about ME/CFS in multiple datasets, including both RNA sequencing data and microarray data, is absent, which may be due to the shortcomings of previous study designs as well as the attribute of the disease itself. All of the abovementioned causes eventually limited the use of the additional datasets in this study. Furthermore, since our study is based on pure bioinformatics analysis without clinical substantiation, the result including the biological functions and the enrichment analysis of hub genes as well as *in vivo* safety and efficacy of candidate drugs needs to be validated by further experimental exploration and clinical trials.

## Conclusion

Our study found the common genes between Long COVID and ME/CFS. Long COVID and ME/CFS show relative similarities in infection, neuroinflammation, energetic metabolic dysfunction, and impaired immune function by a series of bioinformatics analyses. The long-term health consequences of COVID-19 are not to be neglected, and dexamethasone may treat patients with Long COVID and ME/CFS by modulating the HPA axis, although the predicted results still need to be rigorously validated by experiments.

## Data availability statement

Publicly available datasets were analyzed in this study. This data can be found here: https://www.ncbi.nlm.nih.gov/geo/query/acc.cgi?acc=GSE169687.

## Author contributions

YL, TZ conceived and designed this research. YL carried out the data analysis and data interpretation. TZ is responsible for literature searching on the background of this disease and the image processing. JC and SZ wrote most of the article. CH annotated the picture and wrote the conclusion. JL reviewed and revised the manuscript. All authors contributed to the article and approved the submitted version.

## Funding

This research was funded by grants from the Guangzhou Municipal Science and Technology Project, Guangdong, China (Grant No. 201803010053).

## Acknowledgments

We are grateful to the researchers who built the public database and shared an enormous amount of research data, which made our study possible through their generous contributions.

## Conflict of interest

The authors declare that the research was conducted in the absence of any commercial or financial relationships that could be construed as a potential conflict of interest.

## Publisher’s note

All claims expressed in this article are solely those of the authors and do not necessarily represent those of their affiliated organizations, or those of the publisher, the editors and the reviewers. Any product that may be evaluated in this article, or claim that may be made by its manufacturer, is not guaranteed or endorsed by the publisher.
