# Supplementary material for: Bioinformatics and systems biology approach to identify the pathogenetic link of Long COVID and Myalgic Encephalomyelitis/Chronic Fatigue Syndrome
Source: Front Immunol. 2022 Sep 16;13:952987. doi: 10.3389/fimmu.2022.952987 (PMC9524193; doi:10.3389/fimmu.2022.952987)
Supplement: Supplementary file 1 [file DataSheet_1.pdf]

DEGs at 12 weeks post-infection relative to controls					
**external_gene_name**	**logFC**	**logCPM**	**F**	**PValue**	**FDR**
HMGB1P6	0.926780129	2.574281538	37.03934785	9.23E-09	7.50E-05
RBPMS2	-2.62110337	1.489288608	36.82383175	1.01E-08	7.50E-05
ERH	0.843511102	3.639242711	33.99398776	3.27E-08	0.000161984
BLOC1S2	0.693601085	4.087503436	33.18732395	4.58E-08	0.000170479
RPLP0P6	0.855887151	3.59036086	32.4186272	6.34E-08	0.000188652
RPL41	1.626485715	6.72542128	30.90989755	1.20E-07	0.000244967
B2M	0.777457577	11.39888057	30.24744691	1.60E-07	0.000244967
TMX1	0.583934127	4.993232828	30.20926991	1.62E-07	0.000244967
PRDX1	0.59361138	4.992866767	30.16702248	1.65E-07	0.000244967
NDUFS5	1.086942066	3.56772415	29.99747286	1.78E-07	0.000244967
HMGB1P5	0.929385232	3.65600296	29.95697415	1.81E-07	0.000244967
USP1	0.449394036	5.444246265	29.3461023	2.36E-07	0.000292127
TMEM70	0.477115418	3.524827948	29.02772232	2.70E-07	0.000309413
UFC1	0.561494622	4.507638588	28.76184201	3.03E-07	0.000322351
RPS23	1.124696535	7.777783366	28.45468462	3.46E-07	0.000343724
AC090220.1	-0.56007218	2.032680178	28.30585642	3.70E-07	0.000343757
SS18L2	0.684065483	2.620862647	28.07883253	4.08E-07	0.000357103
AC099336.2	1.921605952	2.200403514	27.75769411	4.69E-07	0.000370329
SCARNA9	1.018303292	3.24112427	27.7227205	4.76E-07	0.000370329
MYL12B	0.553529239	6.830898069	27.6224839	4.98E-07	0.000370329
ZNF765	0.704434564	4.118063932	27.30671375	5.71E-07	0.000404876
ZNF845	0.722766581	4.438570259	27.05125759	6.39E-07	0.00042143
DNTTIP2	0.636077266	4.810240264	27.00338548	6.52E-07	0.00042143
PHB	0.346619114	4.366875719	26.81763918	7.08E-07	0.00042143
RPL7	1.782467724	7.20424667	26.75983737	7.26E-07	0.00042143
NDUFS3	0.411207843	3.647561859	26.56549246	7.91E-07	0.00042143
TMCO1	0.59220919	4.868060281	26.45083929	8.32E-07	0.00042143
RASGRF1	3.55118421	1.968701039	26.25961158	9.05E-07	0.00042143
RPS3A	1.90318612	7.479813137	26.22369655	9.19E-07	0.00042143
ACAT1	0.600453121	3.433181245	26.19330131	9.32E-07	0.00042143
EEF1B2	1.627542615	5.581212489	26.18598632	9.35E-07	0.00042143
AC245060.5	1.872198562	3.238469297	26.07824126	9.80E-07	0.00042143
MRPL47	0.738178541	2.75151606	26.01568206	1.01E-06	0.00042143
HMGB1	0.582479113	6.811912093	25.99237241	1.02E-06	0.00042143
SNRPD2	1.04336329	4.322332931	25.98882657	1.02E-06	0.00042143
GSKIP	0.579287315	3.881474565	25.80678273	1.10E-06	0.000439486
DUT	0.547837661	5.037869813	25.73304111	1.14E-06	0.000439486
PNPLA8	0.467214819	5.196192347	25.71269852	1.15E-06	0.000439486
EEF1D	0.370642677	8.243789983	25.57538541	1.22E-06	0.000455339
COMMD8	0.924185425	2.739747389	25.45424506	1.29E-06	0.000466413
ZNF721	0.717870226	5.382499824	25.37678667	1.34E-06	0.000466413
TOMM7	1.279300224	4.668448919	25.35788765	1.35E-06	0.000466413
METTL18	0.995950213	1.701235486	25.20098902	1.44E-06	0.000488665
SNHG29	0.756748887	5.837859636	25.08830547	1.52E-06	0.000502319
MRPL50	0.718870762	3.436685395	24.91027148	1.64E-06	0.000531861
RPL39	1.701439595	4.66441557	24.61413562	1.88E-06	0.000593901
RPA3	0.601512946	2.549968814	24.46401411	2.01E-06	0.000610596
EEF1A1	0.821283552	11.75666527	24.44097045	2.03E-06	0.000610596
SGCE	1.949492305	1.18610936	24.41323315	2.05E-06	0.000610596
RPL3	0.693026877	9.435438186	24.27935671	2.18E-06	0.000635498
SF3B6	0.852470883	3.847859724	24.1838384	2.27E-06	0.000650458
RPS7	1.576629085	6.424591687	24.10233025	2.36E-06	0.000661397

RSL24D1	1.059974952	4.45901252	24.06215823	2.40E-06	0.000661397
HSP90AA1	0.937418702	7.524365234	23.9024141	2.58E-06	0.000691682
RPL7P1	1.79127517	4.141883864	23.87078514	2.61E-06	0.000691682
RPL23	1.637746971	6.902863531	23.77380576	2.73E-06	0.000691682
RPL6	0.96403437	7.737542293	23.77272481	2.73E-06	0.000691682
RPL26L1	0.981320036	1.508331332	23.75372915	2.76E-06	0.000691682
ACTR6	0.652789967	3.233957464	23.72700634	2.79E-06	0.000691682
TMSB4X	0.328182909	9.614830591	23.67713035	2.85E-06	0.000695736
RPL7P9	1.789757668	4.556250277	23.55920332	3.01E-06	0.000721723
RPL9	1.457465922	6.699960312	23.42439101	3.19E-06	0.00072389
UQCRB	1.365008683	4.743208687	23.41621472	3.21E-06	0.00072389
S100A8	1.842383195	8.381231209	23.40876519	3.22E-06	0.00072389
MIR3609	1.582159454	3.270335753	23.40450846	3.22E-06	0.00072389
RPL21	1.418656662	7.290731203	23.3799249	3.26E-06	0.00072389
ZCCHC10	0.691247782	3.351720328	23.22481929	3.49E-06	0.000764792
OTUD6B-AS1	0.78162766	4.400315807	23.10605931	3.69E-06	0.000791166
ATP5PF	0.68580611	3.66489127	23.0851727	3.72E-06	0.000791166
RPL41P1	1.735597361	0.899833344	22.70296804	4.42E-06	0.000919857
DNAJA1	0.620871739	6.08875305	22.68876101	4.45E-06	0.000919857
RPL11	1.186140159	7.855884066	22.60290119	4.63E-06	0.000943163
RPS7P1	1.839327363	3.590916232	22.39176989	5.09E-06	0.000988186
RPS27	1.480939551	8.501661424	22.38663913	5.10E-06	0.000988186
RPL26	1.898773874	5.314117273	22.36554589	5.15E-06	0.000988186
GTF2B	0.423397659	4.552035022	22.25306084	5.42E-06	0.001021117
N4BP2L2-IT2	-0.35141416	5.571256468	22.13980275	5.71E-06	0.001058957
EIF3E	0.774749193	6.630297906	22.10029301	5.81E-06	0.001058957
CIAO2A	0.59005238	4.026588755	22.08480695	5.85E-06	0.001058957
IGBP1	0.479822671	4.89203383	22.06399281	5.91E-06	0.001058957
RPS3AP6	1.978972791	1.322267143	22.0306372	6.00E-06	0.001062221
MT-ATP8	-0.78357169	6.823127563	22.00478168	6.07E-06	0.001062221
PHF5A	0.555491866	3.460053942	21.84704719	6.52E-06	0.001108204
HAT1	0.564702623	4.073665385	21.84478193	6.52E-06	0.001108204
SNRPE	0.827217859	3.045303983	21.80472586	6.64E-06	0.001108204
ZNF92	0.842834965	3.989527971	21.78582286	6.70E-06	0.001108204
ATP6V1G1	0.55904764	5.862541408	21.7381884	6.85E-06	0.001108662
RDH14	0.601877256	2.991085492	21.72346497	6.89E-06	0.001108662
TOMM20	0.357271236	5.445715624	21.69964682	6.97E-06	0.001108662
BX679664.3	1.947343474	2.15790025	21.67437966	7.05E-06	0.001108662
LINC00909	0.465406195	4.379481994	21.66613051	7.08E-06	0.001108662
AL021707.5	-0.80950478	1.384167676	21.56480664	7.41E-06	0.001135838
AP000547.3	-1.4078736	3.574525845	21.54599827	7.48E-06	0.001135838
CD69	0.891303813	3.263200127	21.50028522	7.63E-06	0.001135838
AC125612.1	-0.46748685	1.957748489	21.48440326	7.69E-06	0.001135838
LARP7	0.573590969	4.614246916	21.47859444	7.71E-06	0.001135838
HSPA12A	2.064348573	2.436508337	21.45576879	7.79E-06	0.001136471
AC116533.1	2.256758536	4.092635702	21.37818558	8.07E-06	0.001150894
RPL34	1.94701131	5.993515751	21.36981366	8.10E-06	0.001150894
KIF20B	0.639600137	4.069383335	21.36459742	8.12E-06	0.001150894
AK6	0.870531183	1.259206067	21.25922999	8.52E-06	0.001196226
TXNDC17	0.553075822	2.886749554	21.22955785	8.64E-06	0.001198812
POLR2K	1.085233557	2.385383145	21.20904761	8.72E-06	0.001198812
DCLK2	2.524567354	1.066717462	21.17080951	8.87E-06	0.001198812
IFT57	0.759771756	3.748963144	21.16991492	8.88E-06	0.001198812
TMA7	1.097349578	4.090165755	21.14291307	8.99E-06	0.001198812

WDR89	0.715169015	3.585722224	21.12053724	9.08E-06	0.001198812
SSB	0.689681478	4.569709372	21.11187938	9.11E-06	0.001198812
CCT2	0.605966144	4.920243352	21.07787452	9.26E-06	0.001198812
ZNF146	0.467022296	5.401183107	21.07622539	9.26E-06	0.001198812
PSMC6	0.510523085	4.984440064	21.00548036	9.57E-06	0.001227577
AC092620.1	-0.71429579	2.694613855	20.97314569	9.71E-06	0.001228754
SCARNA7	1.636501863	5.980954278	20.95324154	9.80E-06	0.001228754
ATP5PB	0.498368367	5.528006826	20.94761119	9.83E-06	0.001228754
PPIA	0.532275041	7.665195873	20.92311481	9.94E-06	0.00123226
RPS7P11	1.809461046	1.274870915	20.87322574	1.02E-05	0.001250324
GPT2	1.890652169	2.014704728	20.84217911	1.03E-05	0.001254853
AC010343.1	0.85082806	2.829917259	20.82955706	1.04E-05	0.001254853
SAMD9	0.718374476	7.505867782	20.69880329	1.10E-05	0.00131107
MRPS33	0.740144518	2.808733526	20.67958118	1.11E-05	0.001312186
SLU7	0.410324865	5.748819647	20.6417594	1.13E-05	0.001323332
AC115223.1	1.117343565	1.199959031	20.62683563	1.14E-05	0.001323332
CAMLG	0.363698974	4.322836211	20.49994189	1.21E-05	0.001381688
TIMM8B	0.88696733	2.943052621	20.49910995	1.21E-05	0.001381688
PNRC2	0.413058211	6.623149364	20.45306692	1.23E-05	0.001383678
TDRD9	-1.09583381	2.378681442	20.45127845	1.23E-05	0.001383678
BTN2A3P	-0.77192673	2.650491835	20.44633439	1.24E-05	0.001383678
SMIM19	0.437216898	2.809066589	20.37957112	1.28E-05	0.001412089
SELENOF	0.341459729	5.792209983	20.36966901	1.28E-05	0.001412089
RPL31	1.703313373	6.925107753	20.34986053	1.29E-05	0.001414535
RPL13AP5	0.709809713	5.408533355	20.30255569	1.32E-05	0.001424112
RRN3P2	-0.50340257	2.576569363	20.29971535	1.32E-05	0.001424112
PFDN2	0.516277698	3.009184092	20.28777674	1.33E-05	0.001424112
ZNF268	0.592999082	4.265631582	20.25626632	1.35E-05	0.001425183
AC007390.1	0.652835882	2.250957418	20.23975576	1.36E-05	0.001425183
ZNF816	0.634950011	3.396378476	20.21180828	1.38E-05	0.001433546
MRPL51	0.684111631	3.439173586	20.18611501	1.39E-05	0.001440536
ANKRD12	0.437599217	7.948693009	20.16807552	1.41E-05	0.001442539
DNAJC8	0.420917048	5.220834395	20.13708112	1.43E-05	0.001453263
TMSB10	0.504758058	8.454753283	20.1056665	1.45E-05	0.001464425
RPS9	0.486400515	8.076118135	20.08428776	1.46E-05	0.001468934
NMD3	0.530167001	4.310545746	20.04465868	1.49E-05	0.001480619
NDUFA4	0.775216713	3.981898303	20.02523346	1.50E-05	0.001480619
RPL13A	0.644376006	9.917377292	20.01295838	1.51E-05	0.001480619
RBAK	0.553206091	4.820194347	19.99208287	1.52E-05	0.001480619
NDUFA5	0.586489675	4.069596614	19.96771229	1.54E-05	0.001480619
RIOK2	0.542246436	3.841893298	19.96711329	1.54E-05	0.001480619
MT-ATP6	-0.67848124	10.37492861	19.95374977	1.55E-05	0.001480619
COPS4	0.57396179	3.5833884	19.95296483	1.55E-05	0.001480619
TRIAP1	0.740310077	1.856193365	19.90346566	1.59E-05	0.001502963
MRPS15	0.325908064	3.828828866	19.88157397	1.60E-05	0.001502963
TAF7	0.589545833	6.315723235	19.87648972	1.61E-05	0.001502963
H4C3	0.887392813	5.376836215	19.86566898	1.62E-05	0.001502963
SRP14	0.657444991	5.825832939	19.85161992	1.63E-05	0.00150335
PSMC2	0.483256603	4.313319273	19.81784161	1.65E-05	0.001517562
CHN2	-0.61105112	3.377497266	19.80424756	1.66E-05	0.001517753
COX7B	0.805198393	3.480533591	19.72610573	1.72E-05	0.001552482
PFDN5	1.118260556	6.04815125	19.71791101	1.73E-05	0.001552482
MRFAP1L1	0.426827278	5.591060763	19.69617663	1.75E-05	0.001552482
NDUFA1	0.884012325	3.597163198	19.66653799	1.77E-05	0.001552482

AC009093.7	-0.58087972	2.921317576	19.65899782	1.78E-05	0.001552482
MED7	0.39950734	3.737414623	19.65361139	1.78E-05	0.001552482
TVP23B	0.342768984	4.2124175	19.65165872	1.78E-05	0.001552482
DYNLT3	0.672411828	3.421856282	19.62121007	1.81E-05	0.001565351
CELSR1	1.813555229	4.024413895	19.56900891	1.85E-05	0.001585192
LTF	2.743015291	5.397398321	19.51967757	1.90E-05	0.001612544
MIR155HG	1.074214743	1.122036203	19.48010972	1.93E-05	0.001630961
PEG10	1.785271099	1.712560122	19.47061915	1.94E-05	0.001630961
CAPZA1	0.47487371	7.915943291	19.43122412	1.98E-05	0.001651671
MMP8	2.965771058	2.620503185	19.3978724	2.01E-05	0.001655903
HAUS1	0.576191347	2.733205541	19.367268	2.03E-05	0.001655903
CENPQ	0.839972207	1.142248237	19.35475563	2.05E-05	0.001655903
MMADHC	0.32240659	4.930415417	19.35417864	2.05E-05	0.001655903
RPL4	0.641952598	9.213623018	19.32769145	2.07E-05	0.001667305
RNU4ATAC	1.807879425	1.974580433	19.31347178	2.09E-05	0.001669314
IGFBP4	1.885115468	2.622722578	19.28806936	2.11E-05	0.001676455
ARPC3	0.421844861	7.089576584	19.28121226	2.12E-05	0.001676455
ATP5ME	0.752894111	3.554364364	19.21814088	2.18E-05	0.001713923
PYURF	0.477301996	4.558424166	19.2107773	2.19E-05	0.001713923
GON7	0.606776198	2.132980806	19.1111742	2.29E-05	0.00178141
ZNF271P	0.346965787	4.412198523	19.10506555	2.30E-05	0.00178141
ANXA1	0.770443008	7.034001691	19.06513555	2.34E-05	0.001804016
IGLV1-47	-1.42827425	1.504205745	19.04501339	2.36E-05	0.001804016
PSMA3	0.417373327	4.24986964	19.03496281	2.37E-05	0.001804016
IGHG4	-1.61724769	2.714317952	19.00215684	2.41E-05	0.001804016
VBP1	0.590090697	3.679568547	18.99338943	2.42E-05	0.001804016
RPL21P16	1.460997032	5.103807357	18.99087592	2.42E-05	0.001804016
ZNF480	0.654913602	4.252913071	18.98975801	2.42E-05	0.001804016
DBI	0.842011321	4.177015099	18.98313963	2.43E-05	0.001804016
RPL35	0.987129929	6.993331957	18.97938454	2.44E-05	0.001804016
KRCC1	0.506439506	4.532636428	18.95656595	2.46E-05	0.001814238
RPS27L	0.560172859	3.656580464	18.93675828	2.49E-05	0.001821714
KBTBD8	0.983747286	3.275631083	18.92590595	2.50E-05	0.001821714
ZNF830	0.359777386	4.120812068	18.91603793	2.51E-05	0.001821714
RPL24P4	0.97423049	2.84690179	18.8965076	2.53E-05	0.001829163
NUP37	0.456960155	2.810092387	18.88640519	2.54E-05	0.001829163
RPL24	0.868297191	6.541191963	18.84763647	2.59E-05	0.001850295
PLRG1	0.421621642	4.555200594	18.84083972	2.60E-05	0.001850295
H4C2	0.794581389	4.186706134	18.70244139	2.77E-05	0.001955025
EEF1A1P5	0.745446644	7.201983178	18.69137207	2.79E-05	0.001955869
MRPL48	0.477765102	2.854281325	18.67793162	2.80E-05	0.001958922
SMIM30	0.896845641	1.874808984	18.65204914	2.84E-05	0.001973441
RPS3AP26	1.865843122	2.893294812	18.6184078	2.88E-05	0.001995323
PPIG	0.533163143	6.024874322	18.60527745	2.90E-05	0.001998287
COX7C	1.092182002	4.747311352	18.54494439	2.98E-05	0.002036485
CLIP2	-0.50721486	4.611848468	18.51995051	3.02E-05	0.002041651
THAP5	0.417821237	5.217722258	18.50955703	3.03E-05	0.002042299
MTND5P14	-0.55299413	2.89552947	18.47146546	3.09E-05	0.002065826
EEF1DP7	-0.4358863	2.983198829	18.46573264	3.10E-05	0.002065826
SNRPG	0.696499237	3.613738067	18.42253339	3.16E-05	0.002098525
TOMM22	0.325356572	4.577538171	18.33986914	3.28E-05	0.00217149
SRP9	0.450561921	5.579772159	18.32952554	3.30E-05	0.002172361
RANBP6	0.561709855	4.715674934	18.29514659	3.35E-05	0.002197842
NDUFB3	1.072988768	3.052139735	18.22836995	3.46E-05	0.002257638

RPL30	0.796021723	7.902191352	18.12787837	3.63E-05	0.002356051
CD52	0.802031952	7.211695234	18.07830188	3.71E-05	0.002394574
COPDA1	2.305701339	1.85314941	18.07358979	3.72E-05	0.002394574
ENY2	0.340501528	4.492054974	18.0654741	3.73E-05	0.002394574
MYL6	0.591978708	7.420269411	18.05127019	3.76E-05	0.00240022
MDH1	0.439915987	4.831541434	18.01765822	3.82E-05	0.002425183
SNORD13	1.165215912	0.728015785	18.01096124	3.83E-05	0.002425183
OBI1	0.55902809	4.078003204	17.97829028	3.89E-05	0.002452183
TRMT10C	0.636992446	3.169908261	17.96207587	3.92E-05	0.002460475
AC010615.1	0.859256229	1.73240342	17.95110456	3.94E-05	0.002462776
AC099560.2	1.434841917	4.064017343	17.93047219	3.98E-05	0.002476322
ZNF208	1.27893453	2.609068837	17.90913723	4.02E-05	0.002481413
MRPL40	0.61476138	2.033264739	17.90833226	4.02E-05	0.002481413
NDUFA6	0.631249231	3.612790595	17.83530789	4.16E-05	0.002540646
COMMD6	1.192683851	3.831179191	17.82322536	4.18E-05	0.002540646
ZNF525	0.702248935	4.148412754	17.81413596	4.20E-05	0.002540646
SUB1	0.429857736	6.09908609	17.81306316	4.20E-05	0.002540646
AC011676.1	-0.76893752	2.690705261	17.80600656	4.22E-05	0.002540646
UFM1	0.452200774	5.184399642	17.80568813	4.22E-05	0.002540646
RPS24	1.242614484	6.971741523	17.76906181	4.29E-05	0.002569675
SCARNA21	0.869337182	7.179194953	17.7643295	4.30E-05	0.002569675
RPSA	0.66442912	8.009834343	17.73971139	4.35E-05	0.00258917
GPM6A	2.450149852	1.298871312	17.71527603	4.40E-05	0.002589338
LSM3	0.666291923	3.158683586	17.71066964	4.41E-05	0.002589338
NSMCE1	0.354644788	4.184654987	17.70588171	4.42E-05	0.002589338
CRISP3	2.309976886	1.877184718	17.7057958	4.42E-05	0.002589338
RPS25	0.910070229	7.092475491	17.69102859	4.45E-05	0.002590047
XRRA1	-1.09760099	4.837019933	17.68852685	4.46E-05	0.002590047
CAMP	1.887787529	3.265588997	17.62484122	4.59E-05	0.0026567
LRRC37A15P	-0.55689483	1.761399989	17.61794	4.61E-05	0.0026567
ZNF420	0.510798405	3.382383254	17.58765827	4.67E-05	0.00268441
DEK	0.387186875	6.594755542	17.54557287	4.77E-05	0.00272757
EIF4A2	0.385829559	7.48467595	17.53490424	4.79E-05	0.002730798
RPL4P4	0.728545742	1.771866278	17.51308221	4.84E-05	0.002739275
RPL23AP42	0.534191186	4.595670019	17.51210344	4.84E-05	0.002739275
MT-ND1	-0.73614561	10.94969679	17.49692172	4.88E-05	0.002744397
CHMP5	0.573042283	4.559798768	17.49204455	4.89E-05	0.002744397
CBX3	0.432139935	5.582854245	17.48306172	4.91E-05	0.002745669
C11orf80	0.935343177	3.338343201	17.46559481	4.95E-05	0.002757979
IFT20	0.407191181	3.28551363	17.43909029	5.01E-05	0.002782206
AC087284.1	-0.4416193	3.705506753	17.39447385	5.12E-05	0.002819341
CD24	1.179161075	4.075721394	17.38341861	5.14E-05	0.002819341
JRKL	0.548063881	3.528311333	17.38066179	5.15E-05	0.002819341
RPS27A	0.966722524	7.541517788	17.37950855	5.15E-05	0.002819341
BRIX1	0.510231241	3.20811246	17.37013295	5.18E-05	0.002821455
AC008038.1	0.527239292	6.480178981	17.26485941	5.44E-05	0.002929176
RPL35A	0.893527124	6.774043314	17.26094404	5.45E-05	0.002929176
ZNF781	0.782494644	1.576418898	17.25884769	5.45E-05	0.002929176
FO393411.1	1.760637341	0.908258257	17.24850064	5.48E-05	0.002929176
RPS6	0.889477228	8.413634358	17.24640034	5.49E-05	0.002929176
ATG5	0.407795391	4.502876476	17.24458591	5.49E-05	0.002929176
PFDN4	0.757592631	1.99681016	17.23031041	5.53E-05	0.002938443
COX6C	0.807126328	4.026649505	17.17368626	5.68E-05	0.003007301
BDH2	0.702181412	2.225493039	17.13285372	5.79E-05	0.00305498

UQCRH	0.684433752	3.992404572	17.03968334	6.05E-05	0.003181194
SNORD3A	2.351479969	6.104039881	17.02896321	6.08E-05	0.003186098
SNX4	0.527894305	3.804904978	16.99894399	6.17E-05	0.003212716
CEBPZ	0.555185243	4.864749215	16.98651131	6.20E-05	0.003216717
ATP6V1D	0.353399322	4.43855643	16.95278774	6.30E-05	0.003257089
NUDCD1	0.602409555	3.516056126	16.92354967	6.39E-05	0.00329103
RPS20	0.889152021	7.661248579	16.90321029	6.45E-05	0.0033114
RPL22L1	0.943448286	2.508653762	16.88971331	6.49E-05	0.003321169
GIMAP2	0.473371362	5.945186381	16.87586859	6.54E-05	0.003321335
MT-ND4L	-0.65033198	8.609928146	16.87513731	6.54E-05	0.003321335
ZNF594	0.620418102	4.468267368	16.86040242	6.58E-05	0.003333208
PSMD10	0.480692341	2.909153263	16.83402816	6.67E-05	0.003363653
SBDSP1	0.463845503	3.336662162	16.82470872	6.70E-05	0.003366676
SMIM15	0.372161917	4.087251186	16.81228398	6.74E-05	0.003366676
RPF2	0.451664411	2.852960348	16.81076637	6.74E-05	0.003366676
GNL3	0.580653299	4.250962339	16.78089958	6.84E-05	0.003394473
THAP12	0.359701204	5.69948271	16.76940358	6.87E-05	0.003394473
TMEM126A	0.650135377	1.834032583	16.76526409	6.89E-05	0.003394473
HINT1	0.677942255	5.251119471	16.73788936	6.98E-05	0.003427445
MRPL15	0.510033116	2.927005895	16.7214498	7.03E-05	0.003431605
SNHG12	0.344266613	4.040198306	16.70445869	7.09E-05	0.003448052
PSMA4	0.544455357	5.257304275	16.6584456	7.25E-05	0.003501358
OSTC	0.532451012	3.876201598	16.65166706	7.27E-05	0.003501358
ZNF260	0.576067054	4.022887431	16.65064587	7.27E-05	0.003501358
ATP5F1C	0.530121183	5.152643731	16.64471961	7.29E-05	0.003501358
SNORD15B	1.484068941	2.825148501	16.6307993	7.34E-05	0.003513222
VAMP8	0.469202473	4.9162478	16.6216912	7.37E-05	0.003517126
CASP3	0.376016832	4.794590707	16.59586394	7.46E-05	0.003549117
ATP5PO	0.540737133	3.697671128	16.5840157	7.51E-05	0.003557762
ANP32E	0.411554546	5.343634752	16.56673243	7.57E-05	0.00356727
GNL2	0.349377314	4.530896071	16.54929677	7.63E-05	0.003582649
DPY30	0.440132014	3.183696117	16.53847885	7.67E-05	0.003589772
MT-ND4	-0.64031296	11.37186145	16.49266446	7.84E-05	0.003640761
ZNF493	0.441180754	5.981224474	16.49004513	7.85E-05	0.003640761
LSM5	0.614065912	2.844697081	16.48628923	7.86E-05	0.003640761
C1GALT1C1	0.38315043	3.699777838	16.48245777	7.88E-05	0.003640761
RPLP0	0.54733583	8.622583797	16.43861755	8.04E-05	0.003694441
SBF2-AS1	0.703767286	1.865719386	16.40982777	8.15E-05	0.003733814
RPL27	1.036604404	7.133677371	16.39898992	8.20E-05	0.003741587
IER3IP1	0.530196954	2.462213857	16.38959729	8.23E-05	0.003746837
MDFIC	0.397491145	6.06272976	16.34878626	8.39E-05	0.003769267
PAIP1	0.390917512	4.479395927	16.3487266	8.39E-05	0.003769267
EVI2A	0.843221903	5.94773969	16.34029534	8.43E-05	0.003769267
TXN	0.759714891	3.481053728	16.33903328	8.43E-05	0.003769267
AC005912.1	1.288821205	6.665215335	16.33880825	8.43E-05	0.003769267
PHAX	0.37056796	4.302197667	16.2821888	8.66E-05	0.003859227
C4orf46	0.326802491	2.240222308	16.27664461	8.69E-05	0.003859227
RPL27A	0.585554409	8.6331405	16.26710681	8.73E-05	0.003865246
ZNF22	0.624057683	3.892214616	16.21886746	8.93E-05	0.003943296
ZBED5	0.428598367	6.165765055	16.177651	9.11E-05	0.004009561
FCRL2	1.761495745	4.759115147	16.15935898	9.19E-05	0.004032719
SFR1	0.532401438	1.943301648	16.12878976	9.32E-05	0.00407985
AC009093.2	-0.43195558	3.48510469	16.11205329	9.40E-05	0.00408847
NANOGP4	-0.7710002	1.142037771	16.07385794	9.57E-05	0.004127906

FCRL5	2.171663739	5.522915154	16.07380698	9.57E-05	0.004127906
TPT1	0.947541193	9.742268755	16.07359237	9.57E-05	0.004127906
SNHG32	0.457495227	2.978599569	16.04798306	9.69E-05	0.004166543
POLR3GL	0.473453212	2.819045521	16.02208069	9.81E-05	0.004206176
SCARNA5	1.260310808	5.863096852	15.99463398	9.94E-05	0.004249363
MT-ND2	-0.68359648	10.65565723	15.98784963	9.97E-05	0.004250925
ZNF181	0.64003261	3.606859835	15.9776041	0.000100178	0.004253172
RPL6P27	0.880596128	4.639300323	15.97476622	0.000100313	0.004253172
HSPA13	0.388751923	4.324614629	15.95603386	0.000101214	0.004279172
SLC2A14	-1.64510291	0.673066421	15.94404963	0.000101795	0.00428892
SACS	0.481001775	5.935030794	15.87936317	0.000104988	0.0044012
ZNF28	0.434945252	4.385869786	15.86989389	0.000105464	0.004408731
ANKRD49	0.540131043	4.495045557	15.84721521	0.000106612	0.004420261
NSA2	0.67329047	5.072728038	15.84044418	0.000106958	0.004420261
ATP5MPL	0.609287313	4.196804437	15.83631251	0.000107169	0.004420261
CEP20	0.323693797	3.965459252	15.83151469	0.000107415	0.004420261
SCOC	0.631890203	3.09585851	15.8294407	0.000107521	0.004420261
EGFL7	-0.94201436	1.134748941	15.81770036	0.000108126	0.004432883
BACH1-IT2	-0.59147091	3.053866408	15.80622396	0.000108721	0.004445016
MRPL3	0.49605253	4.676178644	15.77157592	0.000110537	0.004494964
MIR17HG	0.513860953	3.232310473	15.77138267	0.000110547	0.004494964
AC008894.2	-1.12042393	1.523352919	15.73326591	0.00011258	0.004545331
ESCO1	0.367037227	5.560729761	15.73053872	0.000112727	0.004545331
CSTA	1.04394191	4.23092559	15.71787601	0.000113412	0.004545331
KNL1	0.934163038	2.854718062	15.71649227	0.000113487	0.004545331
RAN	0.386559063	5.685969886	15.71407563	0.000113618	0.004545331
ZNF252P	0.404714683	4.822091629	15.66349832	0.000116401	0.004638103
ZNF708	0.533063221	5.346714827	15.66063645	0.00011656	0.004638103
CSNK1G3	0.452659225	5.165486878	15.62804728	0.000118393	0.004684614
RPL21P75	1.316299974	1.8504607	15.6185787	0.00011893	0.004684614
METAP2	0.441133931	4.627189527	15.61756323	0.000118988	0.004684614
GPATCH11	0.569617661	4.226421812	15.60586859	0.000119656	0.004688277
AC018638.4	-0.36401065	5.725216347	15.59864922	0.000120071	0.004690006
TAF9	0.466763412	3.55371047	15.5842022	0.000120904	0.004709474
IGHG1	-1.43250261	5.0785386	15.5790656	0.000121202	0.004709474
KRT18P31	-0.34073585	4.522501922	15.54954441	0.000122928	0.004749643
CENPE	0.712490401	2.236044582	15.548087	0.000123014	0.004749643
RPLP1	0.414393756	8.842419036	15.54504372	0.000123193	0.004749643
TNFRSF14-AS1	-0.34844742	4.656797991	15.53518776	0.000123776	0.00475478
NDUFAF8	0.423237692	3.196960897	15.50788393	0.000125406	0.004797679
MZT1	0.471891108	2.337390949	15.47864087	0.000127176	0.00485292
AC144831.1	-0.65670186	1.546528925	15.45341003	0.000128724	0.004886915
ZNF180	0.347558059	4.109522439	15.43037946	0.000130153	0.004921661
LPAR6	0.853855516	4.854232682	15.42802185	0.000130301	0.004921661
UBE2Q2	0.344013591	4.911346478	15.42206166	0.000130674	0.004923254
ZNF43	0.343679661	4.962731412	15.3656686	0.000134257	0.005045488
CCDC59	0.453551945	3.982614502	15.35423918	0.000134995	0.005060458
SMIM10L1	0.39434862	4.291367928	15.3254276	0.000136875	0.005112146
COMMD3	0.520997882	2.556847054	15.32259721	0.000137061	0.005112146
ZBTB32	1.636055455	1.921099082	15.28992685	0.000139228	0.005179982
MRPL58	0.413300585	2.846682361	15.27285347	0.000140374	0.005209604
LIG4	0.410009246	4.120056273	15.25476518	0.000141599	0.005229748
FIGNL1	0.494797353	3.384874594	15.2535968	0.000141679	0.005229748
PTRHD1	0.535932247	3.021079558	15.24930092	0.000141971	0.005229748

AC034236.1	1.167255662	2.657853232	15.23114417	0.000143215	0.005262539
TAF13	0.419603025	2.509299774	15.22308915	0.000143771	0.005269934
ZNF813	0.565598322	3.122461882	15.19876402	0.000145461	0.005318796
CMTR2	0.434918178	5.031229761	15.17905641	0.000146845	0.005351193
RARS1	0.33720899	4.925054876	15.17593147	0.000147066	0.005351193
BBS12	0.482484787	1.723906389	15.11931399	0.000151125	0.005485459
ZNF184	0.47405231	3.633625298	15.08925078	0.000153326	0.005551821
RPL36AL	0.586688254	5.33772304	15.05519135	0.00015586	0.005629858
ZNF761	0.533648203	3.474557541	15.03359553	0.000157488	0.00567381
EIF4EP2	0.534090345	1.16324261	15.02616687	0.000158052	0.00567381
IDI1	0.49042481	5.006315329	15.0189614	0.000158601	0.00567381
FRG1	0.408900083	3.689314719	15.00230163	0.000159879	0.005705786
RPS8	0.691673654	8.268662313	14.99384933	0.000160531	0.005715349
FKBP3	0.473893107	3.75243652	14.97240778	0.000162197	0.005760887
HSPA8	0.373193805	8.944613784	14.9431157	0.000164502	0.005828837
KLHL14	1.626747795	2.950033597	14.93709695	0.000164979	0.005831877
POLR1F	0.463508949	3.489404972	14.92766833	0.00016573	0.005844548
ZNF583	0.561051843	2.842384935	14.91732045	0.000166559	0.005847736
MALAT1	0.355999173	14.5325294	14.9167243	0.000166607	0.005847736
NDUFB2	0.44557582	4.185530211	14.89140204	0.000168652	0.005905609
SVIP	0.55364489	4.441448243	14.85365678	0.000171749	0.005999935
ZRANB3	0.39586717	2.350390285	14.76082052	0.000179615	0.006222834
RPL5	0.711786461	7.600876844	14.74016152	0.000181415	0.006264088
MTATP6P1	-0.60334825	7.341486895	14.72683629	0.000182586	0.006282728
SPAG7	0.382837319	4.190540931	14.71866021	0.000183308	0.006285693
ZC3H15	0.458100428	5.082222439	14.70214329	0.000184776	0.006312779
ZNF32	0.565508982	3.133421343	14.69595099	0.000185329	0.006312779
HIGD1A	0.418288201	3.248587792	14.69548796	0.000185371	0.006312779
COL7A1	-2.25567154	2.695524053	14.66643327	0.00018799	0.00638737
RPS3AP47	1.939428328	0.741328924	14.64281365	0.000190147	0.006445953
ZBTB6	0.339282183	4.369855692	14.60970508	0.000193214	0.006515153
H4C8	0.358096008	4.973007617	14.60662185	0.000193502	0.006515153
LINC01480	1.495977477	1.27537627	14.59266781	0.000194812	0.006544437
GDF7	2.057739912	2.67125856	14.57489972	0.000196492	0.006586025
PRR33	-0.61410984	1.398448959	14.5647823	0.000197456	0.006603447
CDC37L1	0.380441732	4.015283123	14.54525277	0.000199329	0.006641733
CXCR2P1	-0.95636064	4.751669347	14.54355316	0.000199493	0.006641733
ZNF432	0.439009017	3.642703825	14.50425089	0.000203322	0.006739049
CAPZA2	0.425514605	6.629014263	14.47919316	0.000205802	0.006806093
SMAD1	-0.49112585	1.982510212	14.42559745	0.000211211	0.006969478
ARHGAP5	0.453624427	5.747363966	14.40469146	0.000213359	0.007024811
CEACAM8	1.874871264	3.200194572	14.36977671	0.000216998	0.007128842
VRK1	0.707173393	4.471884514	14.35480603	0.000218578	0.007164916
THOC7	0.344781131	3.83661608	14.3476071	0.000219341	0.007174148
AL121753.2	-0.32349904	3.61240442	14.30721408	0.000223677	0.00728395
SRGN	0.683685554	9.245039464	14.28853689	0.000225712	0.007330886
BCAS2	0.370785009	3.416740948	14.27764664	0.000226907	0.007340919
TSBP1-AS1	1.681323394	1.326958009	14.26591961	0.0002282	0.007366765
UGCG	0.617591181	5.159388925	14.2391372	0.000231184	0.007446921
SNHG9	-0.61353906	1.919790315	14.22432879	0.00023285	0.007484406
MTND4P14	-0.4776928	3.047487831	14.2100862	0.000234465	0.007507079
ESF1	0.590457352	3.647309027	14.20920922	0.000234565	0.007507079
C12orf57	0.565846696	4.828563459	14.2005895	0.000235548	0.007522365
SERPINI1	0.582099382	1.94562878	14.15779908	0.000240491	0.007628787

KBTBD6	0.394371214	3.709846376	14.15361906	0.000240979	0.007628787
SBDS	0.406615394	4.051412719	14.15050494	0.000241344	0.007628787
TPTEP1	-1.16081183	3.117944448	14.14321164	0.0002422	0.007635158
HNRNPH1P1	-0.44296977	1.762334446	14.13537289	0.000243123	0.007635158
NPM1	0.55190635	6.714545157	14.13017774	0.000243737	0.007635158
DPM1	0.432575318	4.360205728	14.12034213	0.000244904	0.007640804
ZNF273	0.432942981	3.224110673	14.09411882	0.000248043	0.007706426
USP16	0.367486894	5.475058395	14.07909026	0.000249861	0.007730613
MTND6P11	-1.1399119	1.590082549	14.06780281	0.000251235	0.007756996
RSL1D1	0.385170517	5.935676955	14.05880646	0.000252335	0.007774847
ZNF84	0.450148402	5.131669575	14.05177919	0.000253198	0.007785322
AL355032.1	2.221921197	1.102215993	13.99386966	0.000260427	0.00797499
PSMD14	0.395176961	3.851691167	13.98111991	0.000262046	0.00797499
SMIM20	0.486360858	2.397485786	13.95401593	0.000265523	0.00805283
IGLV1-44	-1.1507426	2.022468256	13.95275541	0.000265686	0.00805283
BCL2A1	1.000112363	4.511517003	13.91497308	0.000270615	0.008167036
NUP88	0.417180178	5.59429187	13.89884802	0.000272747	0.008167036
ZNF615	0.384916511	3.925000466	13.88363413	0.000274774	0.008211214
DMAC1	0.523707786	3.75559924	13.84908605	0.000279434	0.008333753
SLC24A3	-0.87109586	2.330544308	13.83890478	0.000280823	0.008347306
KTN1	0.400437678	6.43194191	13.83753428	0.000281011	0.008347306
CETN3	0.653256957	1.757959491	13.80058534	0.000286114	0.008466423
RMI1	0.328347086	3.591724456	13.78055589	0.000288919	0.008497856
GAS5	0.672844475	4.787541593	13.7804518	0.000288934	0.008497856
HSP90AA2P	0.883039672	0.998704155	13.77484846	0.000289724	0.008504283
ADTRP	1.201316687	3.304416244	13.76253698	0.000291467	0.008534637
PCMT1	0.33135022	5.065071342	13.75864323	0.000292021	0.008534637
CWC15	0.389728309	4.011899454	13.7544008	0.000292625	0.008534637
PPIL1	0.382892726	2.574926697	13.7514119	0.000293052	0.008534637
WFIKKN1	-0.70019589	1.27888516	13.74019425	0.000294659	0.00855918
CEP57	0.407133183	5.441374936	13.73750653	0.000295045	0.00855918
AL158211.5	0.535181118	2.259584609	13.7067256	0.000299506	0.008671691
NIFK	0.47238595	3.938772986	13.69858364	0.000300698	0.008689284
BMI1	0.391384731	4.117200364	13.67489684	0.000304191	0.008773212
CEACAM6	1.762310148	1.533459004	13.64975149	0.000307946	0.008838185
COPS2	0.417340873	5.198828397	13.61608437	0.000313047	0.00895916
ZNF616	0.38300092	3.486498088	13.60853854	0.000314202	0.008974958
MIS12	0.350520051	3.845537019	13.59910991	0.000315651	0.008981879
ZNF593	0.516169621	1.668593484	13.5897131	0.000317103	0.009005956
NXT2	0.457646607	2.792360479	13.57983301	0.000318636	0.009032265
HMGB2	0.715494836	5.539167591	13.54634434	0.000323889	0.009149966
MRPS18C	0.410451439	3.779881981	13.54553348	0.000324018	0.009149966
SCARNA6	0.867490967	3.918736747	13.5085621	0.000329923	0.009280738
HYLS1	0.415742174	2.451495442	13.49783713	0.000331656	0.009280738
COQ5	0.383818623	3.071713905	13.49636798	0.000331895	0.009280738
RPL13AP25	0.852684231	4.548454987	13.49094259	0.000332776	0.009280738
NFU1	0.375657883	3.074229692	13.48876197	0.00033313	0.009280738
MYO15B	-0.49402237	7.425411382	13.48673571	0.000333461	0.009280738
PTCRA	-0.91962296	2.755552244	13.48564926	0.000333638	0.009280738
RPS18	0.905605502	8.111571246	13.39390627	0.000348944	0.009528418
RPL15P3	0.552098589	4.141519057	13.37129426	0.000352826	0.009581668
ZNF658	0.588133952	2.441503186	13.36085963	0.000354632	0.009613172
THAP9-AS1	0.374447055	4.245030627	13.35393809	0.000355835	0.009617388
RPL29	0.420991105	7.997858338	13.34859704	0.000356766	0.009618473

EGF	-1.03511265	1.732741823	13.30667795	0.000364163	0.009782435
EID1	0.430815085	5.723688537	13.28955538	0.000367229	0.009830905
PRXL2C	0.38270195	3.135172609	13.2892243	0.000367288	0.009830905
RPL10A	0.486840941	7.896890659	13.284621	0.000368117	0.009835402
INPP5F	0.860003804	4.552490603	13.24492565	0.000375345	0.009945779
ZNF888	0.389119579	3.500949118	13.24359294	0.00037559	0.009945779
MTND1P11	-0.4847541	3.293754797	13.23737328	0.000376736	0.009954722
NFYB	0.532543021	3.740342574	13.23299564	0.000377545	0.009954722
AIF1	0.63998801	6.037684955	13.23089267	0.000377934	0.009954722
IL10RB	-0.3517354	4.632951581	13.18571395	0.000386396	0.010142091
GATM	0.998617372	1.908169077	13.18100451	0.000387289	0.010147248
ZNF624	0.408107879	3.351152081	13.15542604	0.000392177	0.010239256
SLIRP	0.466463257	2.901778792	13.12906944	0.000397279	0.010336205
H4-16	0.371356937	5.169216322	13.0726039	0.00040844	0.010589549
SNRPB2	0.375500249	4.131351332	13.06561775	0.000409843	0.010607442
ZNF416	0.363706676	2.908644573	13.06137415	0.000410697	0.010611106
ZNF14	0.449208544	4.024589544	13.05559365	0.000411864	0.010622816
TCF4	1.133678435	5.626016209	13.04774775	0.000413454	0.01064536
RPL10P16	0.65064964	5.446891602	12.97815862	0.000427828	0.010902289
CASP8AP2	0.363425282	5.782837057	12.95104298	0.000433566	0.01101081
GIMAP7	0.61145745	6.737960283	12.93620676	0.000436739	0.011053663
ZNF836	0.390557438	4.090517704	12.92025681	0.000440177	0.011102901
RWDD1	0.478576959	4.107070481	12.88778334	0.000447261	0.011224522
MTERF3	0.364293895	2.86218932	12.8833604	0.000448235	0.011230025
ARV1	0.37633539	2.606638381	12.87003788	0.000451182	0.011234297
SRFBP1	0.422757289	3.429484525	12.86582554	0.000452117	0.011234297
MT-ND3	-0.49571742	9.026295726	12.86554395	0.00045218	0.011234297
C1orf198	-0.70693812	3.348775375	12.850674	0.0004555	0.01127864
CCDC82	0.339982786	5.832769937	12.84400629	0.000456996	0.01127864
FBXO22	0.350127768	5.11169055	12.82735089	0.000460757	0.011333856
SMC6	0.454721682	5.37967733	12.81802664	0.000462876	0.011367192
PPIAP22	0.932212012	1.844108552	12.78282885	0.000470965	0.011541051
RPS14	0.620073164	8.187313681	12.76845663	0.000474309	0.011571592
POLE4	0.393945424	3.37558444	12.74052699	0.000480878	0.011664089
RNF216-IT1	-0.42476948	1.452164924	12.73571338	0.00048202	0.011664089
BPI	1.306115197	3.189427642	12.72337593	0.000484958	0.01171614
CAP2P1	-0.38464079	2.326911738	12.70506605	0.000489352	0.011803144
KCNAB3	-0.46017978	2.452819956	12.67335439	0.00049706	0.011931034
BAIAP2-DT	-0.46023649	4.440720434	12.66244913	0.000499739	0.011932478
SPIN4	0.438656654	2.600395948	12.66037796	0.000500249	0.011932478
RPL21P28	1.263330319	2.080793489	12.65523373	0.000501519	0.011932478
LIMD1-AS1	0.362667372	2.278348417	12.65437385	0.000501732	0.011932478
BACH1-IT1	-0.47568416	3.114550164	12.65357105	0.000501931	0.011932478
ZNF226	0.394078017	5.264908432	12.6375752	0.000505905	0.012007773
BIRC3	0.620519982	7.245266214	12.63093809	0.000507563	0.012027951
WNT10A	0.915395985	2.149555377	12.62648625	0.000508679	0.012035221
MIR6124	-0.70030355	1.538371375	12.62281315	0.000509601	0.012037902
UBL5	0.437908714	4.984266142	12.61778756	0.000510865	0.01204865
BCL2	0.550703009	7.146117791	12.61332347	0.000511991	0.012056099
GEMIN6	0.434763394	2.468469841	12.60720436	0.000513539	0.012073436
TBCA	0.428542017	4.000641449	12.5639279	0.000524621	0.012295132
ATP5F1E	0.615565872	6.318026862	12.538436	0.000531263	0.012390146
RPL22	0.454718196	5.868934061	12.52375714	0.000535126	0.012390956
DAB2	-0.59699116	4.364159837	12.52080782	0.000535906	0.012390956

ZNF138	0.472694235	3.779888492	12.51968569	0.000536203	0.012390956
AC087893.2	-0.40931137	1.812766255	12.50308918	0.000540615	0.01244937
PTGER2	0.394603471	5.102105744	12.50074738	0.000541241	0.01244937
CTSG	1.743592703	0.901174976	12.46411311	0.000551123	0.012618166
MAIP1	0.328558898	2.516657356	12.45096791	0.000554713	0.012670219
RPS27AP12	0.464665762	1.434852258	12.44956167	0.000555099	0.012670219
SNHG8	0.707632202	3.390912678	12.44494725	0.000556366	0.012679692
PIN4	0.399779044	2.593992159	12.41821192	0.000563765	0.012801403
ADH5	0.375161921	4.836874223	12.41634052	0.000564287	0.012801403
SPDL1	0.420266516	2.593435891	12.4088906	0.000566369	0.012829072
MAGOH	0.338032217	3.20632723	12.39130039	0.000571315	0.012921447
SNHG19	0.603242474	1.193555202	12.36259678	0.000579482	0.01308626
AC122718.2	-0.47220638	2.782631772	12.34758552	0.0005838	0.013163803
ZBTB1	0.344235159	6.351534121	12.32864237	0.000589296	0.013267635
RPS15A	0.934674614	6.717370465	12.27164819	0.000606154	0.01357336
PLEKHF2	0.412167845	5.241126077	12.2612142	0.000609293	0.013603846
HDGFL3	0.79297022	2.864220674	12.25426277	0.000611394	0.013603846
FBL	0.395479302	5.546431782	12.25410523	0.000611442	0.013603846
ATAD5	0.353012094	3.187378242	12.24354003	0.000614649	0.013632197
NOC3L	0.50593824	4.081346289	12.22764742	0.000619506	0.013691843
ZNF570	0.351773646	3.390639315	12.22337058	0.000620819	0.013691843
ARHGAP32	-0.44012487	3.766489211	12.13373471	0.000649017	0.014174503
EEF1E1	0.523302243	1.605220413	12.13199358	0.000649577	0.014174503
SNX2	0.356313677	6.329182169	12.10677678	0.000657751	0.014331844
MRPL32	0.511951372	3.284355988	12.08835168	0.000663789	0.014441751
ABLIM3	-1.08837791	2.885013777	12.08547956	0.000664736	0.014441751
MTCO1P12	-1.35584161	3.078891322	12.07521064	0.000668131	0.014494349
LSM7	0.375060114	4.356141165	12.07171286	0.000669291	0.014498389
AGPAT5	0.582843876	4.390939041	12.05676059	0.000674275	0.014573949
ZNF737	0.515170567	5.563147276	12.05537795	0.000674738	0.014573949
ZNF253	0.456341013	4.150617931	12.05113409	0.00067616	0.014583508
LSM1	0.365655353	3.430393684	12.04179323	0.000679302	0.014630066
COX7A2	0.491104481	4.394235632	12.01628212	0.000687959	0.014795093
AC005730.3	-0.41254608	1.625670976	12.00519648	0.000691756	0.014833873
MT-CYB	-0.55257601	10.61729112	11.99271565	0.000696056	0.014904611
SKA2	0.399617756	3.296891215	11.98895525	0.000697357	0.014911015
ZFP30	0.466530947	3.564247735	11.97656083	0.000701663	0.014960093
ZNF569	0.405042935	3.146767171	11.96408495	0.000706024	0.01503155
MTND6P5	-0.47260509	2.163611286	11.95383726	0.000709628	0.015086682
ATP5MD	0.463998769	3.364828157	11.88932507	0.000732747	0.015533828
SLC30A4	0.457011877	2.945804737	11.88215972	0.000735362	0.01556279
AP003117.1	0.697162017	1.219770015	11.87700153	0.000737251	0.01556279
ZWILCH	0.330590259	2.675715916	11.85773838	0.000744347	0.015668135
CHCHD1	0.393846654	3.110931873	11.8504118	0.000747064	0.015703121
TCF7L2	-0.49733871	5.45201654	11.82862974	0.000755203	0.015831214
MTATP6P11	-0.48303611	2.56782691	11.82840899	0.000755286	0.015831214
RECQL	0.39049988	5.306101152	11.81474245	0.000760439	0.015894453
BTLA	0.786989804	5.162636393	11.8108452	0.000761915	0.01590297
DDX50	0.353334002	4.714894813	11.80740482	0.00076322	0.015907908
RPL39P3	1.353897476	2.142135745	11.79850064	0.00076661	0.015956206
HSP90B2P	0.526314371	1.248925872	11.78727841	0.000770903	0.015978532
MTERF1	0.331811394	4.184404907	11.78033641	0.000773572	0.015989335
MRPL11	0.384188967	3.245150505	11.78033211	0.000773574	0.015989335
RPL15	0.414296261	8.717600548	11.77366381	0.000776146	0.016014425

CTBP2P8	-0.92442423	1.975880755	11.7716098	0.00077694	0.016014425
LSM8	0.365989683	6.153417777	11.74305154	0.000788066	0.016198891
AC009086.2	0.526706876	1.266586666	11.73778093	0.000790137	0.016212582
AC064805.1	-0.8930249	3.187719292	11.73384859	0.000791686	0.016212582
MTCO2P11	-0.46911405	2.443068749	11.72751869	0.000794186	0.016234991
NGDN	0.375734708	3.73237044	11.71598724	0.000798761	0.016285201
ZNF91	0.435153236	6.55262109	11.71127372	0.000800638	0.016285201
ZNF572	0.590051681	1.546078515	11.71031914	0.000801019	0.016285201
TMEM123	0.346815695	7.996496601	11.69981161	0.000805223	0.01632757
ZNF780B	0.37590387	5.716210755	11.68080556	0.000812885	0.016448619
CBX3P2	0.41373367	2.009765399	11.6786227	0.00081377	0.016448619
ETAA1	0.486280728	3.950475395	11.67268254	0.000816183	0.016448619
THAP2	0.393222444	3.031873387	11.67118002	0.000816794	0.016448619
POU5F2	-0.4111947	4.121237783	11.65657208	0.000822763	0.016546429
DNAJC15	0.583730895	4.252531344	11.64927419	0.000825762	0.016584323
DNM3	-0.63893027	3.68410222	11.63353917	0.000832265	0.016692409
AC243919.1	1.589278496	5.607829269	11.61408921	0.000840376	0.016809786
UQCRQ	0.495522316	3.692456479	11.59084136	0.000850178	0.016953139
ID3	0.908195382	3.635959164	11.59048263	0.00085033	0.016953139
AP000560.1	-0.99201219	1.233649319	11.58899663	0.000850961	0.016953139
JAM3	-0.75369738	3.559370031	11.58059099	0.000854536	0.01700162
IFI27	-1.54260385	2.001398096	11.56269075	0.000862203	0.017086014
EIF4BP7	0.357231918	2.337947251	11.53797724	0.000872903	0.017251724
MCOLN2	0.780750429	3.755012578	11.52189838	0.000879938	0.017336249
MTND5P2	-0.4258585	2.404861081	11.5020147	0.000888718	0.017448411
TRIM59	0.421518237	3.398524854	11.49854347	0.00089026	0.017455655
POMGNT2	-0.36910625	2.717691768	11.48181206	0.00089773	0.017532419
CD200	1.307724531	2.932146672	11.47980618	0.00089863	0.017532419
FCRL3	0.846153815	6.176993361	11.46934373	0.000903339	0.017596183
PAQR4	-0.45940585	2.811394768	11.44515624	0.000914321	0.017763616
ZNF614	0.359309085	3.650473619	11.43871623	0.000917268	0.017793578
IKZF2	-0.47305192	5.806633571	11.43320576	0.000919798	0.017800304
AL050341.2	0.500542573	1.742481273	11.41086826	0.000930125	0.017924981
SFMBT1	0.506742169	4.406015848	11.39861886	0.000935838	0.017946413
FAM200A	0.324197259	2.943689331	11.38052617	0.000944343	0.018063901
ASF1A	0.3335375	4.336484032	11.34869659	0.000959498	0.01828328
ZNF510	0.372147274	4.741245616	11.34536529	0.000961098	0.018290357
XRCC4	0.457910383	2.828459556	11.33680415	0.000965223	0.018322006
AC108134.3	-0.6668143	4.591964118	11.33025778	0.00096839	0.018358701
LINC00494	1.218892224	2.822939496	11.31029996	0.00097811	0.018483333
FBXO5	0.371839088	2.553134021	11.30911575	0.00097869	0.018483333
RBIS	0.523923529	3.404792669	11.29557216	0.000985347	0.018582749
SLC9A7	0.827500948	4.910322641	11.29315259	0.000986541	0.018582749
AC090114.3	-0.32624069	4.323829934	11.27676406	0.000994669	0.018666663
RPS12	0.526728761	8.49336645	11.27299622	0.000996547	0.018678357
GFI1B	-0.81675351	4.439343902	11.2607486	0.001002678	0.018746043
COL13A1	-1.0144127	1.872737414	11.25309441	0.001006529	0.018794428
RNU7-181P	-0.45928139	2.596730229	11.24331611	0.00101147	0.018839423
TUBB1	-0.87995062	7.74697584	11.22849942	0.001019005	0.018904141
SHLD3	0.490176709	1.630999573	11.22706259	0.001019739	0.018904141
H4C5	0.441895164	6.473555989	11.22650127	0.001020026	0.018904141
AC010359.3	-0.42732098	3.243426632	11.2118957	0.001027518	0.0190193
FCRLA	1.1155663	3.794036217	11.20424763	0.001031463	0.019068612
MRPS21	0.395432756	3.579054207	11.19779863	0.001034802	0.019106603

GEMIN2	0.459638505	1.790906019	11.18927844	0.00103923	0.019149322
ABCE1	0.380928655	5.116118188	11.18839758	0.001039689	0.019149322
RASGRP3	0.823239087	4.523091916	11.17668559	0.001045811	0.019231516
CCDC26	-0.73184085	1.191746147	11.17492077	0.001046736	0.019231516
CCDC167	0.508103198	2.41903229	11.15245951	0.00105859	0.019366245
RPF1	0.364420694	4.367643288	11.14954812	0.001060137	0.019366245
PAK1IP1	0.420601968	2.669713049	11.14953265	0.001060145	0.019366245
C20orf197	-0.4452948	3.551943584	11.14786473	0.001061032	0.019366245
CCNB1IP1	0.381706007	3.116158153	11.12335091	0.001074157	0.019550992
RPL23A	0.335108331	8.573349399	11.11125982	0.001080692	0.019613244
FAM162A	0.350239781	3.233377357	11.10484523	0.001084176	0.019633356
NDUFB1	0.376262357	3.802909581	11.1043612	0.001084439	0.019633356
RPS3	0.427360159	8.806665694	11.09982063	0.001086912	0.019641005
SENCR	-0.49268066	1.914223919	11.09874143	0.001087501	0.019641005
ZNF571	0.462669317	2.817743771	11.09026405	0.001092137	0.019648266
FAU	0.461914283	7.270940606	11.08865412	0.001093019	0.019648266
BEND4	1.136137376	2.461453122	11.08835384	0.001093184	0.019648266
TRMT12	0.352358522	3.525832319	11.05154014	0.001113571	0.019870703
DIPK1A	0.459023886	3.597923173	11.04776944	0.001115681	0.019884512
RPL3P2	0.615362038	0.870293054	11.03914969	0.00112052	0.019946867
SNHG5	0.43266677	5.601272983	11.01718839	0.001132946	0.020128225
CD48	0.375164953	7.306114492	11.01130668	0.001136298	0.020128225
ZNF302	0.424888793	4.861170612	11.01125492	0.001136327	0.020128225
SNORA12	0.945324062	2.778052554	11.00409199	0.001140423	0.020156506
RPS15AP29	-0.49218514	2.503309345	10.99990895	0.001142822	0.020174944
NFIA	-0.35519868	5.347024185	10.98981603	0.001148631	0.020253474
STAP1	0.960879838	3.167119632	10.9635157	0.001163912	0.020450221
AC064799.1	1.948316473	1.628718752	10.94179543	0.001176688	0.020626002
GUCY1A1	-0.60508974	2.937918729	10.92404581	0.001187236	0.020761975
RPL32	0.51440109	8.046593947	10.91877193	0.001190388	0.020792672
ACADM	0.442761227	4.439071892	10.9047037	0.00119884	0.020915746
OXSM	0.374682333	2.344616514	10.89283799	0.001206016	0.021005315
UBAC2-AS1	0.482524358	1.286559061	10.86932403	0.001220367	0.02116657
NDUFAF4	0.537388986	2.405893725	10.86455679	0.001223297	0.02116657
TCEAL8	0.485881809	2.91554928	10.8542623	0.001229651	0.021204707
KLF8	0.746673688	3.438273025	10.84039717	0.001238261	0.021320247
RPS29	0.883300114	7.864695156	10.83886572	0.001239216	0.021320247
MAP3K9	0.535448917	3.587284602	10.8165843	0.001253194	0.021486212
SERPINE1	-0.89711899	1.018730847	10.81056049	0.001257001	0.021526676
EXT1	-0.33914745	4.488200654	10.79950848	0.001264015	0.021572336
ZNF675	0.386901295	4.387266954	10.79596802	0.001266271	0.021586077
ZNF566	0.388355301	3.289510329	10.77942599	0.001276865	0.021741764
AC015911.4	0.625147885	2.008559604	10.75916998	0.001289961	0.02188962
TXNDC9	0.389294257	3.683538377	10.75666627	0.001291589	0.021892288
ZNF484	0.351469362	4.930576667	10.73298485	0.001307094	0.022054624
CD3D	0.568951772	5.514711972	10.72517022	0.001312253	0.022116585
PBDC1	0.345288323	3.098045945	10.71230967	0.001320787	0.02219641
MT-CO2	-0.44251904	10.57929946	10.6951246	0.00133228	0.022326535
CCDC106	0.724903882	2.132125074	10.692996	0.001333711	0.022326535
CLEC17A	1.155230018	3.589804683	10.68525391	0.001338928	0.022382664
KIAA1586	0.441715562	4.016062293	10.67666609	0.00134474	0.022410318
MIER3	0.338082677	4.336696677	10.67185992	0.001348003	0.022415909
CISD1	0.405938391	2.596914565	10.66855613	0.001350251	0.022415909
ZNF441	0.515589132	3.938916297	10.66374975	0.001353529	0.022415909

MSRA	-0.3546813	4.460749574	10.64948101	0.001363307	0.022518013
AC139720.1	0.739654445	1.734491784	10.64352624	0.001367409	0.022552227
LYRM2	0.39687194	4.286232333	10.64207706	0.001368409	0.022552227
ZNF90	0.55953884	2.402466764	10.63459797	0.001373583	0.022612453
AF131215.4	-0.43401586	2.543524711	10.62611215	0.001379477	0.022634377
RPL37	0.549681611	7.843532399	10.58085918	0.001411352	0.023055699
AP1S3	0.804846947	1.372034757	10.57774831	0.001413571	0.023066624
ZNF790	0.378594217	3.059106297	10.56591918	0.00142204	0.023128747
CXCR4	0.340312651	7.844226742	10.56278063	0.001424296	0.023140148
EFCAB13	0.673176048	3.160954448	10.5596555	0.001426546	0.023151426
FAM3C2P	0.813789118	0.898770828	10.53152362	0.001446964	0.023353215
RPL3P4	0.515486372	6.293496732	10.52605584	0.001450967	0.023353215
CTIF	-0.38306707	4.031786623	10.52528273	0.001451534	0.023353215
SNORD3C	2.282514001	0.825216813	10.51713021	0.001457526	0.023414649
ACTBP4	-0.52803994	1.828172195	10.50075565	0.001469638	0.023568048
RPS27AP16	0.82261403	3.249836779	10.49017308	0.00147752	0.023598491
PDGFA	-0.84355503	2.412024301	10.48995213	0.001477685	0.023598491
PTGS1	-0.7266352	5.913948207	10.47843794	0.001486312	0.023672768
ZDHHC21	0.390400819	5.004789066	10.47712018	0.001487303	0.023672768
AP000763.3	-0.40710282	4.138069668	10.47237145	0.001490878	0.023704323
DPH5	0.454623456	3.488457108	10.44982719	0.001507972	0.023950526
LRRCC1	0.428789777	2.150234634	10.44710185	0.001510052	0.023957994
ABCA13	1.365077142	3.209974603	10.44110404	0.00151464	0.024005195
COCH	1.694821784	1.622685641	10.43278914	0.001521025	0.024059098
CYB561A3	0.435768369	6.043569868	10.43036448	0.001522891	0.024059098
COA6	0.444666553	2.358716439	10.36669977	0.001572759	0.024682819
MRPL1	0.496770449	2.694979488	10.35946586	0.001578531	0.024682819
DNAJC10	0.366233286	6.185123816	10.35893191	0.001578957	0.024682819
SNRPD1	0.395996003	3.217108893	10.35223074	0.001584324	0.024718238
C10orf88B	0.489357503	1.43739199	10.35146926	0.001584935	0.024718238
AC022167.2	-0.37643571	3.673668239	10.3379386	0.001595834	0.024842256
MTCO1P11	-0.42275123	3.538408907	10.33381208	0.001599173	0.024852112
PIGW	0.344412106	1.908160912	10.33302942	0.001599807	0.024852112
GZMA	0.738505148	4.750455079	10.32627538	0.001605289	0.024911279
LINC01146	-0.50148347	3.425246567	10.32316771	0.001607819	0.024924538
AL158163.2	-0.36607781	1.68850231	10.31112851	0.001617655	0.025019332
TSC22D1	-0.33336008	5.690622449	10.30951648	0.001618977	0.025019332
YEATS4	0.501496829	2.686726487	10.29700961	0.00162927	0.025126211
AL356273.3	-0.36650722	2.929969544	10.29275812	0.001632784	0.025154338
TAX1BP1	0.325975498	6.894350801	10.2899068	0.001635145	0.025164663
OSGEPL1	0.343164154	2.738031296	10.2875892	0.001637067	0.025168211
MITF	-0.47393076	2.079901885	10.28207753	0.001641646	0.025212571
HSP90AB4P	-0.32567731	2.759646316	10.27697665	0.001645896	0.025244393
AC020658.5	-0.5830202	1.282666135	10.27465859	0.001647831	0.025244393
RPL8	0.357300903	9.05482533	10.27071579	0.001651128	0.025244393
AL162274.2	-0.37990409	2.119271152	10.2538263	0.001665327	0.025392825
SMOX	-1.08993049	4.941337312	10.23855041	0.001678277	0.025537956
EEPD1	-0.50273181	5.643930024	10.20301717	0.001708801	0.025922919
ACAT2	0.327005527	3.172553845	10.19626721	0.001714664	0.025925817
GUCY1B1	-0.70115687	2.686064369	10.19605083	0.001714852	0.025925817
FNBP1L	-0.40743922	2.764875514	10.19477722	0.001715961	0.025925817
DUSP19	0.496922166	1.194938698	10.18998948	0.001720135	0.025962522
ZNF267	0.38740352	6.323795396	10.1722031	0.001735733	0.026082817
AC008964.1	-0.32375555	3.364974954	10.17184803	0.001736045	0.026082817

ASNSD1	0.427850562	3.551507947	10.17091462	0.001736868	0.026082817
TMEM184B	-0.35893913	6.331581172	10.16563071	0.001741533	0.026126504
EEF1A1P6	0.725888357	3.146506067	10.15063532	0.001754841	0.026273186
TREML1	-0.94249806	3.990567038	10.1352474	0.001768606	0.026399598
HMGN3	0.380741453	3.826460856	10.12442585	0.001778353	0.026491941
KCNN4	0.882806904	4.446788792	10.12005073	0.001782309	0.026497826
ZNF850	0.329784578	2.566251794	10.11108595	0.001790444	0.026592198
SLC44A5	2.453958878	0.597734295	10.09615578	0.001804076	0.026767951
AC018926.3	-0.49773488	1.897825487	10.08562078	0.001813759	0.026868118
TIMM17A	0.32462751	3.464519047	10.08488451	0.001814437	0.026868118
ZNF644	0.324657396	6.254847578	10.04889287	0.001847935	0.027255674
NRGN	-0.84925069	7.542308052	10.03383696	0.001862136	0.027371364
CCDC25	0.330067138	3.822381651	10.01306862	0.001881908	0.027511349
GCSAML	-1.06811389	1.484865545	9.999075924	0.001895351	0.027626454
IFNLR1	0.749098715	3.09022578	9.992474465	0.001901727	0.0276652
AGGF1P2	0.922999868	0.922618503	9.925836107	0.001967337	0.028425156
PTPRN2-AS1	-0.62970788	3.864965272	9.916683199	0.001976528	0.028502613
NDUFS4	0.472477386	2.748737994	9.908481767	0.001984802	0.028573708
CHML	0.463629122	4.889790656	9.907990402	0.001985299	0.028573708
LTV1	0.379414213	2.87745938	9.90317735	0.001990172	0.028588547
TMEM144	-0.61232621	2.956949734	9.895750102	0.001997716	0.02866098
F13A1	-0.71970164	7.362325174	9.894423807	0.001999066	0.02866098
ZNF527	0.384567667	3.132376173	9.892185221	0.002001347	0.028666068
ZNF879	0.441020977	3.141769553	9.889255871	0.002004336	0.028681276
AC092652.2	-0.34925893	3.370540371	9.880069079	0.002013739	0.028788156
AC009093.8	-0.41354616	1.47594452	9.862753281	0.002031587	0.028987613
PRNCR1	-0.52886368	2.583059998	9.847163913	0.002047794	0.029162942
MT-ND5	-0.57355116	10.733952	9.833155447	0.002062471	0.029232089
FGFRL1	-0.34021025	3.819299052	9.807494112	0.002089637	0.029560817
ACRBP	-0.53458177	4.254688062	9.803100062	0.002094326	0.029599005
WDR5B	0.330572934	3.647161828	9.781770071	0.002117238	0.029837823
EIF2A	0.328243932	5.448672942	9.779103547	0.002120121	0.029850177
LINC01237	0.467661671	2.377074388	9.776078433	0.002123396	0.029868029
RABAC1	-0.36652694	3.921480099	9.76992468	0.002130074	0.02993367
SETD9	0.59942584	1.392462877	9.765434421	0.00213496	0.029974033
RPL14	0.358673704	7.956611074	9.763556491	0.002137007	0.029974495
ADAMTS6	0.860202104	1.681791593	9.741009503	0.002161742	0.030149708
CHMP2A	-0.40897497	6.672914173	9.738824952	0.002164154	0.030149708
CLIC1	0.332186887	7.517719867	9.727223606	0.00217701	0.030149708
SMTN	-0.38839265	2.243758764	9.721567631	0.002183306	0.030168954
ZNF141	0.385152977	5.76126312	9.711073056	0.002195038	0.03023199
RBBP8	0.369518449	3.218911548	9.698984685	0.002208632	0.030321828
ARHGEF17	-1.16204465	1.435958307	9.692969805	0.002215428	0.030362861
SMG1P3	0.358105595	5.937748557	9.690066472	0.002218716	0.030362861
TMEM126B	0.325416695	3.530049567	9.689126566	0.002219782	0.030362861
SHLD2	0.379038581	4.472714016	9.66909252	0.002242619	0.030479141
SGCB	0.428249249	2.215072042	9.641704686	0.00227423	0.030828551
RPS16	0.403038233	8.442585375	9.641435799	0.002274543	0.030828551
RNU4-2	0.932632635	5.85020017	9.638867612	0.002277531	0.030840961
PPM1H	-0.4748932	2.870481518	9.636168825	0.002280675	0.030855463
MYL9	-1.10513382	4.312770483	9.634101534	0.002283087	0.030860035
IL7	0.816619604	1.591432527	9.627041854	0.002291342	0.030915459
AC138035.1	-0.61746909	2.422423701	9.625227023	0.002293469	0.030916129
AL078622.1	1.183831157	1.172769487	9.596403026	0.002327526	0.031261946

SH3BGRL3	-0.38410513	8.499341055	9.57381099	0.002354582	0.031596829
SOD1	0.352575302	5.178059541	9.562906093	0.002367757	0.03169982
SDK2	1.24410232	4.468947823	9.551456129	0.002381672	0.031845495
ZFAND1	0.363111161	3.722441839	9.544482173	0.002390188	0.031902043
BAIAP2	-0.47738449	3.971134833	9.541639759	0.002393668	0.031919864
C8orf37	0.50137206	1.283986703	9.535603283	0.002401076	0.031969241
ZNF17	0.323524619	3.619449457	9.531384747	0.002406267	0.031969241
CD19	0.942552783	3.497854819	9.51456275	0.002427081	0.032156393
UGT8	1.031314414	1.580516665	9.513267826	0.002428691	0.032156393
ABAT	-0.37843314	5.285926076	9.485979901	0.002462873	0.032532724
AC040904.1	-0.67319406	1.440713891	9.47675997	0.002474534	0.032573981
CCDC18	0.361937793	4.177857084	9.476584143	0.002474757	0.032573981
SGO2	0.507360482	2.08276614	9.475956568	0.002475552	0.032573981
ZFP14	0.33853818	4.460437081	9.460426766	0.00249533	0.032724421
MIR4432HG	0.729923536	2.369138884	9.457721082	0.002498792	0.032724421
CCDC65	0.527998036	2.161911634	9.457536218	0.002499029	0.032724421
PARD3	-0.76411552	1.393278247	9.454922889	0.002502378	0.032724421
GMFG	0.40526151	6.905950367	9.447937901	0.002511352	0.032812946
NRXN2	-0.73165283	1.028378298	9.434827102	0.002528286	0.032976301
SDK1	-0.77804506	1.589109194	9.429596019	0.002535076	0.033004497
AC006364.1	1.253756414	0.982154893	9.428037903	0.002537102	0.033004497
MTCYBP3	-0.40855546	2.646363518	9.42288699	0.002543811	0.033049708
AP003068.2	-0.67708144	3.666629582	9.420782995	0.002546556	0.033049708
RN7SL288P	-0.42950084	3.050758871	9.420259875	0.002547239	0.033049708
ZNF680	0.391237317	3.69691352	9.415661482	0.002553252	0.033098869
PMAIP1	0.531760406	3.443885603	9.392055088	0.002584351	0.033414698
FTH1	-0.41968861	10.64818486	9.379396351	0.002601188	0.033603195
RTL5	-0.53856038	1.794401313	9.357631565	0.0026304	0.033921675
NUDT15	0.39889684	1.722741949	9.338020898	0.002657009	0.034159486
ZFP82	0.50610031	3.309916964	9.335597048	0.002660317	0.034159486
AC005041.3	-1.14290909	0.607648395	9.322507592	0.002678255	0.034301025
AC003681.1	-0.58221281	1.560360502	9.280725718	0.002736348	0.03486501
RCN2	0.349423696	4.307114529	9.271448834	0.002749421	0.034914328
COL6A3	-0.50027783	2.367187151	9.267791524	0.002754593	0.034914328
AC010201.2	-0.46384744	1.570174207	9.265673007	0.002757593	0.034914328
KPNA5	0.371712896	4.871112748	9.264689326	0.002758987	0.034914328
TMEM19	0.41379586	4.049618654	9.238973977	0.002795693	0.035258907
DEFA4	1.51059908	2.114306762	9.208277096	0.002840168	0.035691303
AQP10	-1.11110649	1.137293448	9.202127689	0.002849165	0.035691303
AC022167.1	-0.41108036	1.855489226	9.199000428	0.002853751	0.03571869
BNIPL	-0.36310916	1.588601974	9.192494253	0.002863317	0.035733212
TRAPPC2L	0.325766182	3.902830138	9.181778549	0.002879145	0.035825611
KIF15	0.715529524	1.756419307	9.166805299	0.002901411	0.036012348
LINC02631	-0.50990401	2.587533076	9.164441555	0.002904943	0.036026131
PPP1R12B	-0.47882452	7.529063045	9.160647535	0.00291062	0.036036665
TNFSF4	-0.51578999	2.773268209	9.156497875	0.002916842	0.03604779
LRRC40	0.327904108	3.564351167	9.155194052	0.0029188	0.03604779
FUNDC1	0.336133202	2.070405733	9.134472606	0.002950099	0.036227698
SH3BGRL2	-0.8212136	4.987186583	9.133272579	0.002951922	0.036227698
NME1	0.496604747	1.139500391	9.123129026	0.002967379	0.036358438
RPS27AP5	0.989158414	0.766334869	9.121411876	0.002970004	0.036358438
SLC38A5	-1.03766724	6.321038764	9.087153923	0.003022872	0.036874088
TPM3P7	-0.37234916	1.350214837	9.083329864	0.003028834	0.036916545
CRIP1	0.342688506	3.657119062	9.074402776	0.003042797	0.037056385

ARHGAP26-IT1	-0.51150933	3.076597513	9.066239021	0.003055624	0.037171395
AL391832.2	-0.64777016	3.753217839	9.063630792	0.003059734	0.037171395
BX571818.1	0.838327905	2.631126347	9.05377108	0.003075321	0.037253956
GPR18	0.528658305	3.278624195	9.034275423	0.003106384	0.037523702
CCR3	-0.88078477	5.251557886	9.003133302	0.003156673	0.037913626
AC122718.1	-0.38883232	2.85259243	9.003039032	0.003156826	0.037913626
IGLC2	-0.95685544	5.458156866	9.001064112	0.003160044	0.037913626
AL844908.1	-0.42817428	2.237019279	8.996655125	0.003167239	0.037925067
VIL1	-0.9187021	1.332792566	8.988215913	0.003181058	0.038024498
GLO1	0.334507634	4.175878997	8.97284888	0.00320638	0.038265717
AC006017.1	-0.35878271	2.348214015	8.951369118	0.003242123	0.038599423
RPL19	0.348706291	9.100051737	8.943279756	0.00325569	0.038729962
RPS4X	0.351946358	8.776758125	8.937555095	0.003265327	0.038782594
AL669831.1	-0.41536768	4.404981913	8.906867182	0.003317486	0.039213636
FAM3C	0.612540908	4.417744378	8.906282731	0.003318488	0.039213636
ALOX12	-0.95965783	3.075778551	8.905363812	0.003320063	0.039213636
SGPP1	0.420198298	4.924474758	8.866207805	0.003387917	0.039825417
ITGA1	-0.81555585	2.905163205	8.859209727	0.003400193	0.039850346
AC024293.1	0.69604129	4.162971138	8.858916031	0.003400709	0.039850346
SGSM1	0.684965624	3.077096621	8.85888855	0.003400758	0.039850346
KRT8P46	-0.3429292	3.760797019	8.856641898	0.003404709	0.039850346
TCF19	0.361740739	2.904571889	8.855849619	0.003406104	0.039850346
E2F5	0.884275526	2.909774792	8.848358532	0.00341932	0.039910835
CMTM5	-0.87348531	2.241501949	8.837617635	0.003438361	0.040038875
METTL8	0.530845602	4.346163529	8.830253389	0.003451479	0.040132724
SAMD14	-0.98845832	1.298782948	8.830065816	0.003451813	0.040132724
ZNF567	0.367900145	3.874451545	8.822873063	0.003464677	0.040198882
CLEC18A	-0.97791967	1.3259681	8.822353996	0.003465607	0.040198882
MT-ND6	-0.546594	5.182441748	8.817439286	0.003474427	0.04024626
PPP1R14BP3	-0.60951247	2.533851186	8.803094777	0.003500303	0.040475193
NIPSNAP3A	0.447176595	3.053944773	8.782379839	0.003538021	0.040721447
PAPSS1	-0.40703232	5.200657165	8.765297878	0.003569439	0.041051301
FHL2	-0.78925128	1.37320445	8.757932123	0.003583074	0.041112806
RALGPS2	0.689807758	6.236631909	8.750349011	0.003597168	0.041182576
MPIG6B	-0.99739238	5.338720614	8.750344284	0.003597177	0.041182576
TMEM256	0.619441339	1.124961596	8.731859136	0.003631773	0.041447891
GSK3A	-0.34416877	5.853227139	8.728551685	0.003637999	0.041487132
H1-3	0.401939463	5.83503489	8.723927787	0.003646722	0.041522968
ZMAT2	0.499538773	5.483092681	8.715669752	0.003662354	0.041615145
MRPL13	0.401753335	2.78972788	8.715220425	0.003663207	0.041615145
MFAP3L	-0.70636094	4.203036597	8.713175781	0.003667089	0.041627469
GP9	-0.83023056	4.085798092	8.702493199	0.003687439	0.041648325
YAE1	0.405229803	2.53221327	8.702000605	0.003688381	0.041648325
AC008462.1	0.423901256	1.463770456	8.701789212	0.003688785	0.041648325
YBX3P1	-0.97429472	2.612891424	8.700774378	0.003690725	0.041648325
APIP	0.367286109	3.506306755	8.68648279	0.003718158	0.041884978
ADAM28	0.788451374	5.499862926	8.683539857	0.003723833	0.041888194
ZNF254	0.354571899	4.348425926	8.652748398	0.003783748	0.04243386
ZNF37A	0.3450102	5.206176516	8.645125379	0.003798733	0.042569842
ZNF204P	0.688807496	1.822404279	8.606679125	0.003875249	0.043264412
SNAI3-AS1	-0.34815966	3.681076643	8.603619634	0.003881406	0.043300664
AC006460.2	-0.33633765	1.604252443	8.597925983	0.003892891	0.043363771
LILRB2	-0.40380083	7.841513191	8.589325581	0.003910304	0.043460158
CARNS1	0.43715579	3.782711525	8.578402087	0.003932538	0.043674648

RPL14P1	0.377794959	2.705028337	8.576353467	0.003936722	0.043688514
SNHG6	0.423796962	4.708673265	8.544471724	0.004002432	0.044318599
MKRN3	0.961300646	1.177422342	8.53928917	0.00401322	0.044405012
ARHGAP42	0.617124007	1.917377491	8.534491857	0.004023233	0.044462587
HNRNPH2	0.322686277	5.861870066	8.522327346	0.004048737	0.044599041
SPHK1	-0.4678844	2.135279155	8.507838515	0.004079332	0.044836497
ZNF860	1.000942534	3.175942895	8.499467164	0.004097117	0.044932426
PAX5	0.989347354	6.115289803	8.49772307	0.004100833	0.044940056
PDE5A	-0.69283575	4.056226272	8.485917729	0.004126074	0.045083867
PC	-0.34682657	2.512988086	8.478036846	0.004143012	0.045235738
ZNF10	0.373845734	2.945333747	8.475286437	0.004148941	0.045267257
CMTM2	0.578510277	5.149237526	8.473499569	0.004152797	0.0452683
AL158166.1	-0.53365296	1.454291841	8.465872757	0.004169298	0.045315846
GAS2L1	-0.5470708	4.342718891	8.465745551	0.004169574	0.045315846
FOXP1-IT1	-0.32369279	3.763463932	8.463015722	0.004175497	0.045315846
EPM2A	-0.33036377	3.420884657	8.440666409	0.004224316	0.045588298
RPL21P123	-0.50800703	3.267294471	8.437047197	0.004232276	0.045601808
ATP23	0.401525203	2.127461226	8.435984577	0.004234617	0.045601808
LINC02284	-0.43471366	2.735963887	8.433716178	0.004239617	0.045621096
TGFB1I1	-0.86451267	1.218554242	8.425354614	0.004258101	0.04578689
HOXB-AS1	-0.57455165	1.765653008	8.423052721	0.004263204	0.045808664
CTDSPL	-0.78953064	2.929208703	8.3939396	0.004328292	0.046287924
CTTN	-0.84745157	4.197619719	8.384486568	0.004349645	0.046469073
AC079316.2	-0.51397802	3.117450834	8.379073151	0.004361922	0.046533425
PANK1	0.511281429	1.539764533	8.368816366	0.004385282	0.046715651
WASH7P	0.452926799	3.084488802	8.346828098	0.004435795	0.047085235
KIR2DL3	-0.97372041	1.194994097	8.344266819	0.004441718	0.0471145
CSNK1G2P1	-0.38951959	2.564988161	8.317227432	0.004504744	0.047681082
BTBD3	-0.33444838	2.317757441	8.308636216	0.004524962	0.047809752
PRKAR2B	-0.65572946	5.540820745	8.307015193	0.004528787	0.047809752
AC108066.2	-0.47547925	1.77687639	8.305485875	0.004532399	0.047809752
H2AC19	2.025305267	1.838572636	8.305246993	0.004532963	0.047809752
AC016739.1	0.369813638	2.872980357	8.286131317	0.004578367	0.04822028
DNAJB9	0.330692688	3.640703247	8.268373105	0.004620966	0.048550607
GP6	-0.74485843	2.264655401	8.265146665	0.00462875	0.048550607
DUSP6	-0.33577336	7.881411912	8.26372326	0.004632188	0.048550607
E2F3-IT1	-0.41859731	2.199594351	8.260829836	0.004639185	0.048550607
RPL12	0.382206152	8.112423379	8.257739389	0.00464667	0.048550607
MT2A	-0.58643468	5.275220971	8.257415572	0.004647455	0.048550607
SULF2	-0.4901208	7.823858834	8.250295905	0.00466475	0.048607218
AL160272.1	-0.57172085	2.849347044	8.249603666	0.004666435	0.048607218
TMSB4XP8	0.413185008	2.444126781	8.247881491	0.00467063	0.048607218
BLK	0.837779636	5.427765613	8.241428662	0.004686383	0.048653575
TIMP1	-0.36413756	5.301079487	8.240701493	0.004688162	0.048653575
H4C1	0.501968606	1.242145051	8.227096615	0.004721568	0.048932012
TRIM66	-0.38224569	5.326596233	8.212655386	0.004757295	0.049075562
KLF7-IT1	-0.37833573	2.91607582	8.209258096	0.004765741	0.049075562
S100Z	-0.3941704	2.643973072	8.202404688	0.004782825	0.049156076
IRF8	0.470283197	6.529678464	8.192178396	0.004808434	0.049317102
DIXDC1	-0.57056785	1.289808009	8.180858082	0.004836948	0.049432367
AL390957.1	-0.72329274	1.726274029	8.162124296	0.004884519	0.04982276
IFIT5	0.529632381	5.493496265	8.158701601	0.004893262	0.049868998

DEGs at 16 weeks post-infection relative to controls					
**external_gene_name**	**logFC**	**logCPM**	**F**	**PValue**	**FDR**
ZNF845	0.73213714	4.438570259	40.75882949	2.03E-09	3.02E-05
RPLP0P6	0.75611232	3.59036086	37.17201065	8.74E-09	3.03E-05
HMGB1P6	0.774937938	2.574281538	37.15628592	8.79E-09	3.03407E-05
USP1	0.401816101	5.444246265	35.76174691	1.56E-08	3.03407E-05
SAMD9	0.776134869	7.505867782	35.48197355	1.76E-08	3.03407E-05
AC090220.1	-0.49066919	2.032680178	35.37381838	1.84E-08	3.03407E-05
PNPLA8	0.444748095	5.196192347	35.36731582	1.84E-08	3.03407E-05
TAF7	0.641905034	6.315723235	35.07063577	2.08E-08	3.03407E-05
HMGB1P5	0.832195872	3.65600296	34.81290822	2.32E-08	3.03407E-05
TMEM70	0.424612967	3.524827948	34.68461198	2.45E-08	3.03407E-05
B2M	0.676291949	11.39888057	34.24416345	2.94E-08	3.12596E-05
HMGB1	0.53776269	6.811912093	33.39069675	4.21E-08	3.91242E-05
AF131215.4	-0.59194474	2.543524711	32.83127161	5.32E-08	4.6608E-05
BTN2A3P	-0.7517085	2.650491835	32.37177993	6.47E-08	5.19912E-05
TMCO1	0.529648068	4.868060281	31.79097281	8.27E-08	5.86093E-05
EVI2A	0.97587665	5.94773969	31.6253547	8.87E-08	6.00248E-05
TMX1	0.481443747	4.993232828	31.13217316	1.09E-07	6.78828E-05
MT-ATP6	-0.65074736	10.37492861	30.91056201	1.20E-07	7.14465E-05
GSKIP	0.514780179	3.881474565	30.64449556	1.35E-07	7.14465E-05
AC009093.7	-0.56415346	2.921317576	30.59694769	1.38E-07	7.14465E-05
LINC00909	0.449615109	4.379481994	30.56958088	1.39E-07	7.14465E-05
AC245060.5	1.735041233	3.238469297	30.13534149	1.68E-07	8.31964E-05
ZNF271P	0.352280014	4.412198523	29.99480515	1.78E-07	8.55221E-05
ZNF765	0.59903069	4.118063932	29.39721194	2.30E-07	0.000107158
MT-ATP8	-0.6961091	6.823127563	29.19752059	2.51E-07	0.000113265
ELMOD2	0.366792469	4.711419895	28.92714659	2.82E-07	0.000120077
CAPZA1	0.466576813	7.915943291	28.56634224	3.30E-07	0.000129638
MT-ND4	-0.64778307	11.37186145	28.35903169	3.61E-07	0.000136509
ZNF721	0.619396657	5.382499824	28.23112735	3.82E-07	0.000138577
AC010615.1	0.898611084	1.73240342	28.1132925	4.02E-07	0.000141791
BLOC1S2	0.520074388	4.087503436	28.06891419	4.10E-07	0.000141791
THAP5	0.416904806	5.217722258	27.99569931	4.23E-07	0.000143056
ANKRD12	0.416115218	7.948693009	27.91479716	4.38E-07	0.000144893
PPIG	0.530925874	6.024874322	27.75883901	4.69E-07	0.000151713
WDR89	0.671472447	3.585722224	27.51082668	5.23E-07	0.000160949
SNHG29	0.647867325	5.837859636	27.43869393	5.39E-07	0.000160949
COMMD8	0.792143224	2.739747389	27.36534865	5.57E-07	0.000162493
RBAK	0.526571891	4.820194347	27.20979337	5.96E-07	0.000164959
DNTTIP2	0.520219912	4.810240264	27.19930298	5.99E-07	0.000164959
MRPL50	0.616316145	3.436685395	27.18261298	6.03E-07	0.000164959
ZNF480	0.642889404	4.252913071	27.08799687	6.29E-07	0.000164959
MYL12B	0.443532618	6.830898069	27.08056017	6.31E-07	0.000164959
RPS3A	1.640210414	7.479813137	27.07680876	6.32E-07	0.000164959
MT-ND4L	-0.63460654	8.609928146	26.94318173	6.70E-07	0.000168828
C1GALT1C1	0.397890742	3.699777838	26.92710871	6.75E-07	0.000168828
MRPL47	0.616533545	2.75151606	26.87240612	6.91E-07	0.000168828
MED7	0.378895677	3.737414623	26.8218767	7.07E-07	0.000168828
ESCO1	0.387908816	5.560729761	26.75301218	7.28E-07	0.000168828
ZNF267	0.511952016	6.323795396	26.72452935	7.37E-07	0.000168828

OTUD6B-AS1	0.689294583	4.400315807	26.61677824	7.73E-07	0.000173265
POU5F2	-0.48306243	4.121237783	26.57902389	7.86E-07	0.000173265
RRN3P2	-0.45266834	2.576569363	26.55660569	7.94E-07	0.000173265
PHF5A	0.499522341	3.460053942	26.47785832	8.22E-07	0.000174721
HAT1	0.506066476	4.073665385	26.34129111	8.73E-07	0.000180539
RPL41	1.252944935	6.72542128	26.33941876	8.73E-07	0.000180539
ZNF836	0.45029995	4.090517704	25.84275916	1.09E-06	0.000218681
MIR17HG	0.538052821	3.232310473	25.80293835	1.11E-06	0.000219595
CAPZA2	0.460527159	6.629014263	25.74255963	1.14E-06	0.000222568
AC010359.3	-0.50226647	3.243426632	25.59547576	1.21E-06	0.000228797
DUT	0.442528557	5.037869813	25.48985919	1.27E-06	0.000235966
IGHG1	-1.37088445	5.0785386	25.46650612	1.28E-06	0.000235966
RMI1	0.36112286	3.591724456	25.30280109	1.38E-06	0.000240344
AL162578.1	-0.51819452	2.190247707	25.29704183	1.38E-06	0.000240344
HSPA13	0.396771589	4.324614629	25.27286036	1.40E-06	0.000240344
MRPL40	0.603115335	2.033264739	25.17707361	1.46E-06	0.000240344
SS18L2	0.530092818	2.620862647	25.16254828	1.47E-06	0.000240344
RSL24D1	0.896032317	4.45901252	25.11718271	1.50E-06	0.000242568
ZNF268	0.53771394	4.265631582	24.96342815	1.61E-06	0.000254508
NMD3	0.481710328	4.310545746	24.96063725	1.61E-06	0.000254508
SNRPD2	0.845422703	4.322332931	24.92640336	1.63E-06	0.000255692
MT-ND5	-0.69918251	10.733952	24.75056742	1.77E-06	0.000268036
DPM1	0.465733318	4.360205728	24.64600519	1.85E-06	0.000277977
ZNF273	0.466530959	3.224110673	24.48235747	1.99E-06	0.000294588
KRCC1	0.467928162	4.532636428	24.47096291	2.00E-06	0.000294588
SF3B6	0.705135485	3.847859724	24.40235549	2.06E-06	0.000300768
ZNF525	0.675112807	4.148412754	24.35543486	2.11E-06	0.000304153
ERH	0.583355815	3.639242711	24.29002394	2.17E-06	0.000307955
MALAT1	0.36634006	14.5325294	24.26210785	2.19E-06	0.000307955
EEF1A1	0.667202589	11.75666527	24.24708402	2.21E-06	0.000307955
ANKRD49	0.546279637	4.495045557	24.24232092	2.21E-06	0.000307955
RPS9	0.431896542	8.076118135	24.1700397	2.29E-06	0.000309492
SLU7	0.358915216	5.748819647	24.15936616	2.30E-06	0.000309492
AC009093.2	-0.41435325	3.48510469	24.14907076	2.31E-06	0.000309492
MT-ND1	-0.66452968	10.94969679	24.02517827	2.44E-06	0.000324208
ZNF816	0.566237945	3.396378476	23.93473279	2.54E-06	0.000334618
MTATP6P1	-0.59469187	7.341486895	23.89811368	2.58E-06	0.000337167
RPL9	1.225642721	6.699960312	23.80521396	2.69E-06	0.000345938
THAP12	0.345956413	5.69948271	23.76365533	2.74E-06	0.000346354
FAM200A	0.381865108	2.943689331	23.76117933	2.75E-06	0.000346354
HSP90AA1	0.764693664	7.524365234	23.61229928	2.94E-06	0.000361097
RPL7P9	1.533809784	4.556250277	23.56772759	3.00E-06	0.000363076
RPL7	1.402068652	7.20424667	23.46477981	3.14E-06	0.000376497
ATP6V1D	0.335577425	4.43855643	23.25559825	3.45E-06	0.000400364
ANKRD44-IT1	-0.35853076	5.509925127	23.10025478	3.70E-06	0.000414794
ZNF830	0.322079222	4.120812068	23.09376349	3.71E-06	0.000414794
EEF1A1P5	0.677006263	7.201983178	23.04765607	3.78E-06	0.000417229
DNAJA1	0.508743121	6.08875305	23.01942236	3.83E-06	0.000419466
CASP3	0.358270929	4.794590707	22.95314319	3.95E-06	0.000424158
TRIAP1	0.657211852	1.856193365	22.94636901	3.96E-06	0.000424158
AC007390.1	0.568865789	2.250957418	22.74296099	4.34E-06	0.000451941

RPL7P1	1.483478791	4.141883864	22.66414855	4.50E-06	0.000461866
EIF3E	0.63974489	6.630297906	22.61519258	4.60E-06	0.000464755
GTF2B	0.345235921	4.552035022	22.61178717	4.61E-06	0.000464755
ZBED5	0.410157044	6.165765055	22.57547204	4.68E-06	0.000464755
TWF1	0.342747835	4.549477169	22.55646854	4.72E-06	0.000465643
CENPQ	0.756843226	1.142248237	22.47413027	4.90E-06	0.000473903
CCDC59	0.446048766	3.982614502	22.40384247	5.06E-06	0.00048607
ERGIC2	0.358004632	5.509092661	22.30701777	5.29E-06	0.00049509
RPS7	1.26748705	6.424591687	22.2460154	5.44E-06	0.000499545
CDC37L1	0.381015287	4.015283123	22.18162872	5.60E-06	0.000508075
PYURF	0.41587663	4.558424166	22.10726988	5.79E-06	0.000519178
SCARNA21	0.799569467	7.179194953	22.0787205	5.87E-06	0.000520849
CHMP5	0.526724035	4.559798768	22.07380784	5.88E-06	0.000520849
RPL26L1	0.78414095	1.508331332	21.94624197	6.23E-06	0.000539083
UFM1	0.406499901	5.184399642	21.93311497	6.27E-06	0.000539174
SRP9	0.398726054	5.579772159	21.89265938	6.38E-06	0.000546024
TIGD7	0.483511213	3.873963774	21.83461879	6.55E-06	0.000551082
SERINC1	0.371381617	7.392118084	21.81008285	6.63E-06	0.000551082
MYL6	0.52886228	7.420269411	21.77519615	6.73E-06	0.000552325
RPL27	0.992405095	7.133677371	21.77258726	6.74E-06	0.000552325
TRMT10C	0.574336888	3.169908261	21.75469245	6.80E-06	0.000552325
SNX4	0.486756721	3.804904978	21.74457897	6.83E-06	0.000552325
AP003117.1	0.791499368	1.219770015	21.70498148	6.95E-06	0.000553836
BDH2	0.650398946	2.225493039	21.70304888	6.96E-06	0.000553836
LAMTOR3	0.372944423	4.65843759	21.64272032	7.15E-06	0.000564948
RPL4	0.552031659	9.213623018	21.62968974	7.20E-06	0.000564948
RPS3AP6	1.698159967	1.322267143	21.58714002	7.34E-06	0.000565707
RPS7P1	1.545178938	3.590916232	21.56532479	7.41E-06	0.000566962
PTRHD1	0.522963794	3.021079558	21.53321956	7.52E-06	0.000568018
NUDCD1	0.555824977	3.516056126	21.46086222	7.77E-06	0.000578256
RPL21	1.135150032	7.290731203	21.43823012	7.85E-06	0.000581349
ZNF14	0.468669131	4.024589544	21.34035866	8.21E-06	0.000604893
ATP6V1G1	0.448648648	5.862541408	21.31551264	8.30E-06	0.000608779
RPLP0	0.506176619	8.622583797	21.29073882	8.40E-06	0.000612029
DYNLT3	0.573112092	3.421856282	21.28238452	8.43E-06	0.000612029
CBX3	0.384910454	5.582854245	21.20187973	8.75E-06	0.000622981
TIMM8B	0.744609657	2.943052621	21.19374639	8.78E-06	0.000622981
LARP7	0.462567794	4.614246916	21.18149854	8.83E-06	0.000622981
ZNF708	0.502867382	5.346714827	21.10869818	9.13E-06	0.000635492
TAF13	0.401699391	2.509299774	21.05873383	9.34E-06	0.000641896
RPL3	0.523276521	9.435438186	21.05232919	9.37E-06	0.000641896
RPL30	0.704190454	7.902191352	21.04067645	9.42E-06	0.000641896
NANOGP4	-0.68946451	1.142037771	21.03364634	9.45E-06	0.000641896
IGBP1	0.378553293	4.89203383	20.98592069	9.65E-06	0.000653089
ACTR6	0.500038831	3.233957464	20.96740236	9.74E-06	0.000655668
GIMAP2	0.427562355	5.945186381	20.92647522	9.92E-06	0.000663004
AC099336.2	1.42871835	2.200403514	20.84776726	1.03E-05	0.000683323
RPS27	1.189816419	8.501661424	20.80935312	1.05E-05	0.00068772
NDUFA5	0.487806981	4.069596614	20.80649278	1.05E-05	0.00068772
ZNF583	0.544086979	2.842384935	20.80474159	1.05E-05	0.00068772
AL513412.1	-0.4851378	1.284535942	20.78994205	1.06E-05	0.000689365

AC125612.1	-0.36258076	1.957748489	20.77250615	1.06E-05	0.000689691
ZNF252P	0.377742045	4.822091629	20.76970319	1.07E-05	0.000689691
NDUFA4	0.648612073	3.981898303	20.76039856	1.07E-05	0.000689691
EEF1B2	1.204627875	5.581212489	20.68628183	1.11E-05	0.000707416
GON7	0.516972568	2.132980806	20.62270075	1.14E-05	0.000718784
KIAA1586	0.501583853	4.016062293	20.57084976	1.17E-05	0.000730239
ZNF441	0.588248582	3.938916297	20.46922981	1.22E-05	0.000756999
S100A8	1.433492511	8.381231209	20.46524125	1.23E-05	0.000756999
GPR52	-0.54545492	2.462638721	20.44513205	1.24E-05	0.000760871
NDUFAF8	0.395786075	3.196960897	20.41083526	1.26E-05	0.000766627
MIR3609	1.238108037	3.270335753	20.3614216	1.29E-05	0.000777872
MT-ND2	-0.59603383	10.65565723	20.33402411	1.30E-05	0.000783918
RPSA	0.578489377	8.009834343	20.32522475	1.31E-05	0.000783918
CLIP2	-0.41743045	4.611848468	20.31577584	1.31E-05	0.000783918
RPL23	1.262589509	6.902863531	20.30953409	1.32E-05	0.000783918
VBP1	0.497240592	3.679568547	20.25779225	1.35E-05	0.000795922
PSMC6	0.405847674	4.984440064	20.25059935	1.35E-05	0.000795922
TAX1BP1	0.370353131	6.894350801	20.23811869	1.36E-05	0.000797356
RPL39	1.285820721	4.66441557	20.12903081	1.43E-05	0.00082173
MT-CYB	-0.55412141	10.61729112	20.12296936	1.44E-05	0.00082173
ATP5PB	0.394986308	5.528006826	20.10392668	1.45E-05	0.000825452
TXNDC17	0.437702293	2.886749554	20.03251095	1.50E-05	0.000849826
MRPS33	0.598101124	2.808733526	19.99526477	1.52E-05	0.000858002
IFT20	0.352673203	3.28551363	19.85518814	1.62E-05	0.000898293
MT-CO2	-0.46818454	10.57929946	19.83668901	1.64E-05	0.000899314
TOMM7	0.934385701	4.668448919	19.82490344	1.65E-05	0.000900899
SRGN	0.657094578	9.245039464	19.8012191	1.66E-05	0.000905664
RANBP6	0.475251469	4.715674934	19.79656384	1.67E-05	0.000905664
RDH14	0.468897825	2.991085492	19.77099506	1.69E-05	0.000906947
RPL13A	0.51800283	9.917377292	19.67249219	1.77E-05	0.000932371
GMFB	0.364691538	5.187311376	19.64852838	1.79E-05	0.000939432
ZNF594	0.546181173	4.468267368	19.5017529	1.91E-05	0.000988011
CD52	0.679435297	7.211695234	19.39161139	2.01E-05	0.001029019
SFR1	0.478147464	1.943301648	19.37409862	2.03E-05	0.001030583
METTL18	0.721534952	1.701235486	19.37355768	2.03E-05	0.001030583
NDUFS5	0.716242154	3.56772415	19.33137839	2.07E-05	0.001044998
CCT2	0.471050235	4.920243352	19.30262321	2.10E-05	0.001044998
RIOK2	0.433857845	3.841893298	19.29988998	2.10E-05	0.001044998
ZNF138	0.478758357	3.779888492	19.25295553	2.15E-05	0.001060906
LIG4	0.372933824	4.120056273	19.22390279	2.17E-05	0.001064696
C12orf57	0.536149261	4.828563459	19.11878391	2.28E-05	0.001099864
HSP90AA2P	0.872467736	0.998704155	19.08392749	2.32E-05	0.001114214
CTNND1	-0.37624628	2.692232237	19.07586936	2.33E-05	0.001114798
HIGD1A	0.386510191	3.248587792	18.95265072	2.47E-05	0.001169249
FBXO22	0.343880774	5.11169055	18.91356148	2.51E-05	0.001184184
COPS2	0.399199576	5.198828397	18.90755676	2.52E-05	0.001184184
FAU	0.490885388	7.270940606	18.86985742	2.56E-05	0.001196127
SACS	0.424524906	5.935030794	18.859344	2.58E-05	0.001198239
RPL6	0.700694769	7.737542293	18.84786045	2.59E-05	0.001200302
OSTC	0.460427641	3.876201598	18.80219966	2.65E-05	0.001215357
TPT1	0.847948592	9.742268755	18.76526908	2.69E-05	0.001228859

SCOC	0.563464574	3.09585851	18.74470616	2.72E-05	0.001230693
RPS3AP26	1.655352781	2.893294812	18.74246692	2.72E-05	0.001230693
Z83843.1	-0.34476274	4.983267993	18.73670818	2.73E-05	0.001230693
EEF1A1P6	0.824006938	3.146506067	18.72940159	2.74E-05	0.001230693
COPS4	0.452016168	3.5833884	18.65511471	2.83E-05	0.001260436
LRRC37A15P	-0.45102003	1.761399989	18.65240514	2.84E-05	0.001260436
SUB1	0.354844426	6.09908609	18.59898726	2.91E-05	0.001288388
ZNF813	0.511799384	3.122461882	18.57985896	2.93E-05	0.001292246
ZNF184	0.427889013	3.633625298	18.53581954	3.00E-05	0.001311319
RPS18	0.881273564	8.111571246	18.52377954	3.01E-05	0.001312946
ZC3H15	0.41716626	5.082222439	18.50486317	3.04E-05	0.001315757
AC010343.1	0.668291671	2.829917259	18.50352674	3.04E-05	0.001315757
ZNF493	0.376395768	5.981224474	18.43694735	3.14E-05	0.001349465
CIAO2A	0.437818441	4.026588755	18.42968962	3.15E-05	0.001350146
ATP5F1C	0.45286702	5.152643731	18.39538884	3.20E-05	0.001368016
RPL15P3	0.534741517	4.141519057	18.33343165	3.29E-05	0.001404177
RPL41P1	1.334532891	0.899833344	18.31996597	3.31E-05	0.0014081
AC006141.1	0.360074392	3.477370874	18.31203269	3.33E-05	0.0014081
LPAR6	0.766726587	4.854232682	18.3091594	3.33E-05	0.0014081
ZNF443	0.491110933	1.774737812	18.29651463	3.35E-05	0.001412439
CHN2	-0.45961409	3.377497266	18.22840877	3.46E-05	0.001451749
ATP5MPL	0.533646264	4.196804437	18.22575873	3.46E-05	0.001451749
PFDN5	0.886726264	6.04815125	18.19099232	3.52E-05	0.00147054
ZNF615	0.356855607	3.925000466	18.17559068	3.55E-05	0.001473787
RPL13AP5	0.55190046	5.408533355	18.13669865	3.61E-05	0.001492533
ZNF146	0.349423534	5.401183107	18.12564908	3.63E-05	0.00149612
DMAC1	0.488467198	3.75559924	18.11340789	3.65E-05	0.001500277
OBI1	0.456042346	4.078003204	18.10793007	3.66E-05	0.001500277
CMTR2	0.384406362	5.031229761	18.06250816	3.74E-05	0.001519983
ZNF432	0.398006765	3.642703825	18.03491469	3.79E-05	0.001535571
ZCCHC10	0.495449809	3.351720328	17.97307708	3.90E-05	0.001567924
SLIRP	0.444698044	2.901778792	17.95485629	3.93E-05	0.001577117
CEBPZ	0.463451496	4.864749215	17.93040071	3.98E-05	0.001584597
DNAJC3-DT	0.60253944	3.080491322	17.92759971	3.98E-05	0.001584597
ZNF616	0.355522819	3.486498088	17.82123649	4.19E-05	0.001644432
ZNF624	0.38578193	3.351152081	17.82032981	4.19E-05	0.001644432
RPS15A	0.936731359	6.717370465	17.81259456	4.20E-05	0.001646072
ZNF781	0.655428361	1.576418898	17.78887544	4.25E-05	0.001660142
CAP2P1	-0.3580522	2.326911738	17.77472909	4.28E-05	0.001662483
PSMA4	0.456836255	5.257304275	17.7628616	4.30E-05	0.001667423
ZNF22	0.533689943	3.892214616	17.7414294	4.35E-05	0.001679923
ZNF92	0.622565872	3.989527971	17.7206177	4.39E-05	0.001692037
ZNF761	0.472142997	3.474557541	17.70694436	4.42E-05	0.001698545
ANXA1	0.604082331	7.034001691	17.65854781	4.52E-05	0.001728697
ARMT1	0.32860801	4.294803478	17.63965947	4.56E-05	0.001736338
BX679664.3	1.523585517	2.15790025	17.63826361	4.56E-05	0.001736338
MT-CO1	-0.50319943	12.19007111	17.61917296	4.60E-05	0.001747528
COX7A2	0.485367321	4.394235632	17.578246	4.69E-05	0.00176048
SNORD13	1.002385376	0.728015785	17.55978541	4.73E-05	0.001761102
DIAPH1	-0.36430837	8.114559212	17.44610891	4.99E-05	0.00182597
RPS20	0.739471679	7.661248579	17.40999527	5.08E-05	0.001852751

ZNF416	0.34081482	2.908644573	17.39267628	5.12E-05	0.001860221
AP000547.3	-0.97676585	3.574525845	17.39107906	5.12E-05	0.001860221
LSM8	0.359674746	6.153417777	17.36639174	5.18E-05	0.001872856
KIF20B	0.467493916	4.069383335	17.32586423	5.28E-05	0.001899762
CENPE	0.61811818	2.236044582	17.3017588	5.35E-05	0.00191685
COX7B	0.618206977	3.480533591	17.27507691	5.41E-05	0.001925518
SVIP	0.486335254	4.441448243	17.27185772	5.42E-05	0.001925518
ZNF141	0.416867252	5.76126312	17.25264345	5.47E-05	0.001933985
BACH1-IT2	-0.4815631	3.053866408	17.25095602	5.47E-05	0.001933985
DDIT3	0.486968356	3.212007032	17.24743571	5.48E-05	0.001933985
RPS23	0.712128675	7.777783366	17.24013359	5.50E-05	0.001936073
WDR5B	0.355752614	3.647161828	17.22553083	5.54E-05	0.001944863
SNX16	0.392780355	3.396554663	17.1969581	5.62E-05	0.001963125
AC008038.1	0.425541958	6.480178981	17.19508893	5.62E-05	0.001963125
TAF9	0.39794484	3.55371047	17.19079239	5.63E-05	0.001963125
NDUFB3	0.864760024	3.052139735	17.16843158	5.69E-05	0.001975863
TMEM123	0.338920094	7.996496601	17.16161356	5.71E-05	0.001976471
SMIM19	0.324966913	2.809066589	17.1057429	5.86E-05	0.002024613
ZNF780B	0.368082525	5.716210755	17.08550353	5.92E-05	0.002034624
NAT1	0.344172944	2.717163775	17.07763973	5.94E-05	0.002037493
CBX3P2	0.408525695	2.009765399	17.06824035	5.97E-05	0.002041859
POLR1F	0.401876008	3.489404972	17.01669759	6.12E-05	0.002082641
SGO2	0.561534354	2.08276614	16.99741446	6.17E-05	0.002096918
PIGK	0.35212187	4.218601985	16.98421174	6.21E-05	0.00210248
ZNF420	0.407644857	3.382383254	16.98217886	6.22E-05	0.00210248
RPL35	0.763307224	6.993331957	16.94413861	6.33E-05	0.002129312
RPL11	0.84051315	7.855884066	16.88320403	6.51E-05	0.002175862
RPL22L1	0.781654236	2.508653762	16.87111903	6.55E-05	0.002180262
TMEM126A	0.534963849	1.834032583	16.86347383	6.58E-05	0.002180262
NFYB	0.49034369	3.740342574	16.86261441	6.58E-05	0.002180262
RPL39P3	1.305153792	2.142135745	16.80318363	6.77E-05	0.002227609
IGLV1-47	-1.03521592	1.504205745	16.76039161	6.90E-05	0.002258242
ZNF888	0.354361335	3.500949118	16.70981296	7.07E-05	0.002292874
ZNF260	0.469288937	4.022887431	16.67886581	7.18E-05	0.002293741
IDI1	0.41915365	5.006315329	16.6628913	7.23E-05	0.002293741
UBL5	0.409084299	4.984266142	16.65731983	7.25E-05	0.002293741
JRKL	0.436362711	3.528311333	16.65246323	7.27E-05	0.002293741
AL158211.5	0.482898755	2.259584609	16.65164362	7.27E-05	0.002293741
ZNF181	0.53360022	3.606859835	16.64980865	7.28E-05	0.002293741
RPL31	1.285159031	6.925107753	16.64932199	7.28E-05	0.002293741
AL050341.2	0.495961306	1.742481273	16.62335039	7.37E-05	0.002303529
PFDN4	0.611837515	1.99681016	16.60564969	7.43E-05	0.002317561
SMIM30	0.698540566	1.874808984	16.5984582	7.46E-05	0.002317561
MRPL3	0.412638463	4.676178644	16.58959582	7.49E-05	0.002321214
RPL34	1.436163162	5.993515751	16.56154371	7.59E-05	0.002337715
CASP8AP2	0.331560637	5.782837057	16.55248127	7.62E-05	0.002342943
BCL2A1	0.904630179	4.511517003	16.54556429	7.64E-05	0.002345802
ZNF28	0.359801015	4.385869786	16.52982103	7.70E-05	0.002353696
RPA3	0.401927197	2.549968814	16.50482281	7.79E-05	0.002365107
RPS7P11	1.374673497	1.274870915	16.4757929	7.90E-05	0.00239033
SNRPE	0.587452319	3.045303983	16.45673132	7.97E-05	0.002398617

HINT1	0.548544952	5.251119471	16.45571215	7.98E-05	0.002398617
POLR2K	0.788927847	2.385383145	16.43440534	8.06E-05	0.00241814
SCARNA7	1.202080567	5.980954278	16.34323426	8.42E-05	0.002481314
ZNF208	1.019474174	2.609068837	16.31306339	8.54E-05	0.002509609
MRPL15	0.409381079	2.927005895	16.31018607	8.55E-05	0.002509609
SERPINI1	0.513580936	1.94562878	16.30583134	8.57E-05	0.002509863
TRAPPC2B	0.520190116	1.639861801	16.30122318	8.59E-05	0.00251043
ZNF32	0.486002781	3.133421343	16.28032317	8.67E-05	0.002520628
AGGF1P2	1.006991092	0.922618503	16.27818535	8.68E-05	0.002520628
XRCC4	0.447362765	2.828459556	16.22981436	8.88E-05	0.002566957
GNL3	0.464150503	4.250962339	16.20756367	8.98E-05	0.002578688
ZNF850	0.34006567	2.566251794	16.20713476	8.98E-05	0.002578688
RBPMS2	-1.33178922	1.489288608	16.13916082	9.28E-05	0.002627079
UQCRB	0.936653003	4.743208687	16.13683159	9.29E-05	0.002627079
TXNDC9	0.387704986	3.683538377	16.11800419	9.37E-05	0.00264313
CHMP4BP1	-0.39345529	1.700231216	16.10142756	9.44E-05	0.002649578
RWDD1	0.435028483	4.107070481	16.0587162	9.64E-05	0.002680868
COX7C	0.84045655	4.747311352	16.05032308	9.68E-05	0.002686597
HMGB2	0.638484711	5.539167591	16.04028267	9.72E-05	0.002694464
ZNF879	0.459260591	3.141769553	15.98105623	1.00E-04	0.002751182
CARD8-AS1	0.364883721	4.405487219	15.97082894	1.01E-04	0.002756385
PSMC2	0.35037416	4.313319273	15.93006995	1.02E-04	0.00279826
SNHG19	0.566291858	1.193555202	15.92058987	1.03E-04	0.002799258
COQ5	0.338783365	3.071713905	15.89363247	1.04E-04	0.002821962
CD69	0.625994	3.263200127	15.88876362	0.000104517	0.002822917
HSP90B2P	0.503750768	1.248925872	15.87218516	0.000105348	0.002830455
ZNF226	0.357151683	5.264908432	15.86955548	0.000105481	0.002830455
RPL27A	0.468381108	8.6331405	15.85058808	0.000106441	0.002843894
IGHG4	-1.13937944	2.714317952	15.83400783	0.000107287	0.002859827
RPS14	0.561926602	8.187313681	15.81487324	0.000108273	0.002870908
CSTA	0.865931353	4.23092559	15.80516172	0.000108776	0.002870908
BRIX1	0.394871943	3.20811246	15.75831523	0.00011124	0.002894173
MTND6P11	-0.92516278	1.590082549	15.7484843	0.000111764	0.002897677
C15orf61	0.369076357	2.476569764	15.72821859	0.000112852	0.002915736
HCFC1	-0.33770235	7.040323392	15.6654843	0.00011629	0.002964409
CEP57	0.351162408	5.441374936	15.63511369	0.000117993	0.002996535
RPL15	0.385500459	8.717600548	15.61854377	0.000118932	0.003005014
FKBP3	0.392981486	3.75243652	15.58982697	0.000120579	0.003034216
EIF2A	0.334827497	5.448672942	15.58716383	0.000120733	0.003034216
RPF2	0.352602646	2.852960348	15.57028522	0.000121713	0.003044251
DAB2	-0.52158043	4.364159837	15.56339961	0.000122115	0.003048296
NXT2	0.398152747	2.792360479	15.55180405	0.000122795	0.003048296
FPGT	0.364985519	3.259120693	15.54656594	0.000123103	0.003048296
RPL24P4	0.734507121	2.84690179	15.48509705	0.000126783	0.003123825
MRPL48	0.353057934	2.854281325	15.46328515	0.000128116	0.003150622
LIMD1-AS1	0.324418534	2.278348417	15.42149663	0.000130709	0.003188873
NDUFA6	0.477999996	3.612790595	15.40621692	0.000131671	0.003201831
SSB	0.47833437	4.569709372	15.36524237	0.000134285	0.003254728
RBBP8	0.37821543	3.218911548	15.36187111	0.000134502	0.003254728
CAHM	0.35577003	1.54869542	15.33222434	0.000136429	0.003292433
RPS3	0.406154776	8.806665694	15.31596507	0.000137498	0.003305736

COX6C	0.623595164	4.026649505	15.29658996	0.000138783	0.003320537
COMMD6	0.915058941	3.831179191	15.24225545	0.000142453	0.003386552
ADPRM	0.430995893	3.77222818	15.23083732	0.000143236	0.003399749
RPS6	0.6820295	8.413634358	15.2102871	0.000144658	0.003419446
AC079316.2	-0.53962225	3.117450834	15.20888083	0.000144755	0.003419446
MT-ND6	-0.55749857	5.182441748	15.18640919	0.000146327	0.003447805
GIN1	0.341712624	3.2689349	15.17741704	0.000146961	0.003455096
SNRPG	0.514619004	3.613738067	15.16578915	0.000147785	0.003468983
PTOV1-AS1	0.383881599	1.888116892	15.14550483	0.000149233	0.003487582
ACAT1	0.369723364	3.433181245	15.14359434	0.00014937	0.003487582
AC116533.1	1.613455489	4.092635702	15.13735785	0.000149819	0.003487582
TRMT12	0.333592572	3.525832319	15.11217719	0.000151644	0.003520705
RPL24	0.634718302	6.541191963	15.10031507	0.000152512	0.003535335
TMA7	0.765351539	4.090165755	15.07681112	0.000154246	0.003555122
MDH1	0.324710395	4.831541434	15.0758038	0.000154321	0.003555122
PPIL1	0.325700592	2.574926697	15.06088125	0.000155433	0.003575207
NIFK	0.402520108	3.938772986	15.04368728	0.000156725	0.003593805
CHCHD1	0.360359396	3.110931873	15.03587928	0.000157315	0.003600491
TCF7L2	-0.4372385	5.45201654	15.02856009	0.00015787	0.003603411
LYRM2	0.381985344	4.286232333	15.00401777	0.000159746	0.003640654
EID1	0.369891642	5.723688537	14.97009407	0.000162378	0.003694959
RPL26	1.295460575	5.314117273	14.95111255	0.000163869	0.003717528
AF165147.1	-0.52378459	2.268339966	14.92859886	0.000165656	0.003745134
ITGB3BP	0.441067484	3.343642829	14.92429546	0.000166	0.003745134
ZNF613	0.369564362	3.085695303	14.89478222	0.000168378	0.003779484
AF131215.2	-0.42570095	2.359346422	14.8555337	0.000171594	0.003828629
MRPL51	0.478113036	3.439173586	14.85550511	0.000171596	0.003828629
DBI	0.608719692	4.177015099	14.82458018	0.000174174	0.003869845
AC130895.1	-0.56300336	2.022691265	14.81870818	0.000174668	0.003869845
SBDS	0.336742692	4.051412719	14.81781469	0.000174744	0.003869845
RPL21P16	1.1086728	5.103807357	14.81408012	0.000175059	0.003871061
ZNF17	0.326658137	3.619449457	14.792109	0.000176924	0.003898874
ATP5PO	0.41455879	3.697671128	14.7507381	0.000180492	0.003953889
PSMD10	0.366010004	2.909153263	14.74321973	0.000181148	0.003958649
CLIC1	0.329523756	7.517719867	14.72605324	0.000182655	0.003985736
ZNF117	0.553162883	6.529703402	14.6989049	0.000185065	0.004020637
ETAA1	0.444205182	3.950475395	14.69231094	0.000185655	0.00402758
THAP2	0.358059327	3.031873387	14.68869245	0.00018598	0.004028752
ARSD	-0.34839233	4.470733715	14.67909814	0.000186844	0.004039159
RPL35A	0.673108246	6.774043314	14.60869459	0.000193308	0.004130518
MSRA	-0.32620674	4.460749574	14.60714397	0.000193453	0.004130518
AC115223.1	0.774752146	1.199959031	14.58905876	0.000195152	0.004148925
CSNK1G3	0.353773812	5.165486878	14.57579757	0.000196407	0.004163712
RPL6P27	0.699124561	4.639300323	14.56700255	0.000197244	0.004169575
ZNF90	0.535600568	2.402466764	14.51720085	0.000202052	0.004253093
SCARNA9	0.602327294	3.24112427	14.49512737	0.000204221	0.004286627
ATP5PF	0.441528203	3.66489127	14.48464457	0.00020526	0.004290806
DNAJB6P7	-0.39478299	1.769032057	14.48438724	0.000205285	0.004290806
AC241520.1	-0.49548238	1.084298167	14.47635965	0.000206084	0.004301465
RPS25	0.670760206	7.092475491	14.46750706	0.000206969	0.004311505
RPL5	0.573597112	7.600876844	14.46286608	0.000207434	0.004311505

RPS16	0.399317214	8.442585375	14.4359476	0.000210155	0.004349825
ABCE1	0.349256005	5.116118188	14.4011636	0.000213724	0.004411434
H4C2	0.566482875	4.186706134	14.34913974	0.000219178	0.004512746
RPS27A	0.718187232	7.541517788	14.3457173	0.000219542	0.004512746
LSM1	0.324094188	3.430393684	14.33356928	0.000220838	0.004522644
SNHG32	0.350797277	2.978599569	14.31686499	0.000222633	0.004538672
LSM5	0.465448994	2.844697081	14.31377042	0.000222968	0.004539266
GMFG	0.402957039	6.905950367	14.29761983	0.00022472	0.004552
ZNF569	0.359799816	3.146767171	14.27797559	0.00022687	0.00456972
RPS15AP10	0.39714427	2.31733214	14.27793389	0.000226875	0.00456972
RPS24	0.918369886	6.971741523	14.27752442	0.00022692	0.00456972
UQCRQ	0.448133796	3.692456479	14.27407501	0.0002273	0.004571183
HAUS1	0.402594145	2.733205541	14.25919151	0.000228946	0.00459808
FAM200B	0.368868413	4.156229477	14.20055089	0.000235552	0.004688822
COMMD3	0.407756207	2.556847054	14.12174374	0.000244738	0.004793657
AC144831.1	-0.4963773	1.546528925	14.11553129	0.000245477	0.00480051
C1orf198	-0.57971797	3.348775375	14.06370085	0.000251736	0.004891874
AC243919.1	1.492442991	5.607829269	14.05539999	0.000252753	0.004891874
SLC2A14	-1.19960978	0.673066421	14.00746079	0.000258711	0.00498078
SRFBP1	0.357478964	3.429484525	13.96894753	0.000263602	0.005042318
TXN	0.576253791	3.481053728	13.9387024	0.000267509	0.00509739
SHLD3	0.447016572	1.630999573	13.91555347	0.000270538	0.005135399
PRDX1	0.324245946	4.992866767	13.9003837	0.000272543	0.005155722
CD48	0.339712757	7.306114492	13.89958909	0.000272648	0.005155722
MSN	-0.3321134	10.05067094	13.88178907	0.000275021	0.005180834
PPIA	0.348826099	7.665195873	13.86900882	0.000276737	0.005206575
ZNF204P	0.725019458	1.822404279	13.83740704	0.000281028	0.005263331
RPL4P4	0.531555778	1.771866278	13.83638192	0.000281168	0.005263331
ZNF302	0.385363185	4.861170612	13.77557059	0.000289622	0.005374257
ZNF254	0.362847586	4.348425926	13.76105706	0.000291678	0.005398938
ACTBP2	-0.46062982	1.365713555	13.71596757	0.000298159	0.005491595
AC009951.4	-0.34469455	5.710386258	13.68668056	0.000302448	0.005543145
ZFP30	0.404126248	3.564247735	13.60172329	0.000315249	0.005714394
MRPL32	0.442260429	3.284355988	13.59923677	0.000315632	0.005714394
RPS5	0.443037359	7.866378821	13.5827942	0.000318176	0.005753449
ATP5F1E	0.523798634	6.318026862	13.56999893	0.00032017	0.005772324
RPS3AP47	1.628621071	0.741328924	13.54205013	0.000324569	0.005805581
SHLD2	0.361749356	4.472714016	13.43254964	0.000342411	0.006073618
TMEM202-AS1	0.323496568	2.004938203	13.42417557	0.000343816	0.006091279
RPL29	0.340121143	7.997858338	13.3782153	0.000351633	0.006187728
FO393411.1	1.309416072	0.908258257	13.35884514	0.000354982	0.006229111
CHML	0.437692622	4.889790656	13.34582672	0.00035725	0.006247474
VRK1	0.556899601	4.471884514	13.33128583	0.000359802	0.006269993
AC009093.6	-0.44511247	1.571244288	13.2617573	0.000372263	0.006456889
AC122718.2	-0.38294653	2.782631772	13.25260615	0.000373935	0.006478348
RPL10A	0.392811833	7.896890659	13.23954925	0.000376335	0.006504777
ESF1	0.463621777	3.647309027	13.21807695	0.000380315	0.006550753
MT-ND3	-0.39053867	9.026295726	13.17353784	0.000388709	0.006658107
NDUFB2	0.338967462	4.185530211	13.16202604	0.00039091	0.006669541
ZNF510	0.324136451	4.741245616	13.16027697	0.000391245	0.006669541
RPS29	0.800781392	7.864695156	13.14126184	0.000394911	0.006724323

LXN	0.418954527	2.801165572	13.05239012	0.000412513	0.006936736
SRP14	0.429792255	5.825832939	13.01294845	0.000420579	0.007024757
RPS8	0.522316978	8.268662313	12.98440598	0.000426517	0.007084181
RTL5	-0.49822421	1.794401313	12.94839676	0.000434131	0.007194578
FIGNL1	0.369019436	3.384874594	12.94530297	0.000434791	0.00719751
NIPSNAP3A	0.440919041	3.053944773	12.93283923	0.000437463	0.00723369
RPL13	0.337543363	10.01830319	12.92000988	0.00044023	0.007255923
MTND5P14	-0.36312176	2.89552947	12.91757461	0.000440758	0.007255923
ATP5ME	0.502805712	3.554364364	12.88743442	0.000447338	0.007347995
RPL3P4	0.462999217	6.293496732	12.84880078	0.00045592	0.007426005
RPS27L	0.372741127	3.656580464	12.83185754	0.000459736	0.007463386
AC024293.1	0.695991597	4.162971138	12.83122476	0.000459879	0.007463386
CXCR2P1	-0.698823	4.751669347	12.79919435	0.000467186	0.007532679
RPL32	0.450489202	8.046593947	12.78597054	0.000470237	0.007565479
AP001269.4	0.639913291	2.289898842	12.78032786	0.000471545	0.007573823
ZNF675	0.339880638	4.387266954	12.72982718	0.000483419	0.007722619
RPL21P123	-0.48566549	3.267294471	12.7269098	0.000484114	0.007722619
MTATP6P11	-0.39255131	2.56782691	12.72456205	0.000484675	0.007722619
LSM3	0.459512249	3.158683586	12.6322304	0.00050724	0.007954417
JAM3	-0.61306464	3.559370031	12.62405441	0.000509289	0.007956137
AC009093.8	-0.37101372	1.47594452	12.61882717	0.000510603	0.007956151
AC034111.1	0.370846914	1.521442893	12.58716296	0.000518641	0.008024451
ASNSD1	0.387141503	3.551507947	12.58666301	0.000518769	0.008024451
TCEAL8	0.426309595	2.91554928	12.55838166	0.000526059	0.008083734
UQCRH	0.478735152	3.992404572	12.54913893	0.000528464	0.008107834
CAMP	1.32289124	3.265588997	12.5171076	0.000536886	0.008211653
CTSG	1.489525854	0.901174976	12.47832483	0.000547267	0.008344706
AC023157.2	0.477475429	3.95635507	12.43502354	0.000559101	0.008490346
RPL3P2	0.511708734	0.870293054	12.40706155	0.000566881	0.008547441
HYLS1	0.323091128	2.451495442	12.37902055	0.000574794	0.008623075
AL356966.1	-0.42598233	2.164971129	12.34315465	0.000585081	0.008733372
NSA2	0.481583642	5.072728038	12.31209349	0.000594141	0.008849435
CSF1R	-0.3703782	7.055079017	12.29160526	0.000600196	0.008914286
PGBD4	0.363089536	2.830800544	12.28222284	0.00060299	0.008925113
MAGEH1	0.346797781	2.65262881	12.28110352	0.000603324	0.008925113
ZNF84	0.339085593	5.131669575	12.27177125	0.000606117	0.008944476
AC068473.5	0.347375612	2.178361204	12.26162245	0.00060917	0.008949331
EIF4EP2	0.395309186	1.16324261	12.15755945	0.000641397	0.009312455
EEF1A1P13	0.605464968	1.601556309	12.12839691	0.000650737	0.00939308
GAS5	0.513297171	4.787541593	12.11871463	0.000653868	0.009429138
MITF	-0.40513009	2.079901885	12.10264805	0.000659099	0.009447669
ZNF658	0.456680461	2.441503186	12.09851571	0.000660451	0.009447669
AC005912.1	0.942807956	6.665215335	12.04740572	0.000677413	0.009656375
RPL10P16	0.512764243	5.446891602	12.04214259	0.000679184	0.009672366
HNRNPH1P1	-0.32308913	1.762334446	12.02411682	0.000685288	0.009740651
MTCO1P11	-0.35752171	3.538408907	12.01624141	0.000687973	0.009758453
H4C3	0.561445888	5.376836215	11.99769562	0.000694337	0.009775895
GPR183	0.455053006	4.921025851	11.94030678	0.000714414	0.009992396
C7orf25	0.374509446	1.570010666	11.89207263	0.000731747	0.010189077
NDUFAF4	0.458542352	2.405893725	11.88977427	0.000732584	0.010189077
AIF1	0.492416989	6.037684955	11.88140496	0.000735638	0.010222009

RPL14P1	0.360193786	2.705028337	11.85808552	0.000744218	0.010321956
ZNF571	0.388744894	2.817743771	11.82913459	0.000755013	0.010362328
MRPL13	0.381060033	2.78972788	11.82148032	0.000757894	0.010376242
EIF1AY	0.672206742	3.685986317	11.79281722	0.000768781	0.010496333
SRC	-0.39167789	4.794370247	11.71137026	0.0008006	0.010773571
RPL21P75	0.961731834	1.8504607	11.70574968	0.000802845	0.010793075
AC092139.2	-0.43549706	3.984579203	11.67168736	0.000816588	0.010922137
THNSL1	0.478051733	2.323948097	11.67104648	0.000816848	0.010922137
CSMD2	1.032857015	1.292263531	11.64825623	0.000826181	0.010995175
NUDT15	0.362671601	1.722741949	11.64792748	0.000826316	0.010995175
RTCA-AS1	0.336053056	3.420318629	11.636084	0.00083121	0.011006877
LINC00653	0.366005134	1.464593487	11.63402997	0.000832061	0.011006877
ZNF527	0.338590588	3.132376173	11.62169481	0.000837195	0.011064951
GIMAP7	0.46982077	6.737960283	11.60940822	0.000842341	0.011103377
TMEM256	0.590866994	1.124961596	11.60729283	0.00084323	0.011105261
VIL1	-0.81904859	1.332792566	11.59714649	0.000847508	0.011151737
PAPSS1	-0.36598216	5.200657165	11.59045408	0.000850342	0.011179144
LINC01146	-0.41921234	3.425246567	11.56497536	0.000861221	0.011302192
MTND1P11	-0.35583473	3.293754797	11.55102611	0.000867237	0.011361106
ANKRD36BP2	1.061213806	5.408768845	11.54548978	0.000869636	0.011382522
GEMIN6	0.337039962	2.468469841	11.51221201	0.000884204	0.011492334
RASSF1-AS1	0.411263988	1.497063161	11.50857493	0.000885811	0.011503177
ZNF680	0.350500767	3.69691352	11.49446093	0.000892076	0.011554292
CMTM2	0.548979412	5.149237526	11.45634901	0.000909222	0.011725341
MKRN3	0.928265631	1.177422342	11.4486808	0.000912712	0.011749989
AC072022.2	0.677707104	1.233929037	11.44660744	0.000913659	0.011752002
TRMT10A	0.338361524	1.892810549	11.42591908	0.000923154	0.011853645
GFI1B	-0.64247273	4.439343902	11.41673077	0.000927403	0.011887696
CISD1	0.34008504	2.596914565	11.4012641	0.000934602	0.011928595
KBTBD8	0.62015243	3.275631083	11.35052735	0.000958619	0.012146724
CLEC2B	0.439468427	5.387915246	11.30867563	0.000978906	0.012335373
AC099560.2	1.011994844	4.064017343	11.27380777	0.000996142	0.012499655
OCIAD2	0.33904656	3.181060295	11.26251007	0.001001794	0.012549405
POLR2A	-0.34280331	8.012459139	11.21127308	0.001027838	0.01274587
AC015911.4	0.523828958	2.008559604	11.20346267	0.001031869	0.01274587
LTV1	0.326448565	2.87745938	11.20195946	0.001032646	0.01274587
EEF1E1	0.41089391	1.605220413	11.18750096	0.001040156	0.012803645
AL669831.1	-0.36455559	4.404981913	11.18144269	0.00104332	0.012821374
CRISP3	1.538695523	1.877184718	11.17893311	0.001044633	0.012826921
IFT57	0.447785736	3.748963144	11.1757508	0.001046301	0.012836807
NME1	0.452637589	1.139500391	11.1619454	0.001053567	0.012915312
MTND6P5	-0.35901318	2.163611286	11.15481736	0.001057339	0.012940235
HDAC10	-0.34115566	1.739606946	11.12200323	0.001074884	0.01310667
NPM1	0.394583664	6.714545157	11.09333747	0.001090454	0.013236648
CTSA	-0.32883892	6.838621872	11.07633921	0.001099795	0.013317456
NDUFA1	0.539827317	3.597163198	11.02894254	0.001126278	0.013561573
SNHG8	0.544320667	3.390912678	11.02645344	0.001127687	0.013561787
RPS27AP16	0.70992863	3.249836779	11.02372118	0.001129235	0.01356358
EGFL7	-0.62341569	1.134748941	10.9912727	0.001147791	0.013684087
AC092070.2	0.348403752	5.19803925	10.99010793	0.001148463	0.013684087
BACH1-IT1	-0.34813055	3.114550164	10.98193538	0.001153188	0.013704221

TRIM66	-0.34504899	5.326596233	10.95986257	0.001166051	0.013838252
CHI3L2	0.572173	3.020833761	10.93337883	0.001181678	0.014012532
PITPNA-AS1	0.453221391	1.184828916	10.91451448	0.001192939	0.014105755
ZNF572	0.467645196	1.546078515	10.88338956	0.001211761	0.014278252
IFNGR1	0.383823617	7.259579172	10.83783498	0.001239859	0.014574711
ZDBF2	0.456110117	3.334830488	10.80609313	0.001259831	0.014755027
ZNF91	0.336669412	6.55262109	10.79959554	0.00126396	0.014764719
SLC35A1	0.405726507	2.477507828	10.76471966	0.001286359	0.014979341
ZNF567	0.329280618	3.874451545	10.75697819	0.001291386	0.01502612
SNHG5	0.344204087	5.601272983	10.74426216	0.001299687	0.015058906
RPS21	0.677483911	6.387378066	10.74409152	0.001299799	0.015058906
AC130454.1	-0.62080016	1.434585865	10.74241859	0.001300895	0.015058906
IKZF2	-0.35931444	5.806633571	10.74037164	0.001302238	0.015058906
AC007342.3	0.471475498	5.003423592	10.72364896	0.001313259	0.015127293
AC008462.1	0.386240402	1.463770456	10.70453831	0.001325972	0.015230653
TTC30A	0.410527064	1.283176787	10.63121733	0.001375928	0.015702883
LLPH-DT	0.940288402	1.025806216	10.60806453	0.0013921	0.015838889
RHOBTB1	-0.64185877	2.084015857	10.60806136	0.001392102	0.015838889
PRKACB	0.339792033	6.751925864	10.60186198	0.001396466	0.0158595
RPL37	0.444965708	7.843532399	10.596691	0.001400116	0.015878577
SNORD15B	0.977787683	2.825148501	10.59554575	0.001400925	0.015878577
XRRA1	-0.65936502	4.837019933	10.58564981	0.001407942	0.015945964
PRNCR1	-0.42886591	2.583059998	10.57349259	0.001416612	0.015994471
MRPL1	0.409016809	2.694979488	10.56802564	0.001420528	0.01600955
KCNAB3	-0.33199009	2.452819956	10.48068886	0.001484622	0.016519645
RPS12	0.412285117	8.49336645	10.45391539	0.001504858	0.016687995
SPRED2	-0.37143168	2.392148647	10.44605346	0.001510853	0.016729552
IFT74	0.323938352	2.901603743	10.43159733	0.001521942	0.016827295
UBAC2-AS1	0.386865054	1.286559061	10.41221598	0.00153694	0.016905575
PAK1IP1	0.329260977	2.669713049	10.39258421	0.001552285	0.017036218
CRIM1	-0.35756407	3.34105996	10.34327499	0.001591526	0.017389938
CEACAM6	1.282963944	1.533459004	10.30481865	0.001622835	0.017635109
AC004837.4	0.372767521	1.428996312	10.28180193	0.001641876	0.017757554
MANEA-DT	0.378824364	2.438279262	10.27903325	0.001644181	0.017769578
AC079316.1	-0.43221399	3.000791421	10.23624186	0.001680243	0.018054426
YEATS4	0.407365264	2.686726487	10.20408096	0.001707879	0.018324918
KIR3DL2	-0.79437241	1.743337902	10.19276139	0.001717717	0.018417193
AC092620.1	-0.39186212	2.694613855	10.18933396	0.001720707	0.018428955
ABCA13	1.122834115	3.209974603	10.18334842	0.001725942	0.018465468
TDRD9	-0.60105514	2.378681442	10.16855866	0.001738946	0.01857789
SMIM20	0.337543904	2.397485786	10.16439274	0.001742628	0.018603863
CETN3	0.457116169	1.757959491	10.10002587	0.001800532	0.019032291
TMEM144	-0.48364753	2.956949734	10.09988265	0.001800663	0.019032291
CCDC152	0.476332502	2.06493235	10.09156399	0.00180829	0.019085792
ZNF10	0.33042206	2.945333747	10.06756791	0.001830476	0.019247353
AL353708.3	0.324589749	2.076957266	10.0607187	0.00183686	0.019264372
RPL12	0.341323137	8.112423379	10.05785961	0.001839531	0.019273475
AC011676.1	-0.45671611	2.690705261	10.03971896	0.001856575	0.019348421
SERPINE1	-0.68142401	1.018730847	10.02750814	0.001868138	0.019441703
MRPL36	0.323641128	2.26220669	10.00863597	0.001886156	0.019560552
RBIS	0.400201857	3.404792669	9.991475101	0.001902694	0.019650169

RPL13P12	0.623279467	5.485200543	9.977160941	0.001916603	0.019752689
PTGS1	-0.55165951	5.913948207	9.918668419	0.001974531	0.020181339
SENCR	-0.36966465	1.914223919	9.857035008	0.002037517	0.020655534
ARHGAP6	-0.59437777	2.725300599	9.829188034	0.002066647	0.020879731
GPATCH11	0.364104395	4.226421812	9.826845599	0.002069117	0.020890502
DPH5	0.35663844	3.488457108	9.82549685	0.002070541	0.020890701
CX3CR1	-0.3821783	7.534534897	9.795905812	0.002102025	0.021051371
AL354977.2	-0.45751897	1.151508905	9.766684699	0.002133598	0.021235739
ZNF737	0.373641682	5.563147276	9.755405284	0.002145916	0.021274417
BEX2	0.567692944	1.723947588	9.732182986	0.002171505	0.021486924
S100A9	0.544954561	11.1264115	9.691853109	0.002216692	0.021774794
KIF18A	0.452389457	1.449328685	9.688677751	0.002220291	0.021795757
AC026403.1	0.421444965	2.587546555	9.678528041	0.002231833	0.021869995
ZNF354C	0.327549283	3.704649333	9.674237824	0.002236731	0.021899362
IGHV4-59	-0.75128234	2.184908169	9.644471311	0.002271016	0.022163888
AK6	0.481276602	1.259206067	9.636712906	0.002280041	0.022192002
SIPA1L1	-0.32741088	7.623726463	9.588917306	0.002336455	0.022607696
ZNF582	0.338071494	2.030892416	9.573786908	0.002354611	0.022665792
ZNF107	0.414343848	5.972825899	9.517762887	0.002423108	0.023130405
LIPN	0.661214581	4.11330287	9.463014603	0.002492023	0.023590549
IQGAP1	-0.33942071	9.595534986	9.461875931	0.002493478	0.023590549
MIR7845	0.481537571	2.06839951	9.444887412	0.002515282	0.02375154
LRRCC1	0.330876997	2.150234634	9.436049122	0.002526703	0.023814057
IL2RB	-0.34405181	7.420878217	9.432727711	0.002531009	0.023830363
ABLIM3	-0.75121458	2.885013777	9.432244689	0.002531636	0.023830363
ZNF85	0.325115763	2.778166107	9.416443151	0.002552229	0.023978709
ZNF253	0.325616816	4.150617931	9.401302446	0.002572123	0.02413514
ZFHX3	-0.33232932	5.090426495	9.388177623	0.002589497	0.024237037
PDGFA	-0.62643214	2.412024301	9.367297905	0.002617385	0.02437542
GP6	-0.62229074	2.264655401	9.36335971	0.002622679	0.024409452
RPL13AP25	0.588607632	4.548454987	9.320239951	0.002681375	0.024785233
ATP23	0.343628547	2.127461226	9.298677774	0.002711231	0.024896874
CXCL8	0.650666191	3.057883684	9.296419347	0.002714377	0.024904664
PPIAP22	0.659729153	1.844108552	9.279098559	0.002738636	0.025065429
DLG5	-0.48253038	4.35246763	9.250212433	0.00277959	0.025348716
RPS27AP5	0.887632408	0.766334869	9.247223489	0.002783864	0.025348716
TUBB1	-0.62100909	7.74697584	9.239039295	0.0027956	0.025414852
NOC3L	0.354922281	4.081346289	9.22261511	0.002819305	0.025567883
RPS27AP12	0.326437535	1.434852258	9.220812543	0.002821919	0.025571125
RPL18AP3	0.327499714	5.350487798	9.215971688	0.002828952	0.025608555
AC064799.1	1.657805826	1.628718752	9.208502195	0.002839839	0.02566972
LY96	0.441020418	3.734058043	9.202678614	0.002848357	0.025721636
CCDC167	0.37502825	2.41903229	9.201449563	0.002850159	0.025722292
RNU1-4	-0.77819537	2.802068375	9.190653862	0.002866029	0.025834189
CCDC65	0.425493353	2.161911634	9.174600797	0.002889797	0.025985471
UFSP1	0.335708001	1.54705443	9.134334446	0.002950309	0.026338632
PDE5A	-0.56037861	4.056226272	9.107190963	0.002991834	0.026597655
E2F3-IT1	-0.34525306	2.199594351	9.104333076	0.002996241	0.02662093
AC015912.3	0.529408138	1.902032673	9.096324262	0.003008626	0.026715021
NTSR1	-0.77217322	1.008236051	9.082924107	0.003029467	0.02687184
TRAT1	0.452834938	5.269244942	9.08224635	0.003030525	0.02687184

AC084824.1	0.365747413	1.877212381	9.077786285	0.003037497	0.026875165
NRGN	-0.6261034	7.542308052	9.037623022	0.003101027	0.027355948
RPL21P28	0.894890346	2.080793489	9.03370107	0.003107304	0.027395078
AL353759.1	0.403208214	1.347098562	9.025378259	0.003120666	0.027480329
MMP8	1.67150952	2.620503185	8.999991937	0.003161792	0.027776733
GUCY1A1	-0.43281386	2.937918729	8.998779046	0.003163771	0.027777719
NANOGP5	-0.34474742	1.191168696	8.99516025	0.003169682	0.027796822
NFE2L1-DT	0.348686527	1.588766116	8.988421381	0.00318072	0.027849812
OPHN1	-0.39716025	1.701208539	8.988044723	0.003181339	0.027849812
AL390728.6	0.325871971	3.165512514	8.958231942	0.003230659	0.028116179
BIRC3	0.420874614	7.245266214	8.946797857	0.003249783	0.02823308
VCL	-0.32194945	7.726956154	8.937727995	0.003265035	0.028283034
AC132938.3	0.345748818	1.367672225	8.920931805	0.003293475	0.028446604
SMIM26	0.338966962	3.016090412	8.91306437	0.003306884	0.028463303
AC011939.1	0.329400648	2.844524794	8.859125621	0.003400341	0.029037898
TMSB4XP8	0.355726348	2.444126781	8.838797251	0.003436264	0.029255425
KIR2DL3	-0.78871129	1.194994097	8.789943215	0.003524201	0.029782604
ZNF678	0.332904523	4.310466252	8.747341183	0.003602774	0.030274697
PCYT1B	-0.72886661	1.25503679	8.745766872	0.003605712	0.030278631
AC009716.2	-0.492556	3.380473227	8.735855491	0.003624265	0.030386654
C11orf1	0.400563525	1.451367108	8.728171776	0.003638715	0.030473473
MYL9	-0.81552075	4.312770483	8.712127367	0.003669081	0.030693231
RPL36AL	0.359112266	5.33772304	8.674556285	0.003741212	0.031034957
C10orf88B	0.364842044	1.43739199	8.640104403	0.003808637	0.031349634
F13A1	-0.52364815	7.362325174	8.63578534	0.003817178	0.031402565
IFI27	-1.05524018	2.001398096	8.61376233	0.003861034	0.03164822
COA6	0.329765948	2.358716439	8.613317604	0.003861925	0.03164822
IGLV1-44	-0.7099157	2.022468256	8.599741781	0.003889224	0.031854394
DEFA4	1.22478727	2.114306762	8.594947229	0.003898913	0.03188111
CEACAM8	1.197157468	3.200194572	8.593751221	0.003901334	0.031883386
AC027279.1	-0.48967573	1.094266435	8.589437041	0.003910078	0.031887362
GP1BA	-0.75780021	3.267657305	8.577842085	0.003933681	0.032007131
LINC01891	0.504388457	2.127146506	8.563363681	0.00396336	0.032108175
STMN1P1	0.397077883	1.302429795	8.536946625	0.004018106	0.032445716
PTCRA	-0.57504248	2.755552244	8.523986254	0.004045249	0.03250893
AL731559.1	-0.34610024	4.23401261	8.518628209	0.004056526	0.032561605
RGS18	0.365034182	6.921269041	8.51518056	0.004063799	0.032584834
RPS13	0.378944481	7.205676559	8.490030626	0.004117262	0.032848064
AC012368.2	0.424916473	1.665868981	8.462389346	0.004176857	0.033169685
BPI	0.872633488	3.189427642	8.458634169	0.004185022	0.03319909
LDLRAD3	-0.35949884	2.335560973	8.446753428	0.004210961	0.033333791
ZMAT2	0.397621175	5.483092681	8.409701844	0.004292926	0.033672741
VASH1	-0.47846945	4.881256239	8.399812302	0.00431508	0.033780654
LRRK2-DT	0.373693878	4.600547481	8.381134501	0.004357243	0.034003124
LTF	1.462465296	5.397398321	8.357712416	0.004410716	0.034276908
AC108066.2	-0.37581888	1.77687639	8.333099763	0.004467636	0.034574812
RNU4ATAC	0.97256495	1.974580433	8.312421849	0.004516042	0.034804625
DNAJC15	0.398681867	4.252531344	8.298208543	0.004549627	0.03499098
SPAG1	0.35370586	3.000108274	8.289020526	0.004571474	0.035086479
CTTN	-0.65677436	4.197619719	8.262925229	0.004634116	0.035475783
ZNF711	0.617422727	1.068716896	8.232402223	0.004708511	0.035915972

TEX30	0.349835142	1.5024359	8.227170922	0.004721385	0.035973828
FAM198B-AS1	-0.40748993	3.613100129	8.165193935	0.004876691	0.036858769
ZNF501	0.358193018	1.936999747	8.163275332	0.004881582	0.036877006
ZNF703	-0.39138789	3.912888723	8.046191939	0.005189834	0.038636871
MYO16	0.978533394	1.254653138	8.03179675	0.005229095	0.038909692
ACTBP4	-0.36298446	1.828172195	7.99281954	0.005336944	0.039504165
AL355032.1	1.587887264	1.102215993	7.975826926	0.005384679	0.039670533
AC111182.1	0.335528473	2.579082664	7.973946093	0.005389989	0.039670533
ARHGAP26-IT1	-0.37653543	3.076597513	7.948168667	0.005463318	0.040130848
CD3D	0.395009645	5.514711972	7.919308902	0.005546637	0.040562682
AC246787.1	0.488812627	1.332851884	7.896033604	0.005614789	0.040900287
PDLIM1	-0.40670573	4.935402727	7.884504932	0.005648865	0.04104805
MPIG6B	-0.73405417	5.338720614	7.855990547	0.005734066	0.041404355
PMAIP1	0.393356427	3.443885603	7.843782059	0.00577095	0.041610115
IFIT5	0.421225189	5.493496265	7.836955919	0.005791679	0.041739357
AC009086.2	0.350220054	1.266586666	7.800917621	0.0059024	0.042265628
SPARC	-0.64914272	6.134683971	7.798477927	0.005909974	0.04228473
KLLN	0.331299994	2.121850819	7.778214175	0.005973272	0.042614687
TCTE3	0.361152362	1.741497133	7.761916134	0.00602469	0.042940342
SCARNA5	0.709891562	5.863096852	7.752228568	0.006055469	0.043042289
AP001372.2	0.402163182	1.634481615	7.717082515	0.006168504	0.043486343
SH3RF1	-0.33058132	2.149097704	7.715608149	0.006173293	0.043499498
SH3BGRL2	-0.58724086	4.987186583	7.665094982	0.006339713	0.044315455
PIK3R3	-0.36876924	1.575847438	7.64945464	0.006392175	0.044598383
UGCG	0.365109174	5.159388925	7.648397581	0.006395737	0.044602324
AL078622.1	0.869446292	1.172769487	7.641398746	0.006419372	0.044725231
ZNF354A	0.392960507	3.626412358	7.633671321	0.006445572	0.044802897
SNHG6	0.322563895	4.708673265	7.582225803	0.006622839	0.045714792
PPA1	0.372892496	4.196543299	7.570810326	0.006662852	0.045948361
AC135626.1	-0.45135813	1.373227934	7.569354299	0.006667973	0.045962381
NEIL3	0.619722756	4.171306485	7.517492724	0.006853072	0.046740339
NT5C3AP2	0.359150142	3.539090351	7.514497804	0.006863922	0.046751858
MYH9	-0.33089555	10.84223942	7.452452496	0.007092738	0.04782697
AP003068.2	-0.47124028	3.666629582	7.430252534	0.007176511	0.048256334
HDGFL3	0.499201074	2.864220674	7.401017582	0.007288395	0.048658461
NCR1	-0.39372607	4.131891335	7.390473193	0.007329191	0.048783861
LINC01806	0.447288232	1.788005761	7.388446207	0.00733706	0.048783861
FAT4	-0.59125443	2.059912595	7.388092497	0.007338434	0.048783861
AP001189.1	-0.74889708	3.580925344	7.386468703	0.007344745	0.048783861
NDUFS4	0.330783983	2.748737994	7.386120891	0.007346098	0.048783861
CCR3	-0.61913035	5.251557886	7.371291972	0.007404008	0.049102692
AP002360.1	0.370635326	1.233412585	7.354119494	0.007471659	0.049467173
RPS20P14	0.57012014	1.395638018	7.334076428	0.007551429	0.049836081
SDAD1P1	0.419692318	1.202199702	7.328751711	0.007572768	0.049932627
STON2	-0.55592575	3.859024139	7.325653827	0.007585212	0.049970398

DEGs at 24 weeks post-infection relative to controls					
**external_gene_name**	**logFC**	**logCPM**	**F**	**PValue**	**FDR**
EEF1D	0.340972219	8.243789983	33.34972667	4.28E-08	6.18E-04
BTN2A3P	-0.72297803	2.650491835	29.85412861	1.89E-07	7.04E-04
RPL41	1.258716451	6.72542128	26.67746449	7.53E-07	0.001558617
IGHG1	-1.37914676	5.0785386	25.05074846	1.54E-06	0.002298463
RPL21	1.217158151	7.290731203	24.43154779	2.03E-06	0.002752844
SNRPD2	0.828088776	4.322332931	24.01266573	2.45E-06	0.002889543
B2M	0.557074162	11.39888057	23.66323982	2.87E-06	0.003050374
MYL6	0.547836449	7.420269411	23.32902838	3.33E-06	0.003135249
NEIL3	1.113736287	4.171306485	22.95825807	3.94E-06	0.003449566
RPS3A	1.475064071	7.479813137	22.51846474	4.81E-06	0.003764811
MYL12B	0.400084866	6.830898069	22.19658143	5.56E-06	0.004138051
HMGB1P6	0.585304266	2.574281538	21.61981645	7.23E-06	0.004676877
S100A8	1.465957152	8.381231209	21.46085915	7.77E-06	0.004818803
RPL9	1.151522875	6.699960312	21.22529038	8.65E-06	0.004841758
RPLP0P6	0.562950326	3.59036086	21.10653137	9.14E-06	0.004841758
AC245060.5	1.433193867	3.238469297	21.08634111	9.22E-06	0.004841758
RPL39	1.312584431	4.66441557	20.84874081	1.03E-05	0.004841758
NDUFAF8	0.3996106	3.196960897	20.82124279	1.04E-05	0.004841758
RPS20	0.809833897	7.661248579	20.68797666	1.11E-05	0.004950304
SCARNA21	0.769920693	7.179194953	20.64072077	1.13E-05	0.004950304
SKA3	1.171902071	2.845140848	20.47390333	1.22E-05	0.00505877
PFDN5	0.941136935	6.04815125	20.40947086	1.26E-05	0.00505877
RPS12	0.581445179	8.49336645	20.34544141	1.30E-05	0.00507285
NDUFS5	0.731967893	3.56772415	20.24299223	1.36E-05	0.0051814
HMGB1P5	0.625839522	3.65600296	20.15536819	1.41E-05	0.005259905
RPL7	1.272606959	7.20424667	19.79725327	1.67E-05	0.005909394
RRN3P2	-0.39144964	2.576569363	19.73843621	1.71E-05	0.005931004
MZT2B	0.392925477	5.107245339	19.6140344	1.82E-05	0.005931527
RPL7P9	1.384314574	4.556250277	19.5482438	1.87E-05	0.005931527
CBX3P2	0.436320752	2.009765399	19.45096918	1.96E-05	0.006069167
H4C2	0.659239593	4.186706134	19.22249509	2.18E-05	0.006207694
AF131215.4	-0.45723045	2.543524711	19.21307961	2.19E-05	0.006207694
KIF14	0.937159416	1.684477104	19.16422014	2.24E-05	0.006207694
RPL41P1	1.363199811	0.899833344	19.1071984	2.30E-05	0.006207694
EEF1B2	1.149377673	5.581212489	19.0769206	2.33E-05	0.006207694
RPS23	0.749223633	7.777783366	19.03480959	2.37E-05	0.006207694
RPL7P1	1.34505398	4.141883864	18.98468751	2.43E-05	0.006207694
SNHG29	0.534092863	5.837859636	18.96003208	2.46E-05	0.006207694
PTRHD1	0.490080256	3.021079558	18.95786926	2.46E-05	0.006207694
RPS3AP6	1.577996779	1.322267143	18.86190863	2.57E-05	0.00638298
ANKRD36BP2	1.382211867	5.408768845	18.79188779	2.66E-05	0.006486417
ATP5ME	0.607381528	3.554364364	18.61837007	2.88E-05	0.006809555
TOMM7	0.901580165	4.668448919	18.5772126	2.94E-05	0.006833104
RPL21P16	1.242608549	5.103807357	18.46433055	3.10E-05	0.007082619
POLE2	0.934194506	1.694761598	18.40429385	3.19E-05	0.007082619
FAU	0.483699851	7.270940606	18.33551205	3.29E-05	0.007199727
RPS7	1.135838488	6.424591687	18.24753764	3.43E-05	0.007288492
RPL27	0.902943207	7.133677371	18.24733324	3.43E-05	0.007288492
IGHG4	-1.21854945	2.714317952	18.15709748	3.58E-05	0.007495447

RPL23	1.183797926	6.902863531	18.12242615	3.63E-05	0.007495447
RPS25	0.754740972	7.092475491	18.09175912	3.69E-05	0.007495447
UBL5	0.423668722	4.984266142	17.8723811	4.09E-05	0.008006041
IFT20	0.334089925	3.28551363	17.85946635	4.11E-05	0.008006041
RPS7P1	1.390420239	3.590916232	17.77840354	4.27E-05	0.008006041
AC099336.2	1.311373017	2.200403514	17.75211419	4.32E-05	0.008006041
RPL24	0.690846604	6.541191963	17.75133366	4.33E-05	0.008006041
SF3B6	0.597051657	3.847859724	17.73897682	4.35E-05	0.008006041
TMSB10	0.382878068	8.454753283	17.73577988	4.36E-05	0.008006041
VRK1	0.64371028	4.471884514	17.66085605	4.51E-05	0.008176542
BLOC1S2	0.40948722	4.087503436	17.63906287	4.56E-05	0.008176542
AC090220.1	-0.34953619	2.032680178	17.60195718	4.64E-05	0.008193311
ZMAT2	0.58049334	5.483092681	17.58408722	4.68E-05	0.008193311
RPS9	0.365569042	8.076118135	17.52822018	4.80E-05	0.008218209
UQCRB	0.975110099	4.743208687	17.47023776	4.94E-05	0.008285048
RPL30	0.638375656	7.902191352	17.46274546	4.95E-05	0.008285048
CAMP	1.56890042	3.265588997	17.1923766	5.63E-05	0.008743983
RPS14	0.58709622	8.187313681	17.17686408	5.67E-05	0.008743983
RPS27	1.069521031	8.501661424	17.17667218	5.67E-05	0.008743983
ERH	0.487105782	3.639242711	17.17197159	5.68E-05	0.008743983
RPL32	0.524537447	8.046593947	17.15972713	5.72E-05	0.008743983
RPL24P4	0.772077929	2.84690179	17.01472732	6.12E-05	0.009201639
AC010343.1	0.637724319	2.829917259	16.9633748	6.27E-05	0.009333556
RPL23AP42	0.433139238	4.595670019	16.93197975	6.37E-05	0.00937942
RNY1	1.220134267	3.434317367	16.69056008	7.14E-05	0.009728877
IGLV1-47	-1.03943337	1.504205745	16.6779759	7.18E-05	0.009728877
RPS7P11	1.379931251	1.274870915	16.65402516	7.26E-05	0.009728877
KIF15	0.804764091	1.756419307	16.63514237	7.33E-05	0.009728877
SCARNA7	1.211339529	5.980954278	16.62862972	7.35E-05	0.009728877
AC116533.1	1.693309771	4.092635702	16.61876744	7.38E-05	0.009728877
RPS27A	0.775472325	7.541517788	16.61781515	7.39E-05	0.009728877
C12orf57	0.49617394	4.828563459	16.47537951	7.90E-05	0.010228464
RPL39P3	1.288295204	2.142135745	16.43910737	8.04E-05	0.010316556
TMA7	0.797286364	4.090165755	16.37188671	8.30E-05	0.010446948
NDUFB3	0.838519721	3.052139735	16.27490197	8.69E-05	0.010555713
RPL11	0.820304232	7.855884066	16.17235587	9.13E-05	0.010947151
RPL27A	0.472405225	8.6331405	16.1307228	9.31E-05	0.010995119
RPL26L1	0.668744783	1.508331332	16.1148712	9.38E-05	0.010995119
AC084018.2	0.403458303	1.585735292	16.05897266	9.64E-05	0.011070768
CSTA	0.869614709	4.23092559	15.92631331	1.03E-04	0.011374653
TXN	0.617079943	3.481053728	15.91502969	1.03E-04	0.011374653
RPL31	1.250357094	6.925107753	15.91495738	1.03E-04	0.011374653
RPS3AP26	1.519197195	2.893294812	15.8766671	1.05E-04	0.011374653
RPL34	1.397109291	5.993515751	15.85540733	1.06E-04	0.011374653
AIF1	0.57181017	6.037684955	15.85451982	1.06E-04	0.011374653
TPT1	0.77366111	9.742268755	15.83599991	1.07E-04	0.011393769
RASSF1-AS1	0.483069207	1.497063161	15.80208465	1.09E-04	0.011416829
MIR3609	1.07885035	3.270335753	15.76404312	1.11E-04	0.011545031
BCL2A1	0.878003462	4.511517003	15.73381823	1.13E-04	0.011631754
ATP5F1E	0.5626991	6.318026862	15.59623675	1.20E-04	0.012337639
PRDX1	0.342940227	4.992866767	15.49021095	1.26E-04	0.012839965

UFC1	0.332929965	4.507638588	15.48434051	1.27E-04	0.012839965
GMFG	0.418246724	6.905950367	15.3974502	1.32E-04	0.013201915
DDIT3	0.459629963	3.212007032	15.39061486	1.33E-04	0.013201915
SRGN	0.572029396	9.245039464	15.21097059	1.45E-04	0.014252242
RPL6	0.624252344	7.737542293	15.16555847	1.48E-04	0.014297539
TAF7	0.415727582	6.315723235	15.16546536	1.48E-04	0.014297539
H4C5	0.415430317	6.473555989	15.05863517	1.56E-04	0.014843977
NDUFA1	0.634022686	3.597163198	15.0447669	1.57E-04	0.014848201
MRPL51	0.480984123	3.439173586	14.98851414	1.61E-04	0.015159248
RPL26	1.291198416	5.314117273	14.94925349	1.64E-04	0.015273469
ZNF845	0.435609285	4.438570259	14.93722984	1.65E-04	0.015273469
RPS6	0.674263905	8.413634358	14.93388012	1.65E-04	0.015273469
COQ5	0.328003837	3.071713905	14.87172705	1.70E-04	0.01550131
ATP2C2	1.922490549	1.482673972	14.86430792	1.71E-04	0.01550131
SNORD13	0.919772778	0.728015785	14.8302771	1.74E-04	0.015571998
HMGB2	0.610413821	5.539167591	14.79778537	1.76E-04	0.015723251
RPS15A	0.847396563	6.717370465	14.78364356	1.78E-04	0.015736663
RPL3	0.433165249	9.435438186	14.66764809	1.88E-04	0.016189138
RPL35A	0.673660963	6.774043314	14.66675189	1.88E-04	0.016189138
RPL18A	0.32345825	8.863221638	14.66417547	1.88E-04	0.016189138
AC034236.1	0.958718637	2.657853232	14.61653668	1.93E-04	0.016376754
AC241520.1	-0.4993857	1.084298167	14.53465372	2.00E-04	0.01694125
RPL13A	0.441495242	9.917377292	14.47183239	2.07E-04	0.017355733
AC008038.1	0.38956854	6.480178981	14.44629459	2.09E-04	0.017355733
RPL6P27	0.696605401	4.639300323	14.44284105	2.09E-04	0.017355733
RPS18	0.770131077	8.111571246	14.40321519	2.14E-04	0.017355733
MRPL40	0.453156464	2.033264739	14.38330363	2.16E-04	0.017355733
PAPSS1	-0.40718096	5.200657165	14.35151925	2.19E-04	0.017355733
DNAJA1	0.398777096	6.08875305	14.34484199	2.20E-04	0.017355733
EEF1A1P6	0.715928872	3.146506067	14.34462228	2.20E-04	0.017355733
H2AC21	0.359963078	3.967410422	14.31462328	2.23E-04	0.017365614
AC009093.7	-0.38800619	2.921317576	14.16243174	2.40E-04	0.018406915
GSKIP	0.346684912	3.881474565	14.13042565	2.44E-04	0.018599289
RPL13AP5	0.48505372	5.408533355	14.11735244	2.45E-04	0.018622238
BX679664.3	1.350520143	2.15790025	14.06768083	2.51E-04	0.018884311
RPL15P3	0.465524461	4.141519057	13.96963317	2.64E-04	0.019413937
HAT1	0.36493271	4.073665385	13.92523316	2.69E-04	0.019643309
ATP6V1G1	0.35952491	5.862541408	13.84135637	2.80E-04	0.020362069
RPL35	0.679491959	6.993331957	13.60567679	3.15E-04	0.022087212
RPS16	0.386258892	8.442585375	13.56432248	3.21E-04	0.022168014
SELENOK	0.552871091	4.581612074	13.56430915	3.21E-04	0.022168014
IKZF2	-0.40245662	5.806633571	13.54783492	3.24E-04	0.022196384
AP001269.4	0.65812671	2.289898842	13.52468191	3.27E-04	0.022221714
CENPE	0.545368493	2.236044582	13.51739178	3.29E-04	0.022221714
RNU4ATAC	1.246170137	1.974580433	13.48384173	3.34E-04	0.022422343
RPS3	0.380087076	8.806665694	13.48047763	3.34E-04	0.022422343
SWT1	0.509514014	4.644222185	13.45505127	3.39E-04	0.022600949
RPL36AL	0.449559169	5.33772304	13.41811779	3.45E-04	0.022772146
CMTM2	0.594646727	5.149237526	13.41228904	3.46E-04	0.022772146
LTF	1.882502299	5.397398321	13.32866393	3.60E-04	0.023515133
UQCRH	0.493431073	3.992404572	13.31756207	3.62E-04	0.023540031

SNHG19	0.51696674	1.193555202	13.26634451	3.71E-04	0.023990092
EVI2A	0.61755316	5.94773969	13.25231266	3.74E-04	0.023990092
PPIAP22	0.790930975	1.844108552	13.2266758	3.79E-04	0.023992585
AC015912.3	0.64020598	1.902032673	13.20656158	3.82E-04	0.024055469
COMMD6	0.84659206	3.831179191	13.19348778	3.85E-04	0.024055469
RPL21P75	1.019463656	1.8504607	13.13680655	3.96E-04	0.024055469
ABCA13	1.282437241	3.209974603	13.13512527	3.96E-04	0.024055469
RPSA	0.460777859	8.009834343	13.11064077	4.01E-04	0.024055469
ATP5PO	0.390425266	3.697671128	13.09867602	4.03E-04	0.024055469
AC069410.1	1.153889855	1.276691725	13.09435384	4.04E-04	0.024055469
RPL10A	0.390129052	7.896890659	13.08044841	4.07E-04	0.024123684
PHF5A	0.346712395	3.460053942	12.9977234	4.24E-04	0.024631496
AP003117.1	0.609384441	1.219770015	12.98990757	4.25E-04	0.024631496
RPS27AP16	0.772414195	3.249836779	12.96852974	4.30E-04	0.024795074
EIF1AY	0.696571629	3.685986317	12.95194033	4.33E-04	0.02480573
RPL37	0.49334332	7.843532399	12.93547789	4.37E-04	0.024911427
AC115223.1	0.727068766	1.199959031	12.90755859	4.43E-04	0.025159288
RPS29	0.792239398	7.864695156	12.83443916	4.59E-04	0.025870855
SCARNA9	0.564552208	3.24112427	12.82770845	4.61E-04	0.025870855
RPS24	0.865940532	6.971741523	12.81926735	4.63E-04	0.025880877
ELOB	0.395797032	5.522817039	12.78581135	4.70E-04	0.026043624
EIF3E	0.475838779	6.630297906	12.78286733	4.71E-04	0.026043624
MRPL47	0.420484882	2.75151606	12.77388659	4.73E-04	0.026043624
AURKA	0.528673786	1.562892992	12.76870099	4.74E-04	0.026043624
COX7B	0.528442933	3.480533591	12.74793999	4.79E-04	0.026214512
HMGB1	0.327156023	6.811912093	12.70697409	4.89E-04	0.026650904
CHMP5	0.395468215	4.559798768	12.65711169	5.01E-04	0.027017045
TIMM8B	0.568848934	2.943052621	12.59566078	5.16E-04	0.027623673
ATP5MPL	0.441829993	4.196804437	12.59565234	5.16E-04	0.027623673
SS18L2	0.37057429	2.620862647	12.51440618	5.38E-04	0.028270679
RPL3P2	0.510889339	0.870293054	12.42002225	5.63E-04	0.02931843
RPL12	0.380293896	8.112423379	12.41939043	5.63E-04	0.02931843
AFF2	0.585386433	3.992384408	12.3910805	5.71E-04	0.029628001
EEF1A1	0.469904556	11.75666527	12.38109511	5.74E-04	0.02967125
SOCS1	0.449545749	3.419834847	12.31336021	5.94E-04	0.030261878
CDYL	0.469726852	5.951947475	12.26639098	6.08E-04	0.03051469
CTSG	1.470637111	0.901174976	12.25810504	6.10E-04	0.03051469
H4C3	0.566941049	5.376836215	12.25582599	6.11E-04	0.03051469
WDR89	0.442982146	3.585722224	12.23951867	6.16E-04	0.03051469
TXNDC17	0.340316755	2.886749554	12.23799373	6.16E-04	0.03051469
AC010615.1	0.584903107	1.73240342	12.22648911	6.20E-04	0.03051469
AC099560.2	1.053192207	4.064017343	12.22185922	6.21E-04	0.03051469
COX7C	0.725554628	4.747311352	12.14661935	6.45E-04	0.03138756
PPIA	0.325382948	7.665195873	12.09807279	6.61E-04	0.032022792
NRXN3	1.008559939	1.218339498	11.9912224	6.97E-04	0.033119453
SAMD9	0.440237611	7.505867782	11.94935398	7.11E-04	0.033494385
AF131215.5	-0.32257247	2.780505914	11.93011127	7.18E-04	0.03370949
RSL24D1	0.605645384	4.45901252	11.88122117	7.36E-04	0.034134849
HSP90AA2P	0.682847438	0.998704155	11.86596899	7.41E-04	0.034134849
ATP5PF	0.398717616	3.66489127	11.86302472	7.42E-04	0.034134849
KIF18A	0.49920218	1.449328685	11.78692692	7.71E-04	0.035206268

AC009086.2	0.43269444	1.266586666	11.78646294	7.71E-04	0.035206268
FO393411.1	1.222384993	0.908258257	11.76925552	7.78E-04	0.035321084
SNORD15B	1.026979311	2.825148501	11.7676353	7.78E-04	0.035321084
NDUFA6	0.415511922	3.612790595	11.74269567	7.88E-04	0.035478392
CRISP3	1.570011479	1.877184718	11.71591774	7.99E-04	0.035615824
CD52	0.522294645	7.211695234	11.69632827	8.07E-04	0.035833281
CDC42BPA	0.651703906	3.331203272	11.68320955	8.12E-04	0.035960939
AC072022.2	0.683666628	1.233929037	11.6432042	8.28E-04	0.036576341
CHI3L2	0.590415852	3.020833761	11.61950901	8.38E-04	0.036674076
HSP90AA1	0.52722224	7.524365234	11.61575342	8.40E-04	0.036674076
AF131215.2	-0.37843273	2.359346422	11.5967659	8.48E-04	0.036759364
WASH7P	0.436854806	3.084488802	11.59357701	8.49E-04	0.036759364
BDH2	0.470636873	2.225493039	11.57069397	8.59E-04	0.036936893
RPL4	0.398090606	9.213623018	11.52038971	8.81E-04	0.037766873
CDK5R1	0.58065645	4.53092925	11.4380937	9.18E-04	0.03888521
GAS6-AS1	0.676382103	4.195470397	11.40420682	9.33E-04	0.038902785
COX7A2	0.388088165	4.394235632	11.34736074	9.60E-04	0.039281461
DNTTIP2	0.332226833	4.810240264	11.33780244	9.65E-04	0.039281461
SLC2A14	-1.07133964	0.673066421	11.32411471	9.71E-04	0.039372276
SNORA73B	0.659376817	5.857224286	11.298266	9.84E-04	0.039686117
CEACAM6	1.338594238	1.533459004	11.28235221	9.92E-04	0.039765101
COX6C	0.533035116	4.026649505	11.27811996	9.94E-04	0.039765101
RPL13P12	0.66305594	5.485200543	11.2368832	1.01E-03	0.040162982
SLC2A5	0.83522667	1.737448042	11.17403903	1.05E-03	0.04122861
CNIH3	0.379893365	2.052732643	11.10701151	1.08E-03	0.042130222
NCAPG	0.697488095	1.01102547	11.09121103	1.09E-03	0.042195989
RPS21	0.689038547	6.387378066	11.05863811	1.11E-03	0.042674839
AC005912.1	0.901528451	6.665215335	11.05840378	1.11E-03	0.042674839
KNL1	0.640192583	2.854718062	11.03685739	1.12E-03	0.042694256
SLC39A1	0.324865742	1.484491702	11.0286717	1.13E-03	0.042694256
PTOV1-AS1	0.326275193	1.888116892	10.99962002	1.14E-03	0.043063155
AC024293.1	0.640800139	4.162971138	10.94461233	1.18E-03	0.044047094
AF165147.1	-0.44925017	2.268339966	10.92533457	1.19E-03	0.044364311
HAUS1	0.351401054	2.733205541	10.89070016	1.21E-03	0.044962589
LSM3	0.424578396	3.158683586	10.83343144	1.24E-03	0.045487484
AP001324.1	0.513222085	2.445947125	10.78795545	1.27E-03	0.046036112
LRRC37A15P	-0.34431756	1.761399989	10.74429655	1.30E-03	0.046509161
RPLP0	0.355295394	8.622583797	10.74048774	1.30E-03	0.046509161
OTUD6B-AS1	0.431087444	4.400315807	10.73798927	1.30E-03	0.046509161
LINC01146	-0.40456007	3.425246567	10.72368857	1.31E-03	0.046509161
MMP8	1.825780745	2.620503185	10.68887738	1.34E-03	0.04679895
RPL21P28	0.972119494	2.080793489	10.66372309	1.35E-03	0.046973181
MRPL50	0.381333522	3.436685395	10.66221422	1.35E-03	0.046973181
SNRPE	0.470129196	3.045303983	10.65831945	1.36E-03	0.046973181
METTL18	0.530091584	1.701235486	10.65300815	1.36E-03	0.04698993
ZNF593	0.373202465	1.668593484	10.62286791	1.38E-03	0.047380229
RPS8	0.469843609	8.268662313	10.60749727	1.39E-03	0.047639462
AC145285.6	0.379414358	1.238837216	10.59590732	1.40E-03	0.047809097
CROCCP2	0.364724995	6.38410252	10.58698493	1.41E-03	0.04791505
KIAA1586	0.356909749	4.016062293	10.56384792	1.42E-03	0.048336795
LRP12	-0.33900078	1.889549822	10.56058879	1.43E-03	0.048336795

DNA2	0.34877374	2.918476092	10.51608478	1.46E-03	0.048989527
HSPG2	0.603132827	2.940947508	10.49743978	1.47E-03	0.049341973

Final DEGs from GSE169687 (FDR < 0.05, fold change > 1.25)			
HMGB1P6	ANP32E	AC009086.2	PPP1R14BP3
RBPMS2	GNL2	AC064805.1	NIPSNAP3A
ERH	DPY30	MTCO2P11	PAPSS1
BLOC1S2	MT-ND4	NGDN	FHL2
RPLP0P6	ZNF493	ZNF91	RALGPS2
RPL41	LSM5	ZNF572	MPIG6B
B2M	C1GALT1C1	TMEM123	TMEM256
TMX1	RPLP0	ZNF780B	GSK3A
PRDX1	SBF2-AS1	CBX3P2	H1-3
NDUFS5	RPL27	ETAA1	ZMAT2
HMGB1P5	IER3IP1	THAP2	MRPL13
USP1	MDFIC	POU5F2	MFAP3L
TMEM70	PAIP1	DNAJC15	GP9
UFC1	EVI2A	DNM3	YAE1
RPS23	TXN	AC243919.1	AC008462.1
AC090220.1	AC005912.1	UQCRQ	YBX3P1
SS18L2	PHAX	ID3	APIP
AC099336.2	C4orf46	AP000560.1	ADAM28
SCARNA9	RPL27A	JAM3	ZNF254
MYL12B	ZNF22	IFI27	ZNF37A
ZNF765	ZBED5	EIF4BP7	ZNF204P
ZNF845	FCRL2	MCOLN2	SNAI3-AS1
DNTTIP2	SFR1	MTND5P2	AC006460.2
PHB	AC009093.2	TRIM59	LILRB2
RPL7	NANOGP4	POMGNT2	CARNS1
NDUFS3	FCRL5	CD200	RPL14P1
TMCO1	TPT1	FCRL3	SNHG6
RASGRF1	SNHG32	PAQR4	MKRN3
RPS3A	POLR3GL	ZNF614	ARHGAP42
ACAT1	SCARNA5	IKZF2	HNRNPH2
EEF1B2	MT-ND2	AL050341.2	SPHK1
AC245060.5	ZNF181	SFMBT1	ZNF860
MRPL47	RPL6P27	FAM200A	PAX5
HMGB1	HSPA13	ASF1A	PDE5A
SNRPD2	SLC2A14	ZNF510	PC
GSKIP	SACS	XRCC4	ZNF10
DUT	ZNF28	AC108134.3	CMTM2
PNPLA8	ANKRD49	LINC00494	AL158166.1
EEF1D	NSA2	FBXO5	GAS2L1
COMMD8	ATP5MPL	RBIS	FOXP1-IT1
ZNF721	CEP20	SLC9A7	EPM2A
TOMM7	SCOC	AC090114.3	RPL21P123
METTL18	EGFL7	RPS12	ATP23
SNHG29	BACH1-IT2	GFI1B	LINC02284
MRPL50	MRPL3	COL13A1	TGFB1I1
RPL39	MIR17HG	RNU7-181P	HOXB-AS1
RPA3	AC008894.2	TUBB1	CTDSPL
EEF1A1	ESCO1	SHLD3	CTTN
SGCE	CSTA	H4C5	AC079316.2
RPL3	KNL1	AC010359.3	PANK1

SF3B6	RAN	FCRLA	WASH7P
RPS7	ZNF252P	MRPS21	KIR2DL3
RSL24D1	ZNF708	GEMIN2	CSNK1G2P1
HSP90AA1	CSNK1G3	ABCE1	BTBD3
RPL7P1	RPL21P75	RASGRP3	PRKAR2B
RPL23	METAP2	CCDC26	AC108066.2
RPL6	GPATCH11	CCDC167	H2AC19
RPL26L1	AC018638.4	RPF1	AC016739.1
ACTR6	TAF9	PAK1IP1	DNAJB9
TMSB4X	IGHG1	C20orf197	GP6
RPL7P9	KRT18P31	CCNB1IP1	DUSP6
RPL9	CENPE	RPL23A	E2F3-IT1
UQCRB	RPLP1	FAM162A	RPL12
S100A8	TNFRSF14-AS1	NDUFB1	MT2A
MIR3609	NDUFAF8	RPS3	SULF2
RPL21	MZT1	SENCR	AL160272.1
ZCCHC10	AC144831.1	ZNF571	TMSB4XP8
OTUD6B-AS1	ZNF180	FAU	BLK
ATP5PF	LPAR6	BEND4	TIMP1
RPL41P1	UBE2Q2	TRMT12	H4C1
DNAJA1	ZNF43	DIPK1A	TRIM66
RPL11	CCDC59	RPL3P2	KLF7-IT1
RPS7P1	SMIM10L1	SNHG5	S100Z
RPS27	COMMD3	CD48	IRF8
RPL26	ZBTB32	ZNF302	DIXDC1
GTF2B	MRPL58	SNORA12	AL390957.1
N4BP2L2-IT2	LIG4	RPS15AP29	IFIT5
EIF3E	FIGNL1	NFIA	ELMOD2
CIAO2A	PTRHD1	STAP1	AL162578.1
IGBP1	AC034236.1	AC064799.1	ANKRD44-IT1
RPS3AP6	TAF13	GUCY1A1	TWF1
MT-ATP8	ZNF813	RPL32	ERGIC2
PHF5A	CMTR2	ACADM	TIGD7
HAT1	RARS1	OXSM	SERINC1
SNRPE	BBS12	UBAC2-AS1	LAMTOR3
ZNF92	ZNF184	NDUFAF4	AL513412.1
ATP6V1G1	RPL36AL	TCEAL8	GPR52
RDH14	ZNF761	KLF8	GMFB
TOMM20	EIF4EP2	RPS29	CTNND1
BX679664.3	IDI1	MAP3K9	Z83843.1
LINC00909	FRG1	SERPINE1	AC006141.1
AL021707.5	RPS8	EXT1	ZNF443
AP000547.3	FKBP3	ZNF675	DNAJC3-DT
CD69	HSPA8	ZNF566	ARMT1
AC125612.1	KLHL14	AC015911.4	MT-CO1
LARP7	POLR1F	TXNDC9	DIAPH1
HSPA12A	ZNF583	ZNF484	DDIT3
AC116533.1	MALAT1	CD3D	SNX16
RPL34	NDUFB2	PBDC1	NAT1
KIF20B	SVIP	MT-CO2	PIGK
AK6	ZRANB3	CCDC106	TRAPPC2B

TXNDC17	RPL5	CLEC17A	CHMP4BP1
POLR2K	MTATP6P1	KIAA1586	CARD8-AS1
DCLK2	SPAG7	MIER3	C15orf61
IFT57	ZC3H15	CISD1	HCFC1
TMA7	ZNF32	ZNF441	FPGT
WDR89	HIGD1A	MSRA	CAHM
SSB	COL7A1	AC139720.1	ADPRM
CCT2	RPS3AP47	LYRM2	GIN1
ZNF146	ZBTB6	ZNF90	PTOV1-AS1
PSMC6	H4C8	AF131215.4	AF165147.1
AC092620.1	LINC01480	RPL37	ITGB3BP
SCARNA7	GDF7	AP1S3	ZNF613
ATP5PB	PRR33	ZNF790	AF131215.2
PPIA	CDC37L1	CXCR4	AC130895.1
RPS7P11	CXCR2P1	EFCAB13	ZNF117
GPT2	ZNF432	FAM3C2P	ARSD
AC010343.1	CAPZA2	RPL3P4	DNAJB6P7
SAMD9	SMAD1	CTIF	AC241520.1
MRPS33	ARHGAP5	SNORD3C	RPS15AP10
SLU7	CEACAM8	ACTBP4	FAM200B
AC115223.1	VRK1	RPS27AP16	MSN
CAMLG	THOC7	PDGFA	ACTBP2
TIMM8B	AL121753.2	PTGS1	AC009951.4
PNRC2	SRGN	ZDHHC21	RPS5
TDRD9	BCAS2	AP000763.3	TMEM202-AS1
BTN2A3P	TSBP1-AS1	DPH5	AC009093.6
SMIM19	UGCG	LRRCC1	LXN
SELENOF	SNHG9	ABCA13	RPL13
RPL31	MTND4P14	COCH	AP001269.4
RPL13AP5	ESF1	CYB561A3	AC034111.1
RRN3P2	C12orf57	COA6	AC023157.2
PFDN2	SERPINI1	MRPL1	AL356966.1
ZNF268	KBTBD6	DNAJC10	CSF1R
AC007390.1	SBDS	SNRPD1	PGBD4
ZNF816	TPTEP1	C10orf88B	MAGEH1
MRPL51	HNRNPH1P1	AC022167.2	AC068473.5
ANKRD12	NPM1	MTCO1P11	EEF1A1P13
DNAJC8	DPM1	PIGW	GPR183
TMSB10	ZNF273	GZMA	C7orf25
RPS9	USP16	LINC01146	EIF1AY
NMD3	MTND6P11	AL158163.2	SRC
NDUFA4	RSL1D1	TSC22D1	AC092139.2
RPL13A	ZNF84	YEATS4	THNSL1
RBAK	AL355032.1	AL356273.3	CSMD2
NDUFA5	PSMD14	TAX1BP1	RTCA-AS1
RIOK2	SMIM20	OSGEPL1	LINC00653
MT-ATP6	IGLV1-44	MITF	ANKRD36BP2
COPS4	BCL2A1	HSP90AB4P	RASSF1-AS1
TRIAP1	NUP88	AC020658.5	AC072022.2
MRPS15	ZNF615	RPL8	TRMT10A
TAF7	DMAC1	AL162274.2	CLEC2B

H4C3	SLC24A3	SMOX	OCIAD2
SRP14	KTN1	EEPD1	POLR2A
PSMC2	CETN3	ACAT2	HDAC10
CHN2	RMI1	GUCY1B1	CTSA
COX7B	GAS5	FNBP1L	AC092070.2
PFDN5	HSP90AA2P	DUSP19	CHI3L2
MRFAP1L1	ADTRP	ZNF267	PITPNA-AS1
NDUFA1	PCMT1	AC008964.1	IFNGR1
AC009093.7	CWC15	ASNSD1	ZDBF2
MED7	PPIL1	TMEM184B	SLC35A1
TVP23B	WFIKKN1	EEF1A1P6	RPS21
DYNLT3	CEP57	TREML1	AC130454.1
CELSR1	AL158211.5	HMGN3	AC007342.3
LTF	NIFK	KCNN4	TTC30A
MIR155HG	BMI1	ZNF850	LLPH-DT
PEG10	CEACAM6	SLC44A5	RHOBTB1
CAPZA1	COPS2	AC018926.3	PRKACB
MMP8	ZNF616	TIMM17A	SPRED2
HAUS1	MIS12	ZNF644	IFT74
CENPQ	ZNF593	NRGN	CRIM1
MMADHC	NXT2	CCDC25	AC004837.4
RPL4	HMGB2	GCSAML	MANEA-DT
RNU4ATAC	MRPS18C	IFNLR1	AC079316.1
IGFBP4	SCARNA6	AGGF1P2	KIR3DL2
ARPC3	HYLS1	PTPRN2-AS1	CCDC152
ATP5ME	COQ5	NDUFS4	AL353708.3
PYURF	RPL13AP25	CHML	MRPL36
GON7	NFU1	LTV1	RPL13P12
ZNF271P	MYO15B	TMEM144	ARHGAP6
ANXA1	PTCRA	F13A1	CX3CR1
IGLV1-47	RPS18	ZNF527	AL354977.2
PSMA3	RPL15P3	ZNF879	BEX2
IGHG4	ZNF658	AC092652.2	S100A9
VBP1	THAP9-AS1	AC009093.8	KIF18A
RPL21P16	RPL29	PRNCR1	AC026403.1
ZNF480	EGF	MT-ND5	ZNF354C
DBI	EID1	FGFRL1	IGHV4-59
RPL35	PRXL2C	ACRBP	SIPA1L1
KRCC1	RPL10A	WDR5B	ZNF582
RPS27L	INPP5F	EIF2A	ZNF107
KBTBD8	ZNF888	LINC01237	LIPN
ZNF830	MTND1P11	RABAC1	IQGAP1
RPL24P4	NFYB	SETD9	MIR7845
NUP37	AIF1	RPL14	IL2RB
RPL24	IL10RB	ADAMTS6	ZNF85
PLRG1	GATM	CHMP2A	ZFHX3
H4C2	ZNF624	CLIC1	CXCL8
EEF1A1P5	SLIRP	SMTN	DLG5
MRPL48	H4-16	ZNF141	RPL18AP3
SMIM30	SNRPB2	RBBP8	LY96
RPS3AP26	ZNF416	ARHGEF17	RNU1-4

PPIG	ZNF14	SMG1P3	UFSP1
COX7C	TCF4	TMEM126B	AC015912.3
CLIP2	RPL10P16	SHLD2	NTSR1
THAP5	CASP8AP2	SGCB	TRAT1
MTND5P14	GIMAP7	RPS16	AC084824.1
EEF1DP7	ZNF836	RNU4-2	AL353759.1
SNRPG	RWDD1	PPM1H	NANOGP5
TOMM22	MTERF3	MYL9	NFE2L1-DT
SRP9	ARV1	IL7	OPHN1
RANBP6	SRFBP1	AC138035.1	AL390728.6
NDUFB3	MT-ND3	AL078622.1	VCL
RPL30	C1orf198	SH3BGRL3	AC132938.3
CD52	CCDC82	SOD1	SMIM26
COPDA1	FBXO22	SDK2	AC011939.1
ENY2	SMC6	ZFAND1	ZNF678
MYL6	PPIAP22	BAIAP2	PCYT1B
MDH1	RPS14	C8orf37	AC009716.2
SNORD13	POLE4	ZNF17	C11orf1
OBI1	RNF216-IT1	CD19	AC027279.1
TRMT10C	BPI	UGT8	GP1BA
AC010615.1	CAP2P1	ABAT	LINC01891
AC099560.2	KCNAB3	AC040904.1	STMN1P1
ZNF208	BAIAP2-DT	CCDC18	AL731559.1
MRPL40	SPIN4	SGO2	RGS18
NDUFA6	RPL21P28	ZFP14	RPS13
COMMD6	LIMD1-AS1	MIR4432HG	AC012368.2
ZNF525	BACH1-IT1	CCDC65	LDLRAD3
SUB1	ZNF226	PARD3	VASH1
AC011676.1	BIRC3	GMFG	LRRK2-DT
UFM1	WNT10A	NRXN2	SPAG1
RPS24	MIR6124	SDK1	ZNF711
SCARNA21	UBL5	AC006364.1	TEX30
RPSA	BCL2	MTCYBP3	FAM198B-AS1
GPM6A	GEMIN6	AP003068.2	ZNF501
LSM3	TBCA	RN7SL288P	ZNF703
NSMCE1	ATP5F1E	ZNF680	MYO16
CRISP3	RPL22	PMAIP1	AC111182.1
RPS25	DAB2	FTH1	AC246787.1
XRRA1	ZNF138	RTL5	PDLIM1
CAMP	AC087893.2	NUDT15	SPARC
LRRC37A15P	PTGER2	ZFP82	KLLN
ZNF420	CTSG	AC005041.3	TCTE3
DEK	MAIP1	AC003681.1	AP001372.2
EIF4A2	RPS27AP12	RCN2	SH3RF1
RPL4P4	SNHG8	COL6A3	PIK3R3
RPL23AP42	PIN4	AC010201.2	ZNF354A
MT-ND1	ADH5	KPNA5	PPA1
CHMP5	SPDL1	TMEM19	AC135626.1
CBX3	MAGOH	DEFA4	NEIL3
C11orf80	SNHG19	AQP10	NT5C3AP2
IFT20	AC122718.2	AC022167.1	MYH9

AC087284.1	ZBTB1	BNIPL	NCR1
CD24	RPS15A	TRAPPC2L	LINC01806
JRKL	PLEKHF2	KIF15	FAT4
RPS27A	HDGFL3	LINC02631	AP001189.1
BRIX1	FBL	PPP1R12B	AP002360.1
AC008038.1	ATAD5	TNFSF4	RPS20P14
RPL35A	NOC3L	LRRC40	SDAD1P1
ZNF781	ZNF570	FUNDC1	STON2
FO393411.1	ARHGAP32	SH3BGRL2	SKA3
RPS6	EEF1E1	NME1	MZT2B
ATG5	SNX2	RPS27AP5	KIF14
PFDN4	MRPL32	SLC38A5	POLE2
COX6C	ABLIM3	TPM3P7	RNY1
BDH2	MTCO1P12	CRIP1	AC084018.2
UQCRH	LSM7	ARHGAP26-IT1	ATP2C2
SNORD3A	AGPAT5	AL391832.2	RPL18A
SNX4	ZNF737	BX571818.1	H2AC21
CEBPZ	ZNF253	GPR18	SELENOK
ATP6V1D	LSM1	CCR3	SWT1
NUDCD1	COX7A2	AC122718.1	AC069410.1
RPS20	AC005730.3	IGLC2	ELOB
RPL22L1	MT-CYB	AL844908.1	AURKA
GIMAP2	SKA2	VIL1	AFF2
MT-ND4L	ZFP30	GLO1	SOCS1
ZNF594	ZNF569	AC006017.1	CDYL
PSMD10	MTND6P5	RPL19	NRXN3
SBDSP1	ATP5MD	RPS4X	AF131215.5
SMIM15	SLC30A4	AL669831.1	CDC42BPA
RPF2	AP003117.1	FAM3C	CDK5R1
GNL3	ZWILCH	ALOX12	GAS6-AS1
THAP12	CHCHD1	SGPP1	SNORA73B
TMEM126A	TCF7L2	ITGA1	SLC2A5
HINT1	MTATP6P11	AC024293.1	CNIH3
MRPL15	RECQL	SGSM1	NCAPG
SNHG12	BTLA	KRT8P46	SLC39A1
PSMA4	DDX50	TCF19	AP001324.1
OSTC	RPL39P3	E2F5	AC145285.6
ZNF260	HSP90B2P	CMTM5	CROCCP2
ATP5F1C	MTERF1	METTL8	LRP12
SNORD15B	MRPL11	SAMD14	DNA2
VAMP8	RPL15	ZNF567	HSPG2
CASP3	CTBP2P8	CLEC18A	
ATP5PO	LSM8	MT-ND6	

COVID-19 related genes form database		
**CTD**	**GeneCards**	**DisGeNET**
CCL2	ACE2	S
IL6	TLR7	IL6
TNF	IL6	ORF1ab
CXCL8	TMPRSS2	ACE
IL10	CRP	REN
IL1B	TNNI3	TNF
IL2	TNF	TMPRSS2
AGT	IL10	CD4
CCL3	NLRP3	CD8A
CXCL10	F3	AGT
IL7	DPP4	ALB
UGCG	HLA-A	IL1B
MUC1	IL4	GPT
CCR2	ACE	F2
CSF3	TLR4	IL10
BTK	APOE	LOC102724971
CRP	IFNA2	LOC102723407
LZTFL1	NRP1	INS
TMPRSS2	ABO	IFNG
PLSCR1	HLA-C	N
IL2RA	IL2RA	CXCL8
CD209	AR	AMH
TMPRSS4	NPPB	IL1A
IFNAR2	IL1B	IFNA1
TMEM106B	LOC117134593	FURIN
ICAM5	EGFR	CSF2
IL10RB	OAS1	DPP4
ATP11A	SLC6A20	TNNI3
BCL11A	LOC117135106	E
SELE	LOC117134608	F3
ABO	LOC117134604	IL2
F8	LOC112679198	GOT1
FUT2	IL22	CRX
HCK	ARNTL	IFNB1
TYK2	F2	NFKB1
ACE2	ADAM17	AGTR1
BSG	IFNG	RPGR
TP53	CCR9	PDB1
NFKBIA	IFNB1	IL6R
RELA	IFNA1	PLAT
NFKB1	CD99	CALCA
CASP8	INS	CCL2
ABCB1	CD8A	IL17A
CASP3	CCL17	SERPINA13P
PCNA	CSF2	HLA-C
AKT1	FCGR3A	LSAMP
NOS2	TNFRSF1B	LAMP3
BAX	TAMM41	ABO
CDKN1A	TLR3	HBA1
BBC3	CCR5	CENPJ
ICAM1	MUC1	IL2RA
SOD2	CXCL10	NELFCD
IL1A	PMS2	MB
SERPINE1	CHAT	CXCL10
GPT	INPP5E	VWF
FOS	CXCL8	CTSL

TGFB1	HLA-B	NLRP3
COL1A1	IRF7	ORF8
HSPA5	CXCR6	CTRL
PPARG	FURIN	CTSB
CCND1	CD4	IL4
CYP3A4	FCRL5	SPECC1
ESR1	IFNAR2	MAS1
BECN1	BSG	ZFYVE9
CTSL	TNFRSF1A	MS4A1
STAT3	TLR2	PDCD1
NR1H4	ALB	RTN1
MCL1	IRF3	RTN4
SRC	ADM	BSG
CPT1A	VWF	MTOR
MAP1LC3B	LY6D	NCKIPSD
CDH1	C11orf71	AHI1
HMOX1	CCL2	SOAT1
BCL2	IL17A	VEGFA
BCL2L1	ADAMTS13	PLG
PARP1	IFITM3	KRT20
STAT1	S100A8	F8
ABCC2	HSPA5	JAK1
MYC	SERPINE1	SERPINA5
CD36	NRP2	CD19
SQSTM1	LOC119086083	IFNA2
VEGFA	SERPINA1	PSMD1
GLB1	DDX58	STAT3
IFNG	PLAUR	TTR
NOS3	CALCA	KNG1
CS	IL7	NPPB
HIF1A	HIF1A	EMSLR
ALB	HMOX1	AGTR2
RUNX2	MAVS	CSF3
CTSK	CTSL	IL7
MT2A	IL18	IL18
MAP2K1	TBK1	PROS1
PPARA	FYCO1	LINC01672
CYP27A1	IFIH1	ESR1
TLR4	RAB7A	NFE2L2
LEP	LZTFL1	PSS
MYH7	SFTPD	ORF3a
RB1	TGFB1	C5
MMP9	TREM1	EEF1A2
FABP5	STAT1	ERBB2
MAP2	F5	G6PD
CREB1	S100A9	GGT1
CASP7	LOC117134611	NCAM1
MAPK8	LOC117135104	ADAM17
MMP2	LOC117135105	TLR4
CAT	LOC117134605	GGTLC5P
EDN1	LOC117134606	GGTLC3
INS1	LOC117134607	GGT2
CASP9	EDN1	GGTLC4P
PTGS2	MUC5AC	FCGR3A
DDIT3	MBL2	FCGR3B
APP	HMGB1	HSPA5
CTNNB1	CD209	IL1RN
ACTA2	AGT	INSRR

CASP12	PRF1	JAK2
CYP2D6	SDC1	CCL3
ABCB11	FAS	CCL5
FAS	IL13	TLR3
CCNA2	CCL5	KLK4
MKI67	ELANE	SH2D3C
HSF1	AAK1	BTK
CYP2C9	ANGPT2	VPS51
EIF2S1	PTX3	ABCB1
ABCB1A	CD14	WDTC1
IL4	TLR8	PGR-AS1
CYP3A23-3A1	OAS3	C3
RAF1	AVP	CD14
NFE2L2	IL10RB	F10
TIMP2	SLC6A19	HIF1A
BAK1	XCR1	HLA-A
CTSB	CCL3	SERPINF2
CCND2	SIGLEC1	SLC5A2
SPIRE1	ZC3HAV1	ADAMTS2
ACOX1	JAK1	TLR7
NCF2	SFTPB	STS
EIF4H	VPS39	CYP3A4
MAPK3	AGTR2	FLT4
JUN	SELP	HMGB1
EGFR	LOC118966792	LTF
CADPS	MYD88	EPCAM
MAPK1	NFE2L2	COX2
RO60	CCR1	PRF1
MAPT	ITM2B	MAPK1
BDNF	LOC117600004	PKD2L1
SCD1	CLEC4M	SLC33A1
CXCL1	SERPING1	ADAMTS13
MTOR	PDCD1	IGKV2D-29
EEF1A1	IFNL1	PYCARD
COL3A1	CD163	ASZ1
COL1A2	SERPINC1	M
CD74	APOL1	ORF6
TRIB3	FLT1	ORF7a
MTTP	IFNAR1	AR
CEBPA	ALOX5	CASP1
MT1M	ADIPOQ	CDSN
CYP7A1	GPT	CEL
GSR	IL2	HLA-B
VIM	EPO	IGHE
PENK	PF4	IL13
NR3C1	ERVW-1	LCT
CYP1B1	LPA	MME
FASLG	MPO	MUC1
FASN	KNG1	OCA2
HAVCR1	IL33	PRKAA2
BIRC5	RAB1B	STAT1
RCN2	ARF5	B3GALNT1
MIR379	TLR9	RHOD
VDAC1	ICOSLG	SCPEP1
CYP2E1	CCL4	FUZ
MYLK	STAT3	SCAI
GJA1	TTR	ORF10
CYP1A2	ERG	MS2

PRMT5	TAS2R1	AKT1
ATF4	VAMP8	C5AR1
FABP4	VDR	CASP3
IGF1	IL1A	CD48
MIR151	CXCL1	CD68
GSK3B	CXCL2	EGFR
ABCB1B	VPS41	DMTN
CSE1L	LINC02967	EPHA3
KRT19	G6PD	EPO
NR1H3	SCGB1A1	ICAM1
POLR2A	MTHFR	JUN
MYH11	IL6R	MBL2
MBP	ENPEP	MEFV
PPT1	REN	MPO
ATP1A3	CCR6	PRKAA1
PPM1B	F11	PRKAB1
LMNA	HAVCR2	NECTIN1
MPST	BECN1	SELP
BGLAP	AGER	TFPI
HK2	TNNT2	APOL1
CCNE1	SNAP29	RNMT
IGF2	GSTT1	HGS
CEBPB	STX17	ISG15
REN	SAA1	CABIN1
PDGFB	TAS2R38	CD2AP
RHEB	SIGMAR1	CD274
SNAI1	AKAP8L	IL23A
STAT4	NEU1	CDCA7L
MAL	RAB8A	OTOR
OPTN	VPS11	IFIH1
NEDD4	ESM1	SLTM
DYNC1H1	MMP1	OMA1
ACADS	UNC93B1	SLC6A19
BNIP3	LDHA	SERPINA3
SLC39A10	GDF15	ACR
CYP2B10	MIR146A	PARP1
MAP1LC3A	AP3B1	AGER
NCF1	DNMT1	ANGPT1
NPPB	HLA-DRB1	ANPEP
MYH10	APLP2	AQP4
HSPA1B	NME7	CD38
CCL9	RAB8B	CDKN3
PTPRD	CALML3	CCR5
FOXO1	RAB4B	CRYGC
GLUL	NME5	CYP2D6
TNFAIP3	CMPK2	ATN1
TYMS	TTYH1	ELANE
CCL5	ZNF366	FBL
OCLN	PYDC1	GLP1R
AR	BTBD17	HLA-DRB1
CXCL2	VKORC1	HMOX1
HGF	STING1	MET
CYP4A10	NCAM1	NR3C2
HMGCR	CXCL16	MMP9
MIR31	LOC111832671	MUC5AC
TJP1	TH2-LCR	MX1
NDRG1	LOC117152611	NEU1
ABCB4	LOC117152610	OXT

CYB5R3	LOC106699567	SERPINE1
HSPA1L	LOC116158494	PIK3CA
POSTN	LOC116158495	PIK3CB
ALDH1B1	LOC117204000	PIK3CD
ELK1	LOC117204001	PIK3CG
LAMB1	LOC119230225	PPARG
NQO2	LOC112590816	PROC
ACTB	LOC112590817	MOK
ACADL	LOC119266102	SAA1
ITGAM	LOC116183086	NRP1
MIR10B	LOC116183087	NAPSA
MT1	LOC117693187	SH2D3A
CYBB	ICAM1	IFITM3
CDKN1B	SURF1	PITRM1
FN1	IFI44	COPE
SREBF1	AHR	DDX58
EIF4EBP1	SLC6A15	PLA2G15
INTS3	NEDD1	NAAA
TOP1	CST3	BBS9
MYH6	IRF5	FAM3B
GEM	BRD4	ACSS2
KDR	AKAP8	ACCS
AHCY	RPL36	CRYGEP
GADD45A	FBXL12	A1BG
CTSD	TIMM29	KLK3
PTK2B	WWP2	FAS
PRKCA	LGALS3	CD40LG
HNRNPA1	OAS2	CECR
ERGIC3	TEK	CPOX
ITGAV	KLF2	CSF1
PRPSAP2	SDCBP	SLC25A10
CLNS1A	EIF2B1	DAPK3
H2AX	AXL	DHODH
HSP90AA1	SIRT3	DIO3
PER3	STX7	EPHA2
TARDBP	STX6	FUT3
RGS18	VPS16	GCG
GPX1	VPS18	NR3C1
HK1	HSD17B4	HBB
ZMPSTE24	TPM4	HBG2
ACACB	DOCK6	CFI
CAMK2A	KDELR1	IGF1
NFATC1	LRRFIP2	ISG20
CYP1A1	EIF3G	ITGAM
HNF1BB	SURF6	IVD
SUR-7	MON2	KLRC1
LAMC2	TTC27	LEP
COG1	LMNB1	LRP2
U2AF2	CNBP	MMP8
ABCC4	GZMA	NOS2
ANGPTL6	TMPRSS4	NPC1
PDIA6	ISG15	MAPK8
APOA1	HLA-G	PTGS2
GOLGA3	IFNL4	PTPRC
VIPR2	PDHX	TF
ERCC1	HMGCR	UMOD
LRP1	CYB5A	AD5
SLC18A2	FAT1	SKAP2

CAPN1	PEG10	PPIG
TPM4	PDS5A	RAPGEF5
PLPBP	MLEC	CTPP
EGR1	PALM	CLEC4M
PINK1	PLA2G7	CPQ
PRKN	AKT1	SLC27A5
CYP8B1	PLG	SPACA9
MARCKS	VEGFA	MCF2L
MMP1	TRPV1	SIRT1
CCNB1	TRPA1	SETD2
RNY5	PAFAH1B1	FEV
PRL	GSTM1	PARP9
SLC7A11	C9orf72	ZGPAT
AANAT2	WWP1	GGTLC1
ARHGAP42A	RBM15	COPD
CDF-1	IFNL2	ORF7b
RAB7A	PLA2G2A	LOC102724197
SFN	CHRFAM7A	ABL2
NOB1	MIR139	AHR
PRLR	MIR30A	AMBP
SOD1	SNHG16	ANG
RPL35	SNHG6	APEX1
ACTA1	MAS1	APP
GATA4	PTBP1	ARSL
VPS45	ILF3	ASIP
KIF20A	TICAM1	AVP
MBOAT7	SMN1	OPN1SW
PRDX6	S100A12	BCS1L
TFRC	MADCAM1	BRCA1
ARID3A	ITGAV	CAD
MIR29C	C5	CALR
PDCD4	GC	CFTR
UCHL1	C5AR1	CHAT
PGD	RB1CC1	COL11A2
RNY3	VPS33A	CRYGD
FBXO32	FCGR1A	DNAH8
FTL	FTL	EPHB2
GAK	MAF	FLOT2
MTMR6	MAFB	FLT1
IGSF11	UNC13D	FN1
DEF6	TREM2	GAST
AQP1	FABP2	GAD2
CAMSAP2	THBS3	OPN1MW
FKBP3	LAG3	GFAP
SCFD2	MIR126	GH1
MFF	MIR29A	CXCR3
G6PD	MIR10B	CXCL1
SCHIP1	MIR31	GZMB
ARPC5L	CD274	HOXB5
ATF2	NOS3	IL5
SIN3A	IL2RB	ITGAX
ART3	CNR2	KRT12
ATP1A1	ENPP2	LPO
CSF2	P2RY14	CXCL9
SLC30A2	SREBF2	MRC1
LARP4B	IL37	NT5E
DAP3	RIOX2	OAS1
CXCL5	MDM2	PAEP

IL12B	GPHN	PCOS1
COL4A1	MYO1E	PF4
SCCPDH	TCF3	SERPINA1
PTEN	ACAD9	PKM
LPL	CCHCR1	PON1
ENO2	DHX37	PPARA
IL12RB2	COPG1	PRH1
TSC22D1	QTRT1	PRH2
SEMA4B	SLC35F1	PLAAT4
GATAD2A	PSME3	BRD2
ITPR1	FNDC5	RPS6KA1
RBM15	PALS1	RTN2
EHHADH	MMP3	S100A8
NPPC	LCN2	CCL7
SNU13	ELAVL2	CCL20
ATP2C1	PTGDR2	SECTM1
BRD8	TNFSF14	SLC3A2
ME2	DEFA1	SMARCB1
PTGES2	UBE2L6	SRY
GCLC	KLRC2	TBXT
NDC1	MIR152	TLR2
CKB	ITK	TWIST1
S100A4	BTK	USF2
ALPI	CAT	VDR
CSF1	BCL2	CXCR4
PFKFB2	F10	BCAR3
MAP2K2	SLC6A4	NR1I2
CSNK2B	PIK3C3	CD163
EIF4G3	MIF	ABCB6
MYEF2	LEP	SPAG5
WDR26	NEDD4	CXCR6
CELSR3	SMURF1	FASTK
ACER2	ULK1	PRAME
APEX1	OLR1	ARIH1
RGS19	PER3	IL37
EHD3	SELL	IGKV7-3
UPF1	TIRAP	CD209
YPEL1	SMURF2	IL22
ATP6V1C1	ATG13	TLR9
MCM2	FBXW7-AS1	SAGE1
ETF1	PLAU	CCDC88A
ATG5	PIK3R1	NKRF
PSMD3	EPHA7	TMPRSS4
FGF1	ACACA	CD177
EPN2	NRG1	HAMP
DDX6	SLC19A1	PAGR1
COX8A	VCL	IL1F10
RAC1	PTPRS	HAVCR2
ABCF2	OSMR	UBXN11
DHCR7	ATP6V0A2	DNER
IRF4	LIAS	CARD16
BAZ1B	SGCE	MRGPRD
GLG1	LMNB2	SYNPR
OAS1	RPL13A	PIKFYVE
BID	CDC37	PCSK9
AURKB	ATP6V1D	SLC26A5
RAB1A	POLD3	SMIM10L2A
PKIA	NUCB1	NPS

LAT2	PSMD11	SPANXB1
PLA2G4A	SIN3B	ACOD1
AP1G1	RNF213	ERICD
INTS6	TRA2A	ADM
GRIN2B	ANKLE2	ADRA1A
SLC2A2	GCNT3	ADRA2B
EGF	SNRNP70	AKR1B1
IMPA1	SAFB	ANXA13
GCNT1	SACM1L	APC
EDNRB	TRAPPC9	APOA1
TPP2	SYT4	APOE
EHD1	DAZAP1	ATR
EIF4G2	NCAPH2	BCHE
SESN2	PLXNB2	BCR
AHSA1	RMND1	BRAF
MICAL1	SAFB2	BRS3
SLC12A5	STX18	TSPO
ANO1	TUT1	CA2
CNBP	TBC1D5	CAMP
NR4A2	PCBP3	CAV1
ABCC1	PIH1D1	CD74
TRPV2	TBC1D8	CEACAM5
NOX2	NTPCR	CTSC
GPX4	ECHDC3	CPE
LUC7L	RAVER1	CST3
RBMX	WDR18	CTLA4
TOMM40	KRI1	DDIT3
PDGFRB	MYDGF	DNASE1
PTK2	PPAN	EDNRA
FLT1	GRWD1	EGF
PPP2R1B	STEAP1B	ENPEP
WNK1	ANXA2	ERG
CASP1	SOCS1	F7
NUP153	NPR3	FOLH1
KIF1A	PAPPA	FUT1
RNY4	XK	GALNS
PZP	DELEC1	GC
FOXO4	ANXA5	GOLGB1
PTPRN	SMPD1	GPR42
TIMP3	SPP1	HK1
AKT1S1	P2RX7	HP
TRP53	F2RL1	HPN
COLEC12	F2RL3	IFIT1
CALR	CHI3L1	IGF1R
CHUK	THPO	IL1R1
KRT8	BDKRB1	IRF1
CDK2	CPA3	IRF7
NOTCH1	LGALS9	ITGA2B
PDX1	NEAT1	ITGB2
AOC1	MALAT1	ITGB3
MPO	CTLA4	KLKB1
ANXA2	FPR1	KRT10
CSNK1D	SIRT1	KRT18
DNM2	ATF4	LY6E
GNG2	IDO1	MAX
PEA15	TNFRSF13C	MNT
PPM1A	SOD3	MOG
SEC23B	CXCL3	ABCC1

PRKCB	ATG14	NEFL
TGM2	OR51E2	OAS2
AGXT	MIR1207	OPRD1
COX6A2	ANPEP	PDE4A
SPAG9	CXCL5	PGF
APRT	MCUB	SLC25A3
MTF1	ITGB3	PIP
ARL4C	SOCS3	POMC
MIR34A	CCL20	PPIA
MLEC	PSMD1	MAPK3
TRPV6	SLCO4C1	RAF1
GPX2	UBC	REL
MTNB	RELA	RPS6KB1
CYP3A11	KLRC1	S100A12
SPTLC2	ITGAM	CCL4
LONP1	IL7R	CCL8
MIR155	NLRP12	CCL11
RNY1	IRF9	SELE
ID1	IGHMBP2	SELENOP
PDP1	TK2	SLC6A1
APOB	TRIP4	SOD1
SYT1	TOMM70	TRIM21
IL17A	G3BP1	SSTR4
EIF3C	G3BP2	TAPBP
MMP13	CXCR4	TRBV20OR9-2
SPP1	CPT2	TGFB1
SYN2	MAPT	THAS
WIPF1	GAA	THBD
E2F1	B2M	TLE1
NOX4	JUN	TLE2
TOMM20	HADHA	TLE3
ADM	FHL1	TLE4
DRD4	CD81	TLR5
MRPS6	ACADVL	SGCE
IL4R	APEX1	LPAR2
RPS20	EIF2S1	TMPRSS11D
STK39	ITIH4	LITAF
GSDME	DHPS	CYTIP

ME/CFS related genes form database		
**CTD**	**GeneCards**	**DisGeNET**
RYR2	ATM	FURIN
ADRB3	TP53	NR3C1
NTF3	BRCA2	RNASEL
ITGA2	FBN1	TNF
OPRM1	BRCA1	ETV6
CRH	SCN5A	NTRK3
ITGB1	NLRP3	SLC6A4
CALCA	PTEN	NHS
HSPA8	WT1	TGFB1
ABCC1	FLNA	COMT
INS	NF1	TRPM3
SERPINE1	TNF	IL10
NFKB1	IL6	APRT
CCND1	MSH2	EIF2AK2
NOS2	PTPN11	NOS2
RELA	APC	HLA-DRB1
CAT	KCNQ1	PTGS2
PTGS2	MSH6	SERPINA6
MAPK3	IL10	HTR2A
MAPK1	MLH1	TPH2
IL1B	PMS2	MFAP1
IL6	CEP290	NFE2L2
TNF	MECP2	PF4
FGF	COL4A5	MIR126
DOP1	KRAS	MIR143
PRL8A2	TSC2	OAS2
SYPL	USH2A	MIR150
ZFP131	PTCH1	NPAS2
CALY	BRAF	MIR330
GNG3	CYBB	MIR30C1
NSMCE3	BLM	RNR2
P2RY12	STK11	MUC1
TRP53BP2	KCNH2	MIR30C2
CAR6	C11orf65	MYOG
CER1	ABL1	MBL2
GNG4	ALMS1	MIR99B
SIAH1A	FGFR2	PLAU
SULT1A3	MKS1	KRT20
ADRA2B	SMAD4	CD14
GNB4	COL4A4	SIGLEC12
SLC29A4	STAT3	TRPA1
FRS3	SMARCA4	MTA2
TRMT10B	SETBP1	CD6
GRK2	DICER1	CD9
OPRL1	WRN	MS4A1
PARD6G	PDGFRB	ASIC3
ZFP638	COL4A3	ADIPOQ
GNB2	NBN	GDF15
MYL2	HLA-DRB1	CD38
GNG12	JAK2	VIP
ADRA2C	PIK3CA	TRPM2
KHDRBS2	HRAS	ATR
SCT	FGFR1	AVP
MEIS1	RECQL4	PRKCD
ARHGAP39	BCR	BLM
CRPPA	IL1B	FANCM

GNG2	NSD1	REN
HSPB6	RET	ACTB
ZSCAN21	ERCC6	CFB
ADGRB3	ATRX	SULT1E1
FH	CTLA4	TRBV20OR9-2
MBL1	PALB2	TH
ADRA1D	RUNX1	CD69
AK2	ALB	ERVK-18
NAP1L2	IFNG	CPT2
AOC1	CHEK2	NRSN1
CELA2A	CYBA	CTSB
LSM1	POLG	DBI
MTX2	KIT	ACE
SLC4A3	VHL	DUT
UNCX	NRAS	EMX2OS
RNASEH1	INS	ELANE
RPE	CFH	ESR2
SCAF1	CDH23	PTK2B
THOC6	CDH1	FCN1
KRT2	NPHS2	EMB
PPP2R1A	ASXL1	MAP3K8
DBH	NPHS1	CD24
DLAT	COL3A1	MASP2
GRK5	TSC1	HPSE
MEX3D	PDGFRA	TMED2
OLIG3	NOTCH1	TPPP
RANBP6	OFD1	PSIP1
SUCLG2	BBS1	CHP1
UBL5	CDKN2A	IL17F
AGAP3	LMNA	NLRP3
AQP7	SERPINA1	H3P19
ETFB	NOD2	ADCYAP1
IPP	BBS10	RBM45
NEUROG2	TMEM67	FLNA
NT5DC1	FGFR3	GABPA
C1QBP	TGFB1	COPD
CSK	BRIP1	IFNA1
RDH14	CACNA1C	IFNA13
ADCY7	SDHD	IFNB1
NGLY1	AKT1	IGHG3
TCF15	TET2	IL1A
TUT4	NCF1	IL1B
AKAP10	AIRE	IL2
CASKIN1	CRP	IL6
SNX2	SDHB	IL18
EIF1A	CC2D2A	KIR3DS1
FBXL3	LRRC56	KLRC2
HOXB7	HLA-B	HTR7
RAP1A	FAS	HTR1A
RBP2	IFIH1	HLA-DQB1
YME1L1	COL5A1	GCH1
ZFP503	BBS2	GDNF
ARMC8	MYO7A	AMPD1
B4GALT7	RPGRIP1L	B3GAT1
CMPK1	CUBN	DISC1
KCTD3	RAD50	SESN1
OPRD1	TGFBR2	REM1
PLCL1	CREBBP	GRIK2

VARS2	MEN1	HTT
ATP5F1A	CBL	CFH
EPHA4	BMPR1A	HLA-DQA1
MESP1	GNAS	LTA
RBM34	CCND1	
AGTR2	TNFRSF1A	
CNOT7	KMT2D	
TCEAL1	ACE	
ADRA1A	HFE	
ATPAF1	CXCL8	
CYP2C	MIR17	
ECHS1	RAF1	
MRPS18C	GLI3	
PAFAH1B2	CD40LG	
RNF115	TLR4	
UBR1	TBX1	
AMN1	COL1A2	
INPP5F	WAS	
RIOK2	SDHA	
SART3	JAG1	
HOOK3	SLC2A1	
KLHL7	BBS4	
LDB2	GATA3	
MTF2	COL1A1	
SLC5A1	TGFBR1	
SULT1D1	TERT	
ADRA2A	UGT1A1	
ARFIP1	LAMB2	
CBLL1	WFS1	
FNDC3A	BBS12	
GNL2	BDNF	
HOXA2	MKKS	
LIPE	KCNE1	
NCAN	NPHP1	
NELFA	STAT1	
PHB1	RAPSN	
RSRC1	EVC	
CYP2C37	SLC26A4	
EBPL	HNF1B	
GNPDA2	GJB2	
KLHL2	VEGFA	
KRIT1	MET	
PPIL4	CDKN1C	
SMARCA1	INSR	
SMC5	IFT140	
AGTR1	CFTR	
CDX2	CHAT	
CLCN6	MMP1	
HELZ	MT-ATP6	
ADRA1B	BBS9	
SENP7	C3	
VASP	FOXP3	
OPRK1	UBE3A	
P2RX7	BBS7	
SH3YL1	AGRN	
WNT4	IL4	
DMTN	SLC12A3	
GCG	CIITA	

ITGAV	MIR34A	
ACTN1	COMT	
DAP3	AR	
PHF14	WDR19	
THY1	MIR223	
UBE2D2	HSPG2	
WNT3	AXIN2	
ACO2	TREX1	
MAPRE3	TP63	
PARG	CHRNE	
SCN3A	EP300	
TERF1	ANK2	
AAAS	MITF	
FMO4	CDKL5	
G6PDX	MUTYH	
PTBP2	LMX1B	
RBM12	PCDH15	
TAF9B	COL5A2	
WNT1	THBD	
DES	LIG4	
FGF5	BAP1	
POU3F1	DSP	
CHRNA7	RRM2B	
CPSF6	SOS1	
CYP3A13	COL2A1	
SLC22A3	IGF1	
CAPN5	FMR1	
DAPK2	AHI1	
FABP3	TBX5	
ITGB3BP	NR3C1	
MAP3K4	SCN1A	
PRMT3	MT-TL1	
RAI1	SDCCAG8	
SMARCA5	USH1C	
HADH	SAMHD1	
HOXB9	SCN4A	
MGAT2	TCF4	
PRDX6	PLOD1	
SCN1A	TGFB2	
SIPA1L1	LEP	
CTSK	POT1	
EMX2	DOCK8	
FMOD	HCN4	
HEXIM1	PRKAR1A	
KCND2	IL2	
SCN1B	IFNA1	
XRCC4	CTNNB1	
ADRB1	BARD1	
CEP57	MT-CYB	
CUL4A	CLCNKB	
IL6RA	MEFV	
LTBP4	KMT2A	
NEFH	PTPN22	
RPL28	MIR21	
FGF12	F5	
FOXC2	GATA2	
HDAC7	NRXN1	
LAP3	FLCN	

LRBA	INPP5E	
LYPLA1	ATP7A	
PIP4K2A	KCNJ2	
CCNC	BBS5	
EYA1	TTC21B	
NUP153	NIPBL	
SMC3	MAPT	
TFAM	LPIN2	
TLR9	MT-ND1	
EOMES	MAP2K1	
PNN	ERCC8	
PRKCE	BCS1L	
PRSS12	ELN	
VDAC1	CHRND	
FGF8	EGFR	
FLRT2	ADGRV1	
IFNGR2	CASP8	
MEOX1	PHOX2B	
MYO1E	RAD51C	
PAX3	PKD1	
ZAP70	MIR20A	
GPM6A	POLD1	
KYAT1	SCN10A	
PCYOX1	LYST	
SP3	OCRL	
ST13	IRF6	
CA2	SMC1A	
PLXNB2	IL13	
SCN2A	COL11A1	
TBX21	CFI	
ABHD5	EHMT1	
LAMA2	DNMT3A	
ADAMTS9	LOC107303340	
ATP2A2	TTR	
BMP1	IGF2	
C2	AKAP9	
CYP2B9	IL17A	
ITGA8	GATA1	
PBX1	CAV3	
PHGDH	SMAD3	
PSMC6	PIK3R1	
DHX9	CD46	
PAFAH1B1	MRE11	
PSIP1	TYMP	
REN	SETD2	
IDH2	EYA1	
ADAM8	TWNK	
BMP2	ETV6	
HSP90AB1	MAPK1	
NPAS2	TMEM216	
VEGFB	PSTPIP1	
CALM2	POLE	
ENPEP	CSF3R	
FBXO30	FASLG	
HSPA9	CNTNAP2	
HLA-B	PAX6	
CYP2A5	NPHP4	
IHH	ADAR	

PKM	CASR	
PSMD12	ZEB2	
RAPGEF4	DHCR7	
RARG	MIR155	
STAT4	MUSK	
FMR1	CCL2	
IRF9	CASP10	
NPPB	PPARG	
PLEC	MTHFR	
SMC2	ERCC4	
ABLIM1	MT-ND5	
FASL	MTOR	
FOXO1	TH	
HSF1	SURF1	
PGRMC1	ERCC2	
CACYBP	HLA-DQB1	
CASP10	IL1RN	
MDK	TCOF1	
NR2F1	ADA2	
SYN1	CCL5	
COL6A2	NPHP3	
MTHFD1L	EPCAM	
RPS6KA3	APOE	
FGF9	PAX2	
FOXP3	ADA	
PTH	RYR1	
ALDH1A7	POMC	
CD47	DOK7	
COL6A3	IFT172	
COMT	HLA-A	
IFNGR1	MPL	
RBM3	MT-CO1	
ADRB2	EZH2	
CYP3A7	ADAMTS13	
CYP4A10	SBDS	
HSPA1L	TRPS1	
MAOA	MIR15A	
NRCAM	LZTR1	
TRADD	B2M	
CYP4A1	MIR106B	
NCL	CPLANE1	
CKM	MIR10A	
COL11A1	PRKCD	
CYP2C29	SDHC	
HNRNPA2B1	EPO	
PTCH1	MT-CO3	
RAD50	SLC6A4	
SERPINC1	XIAP	
EGR3	SCN1B	
GSTK1	LTBP2	
RND1	HLA-DPB1	
SHH	BSND	
CYP4A14	EDN1	
LCAT	CALR	
NPPA	TMEM231	
RPSA	ABCC2	
PAX6	CDKN1B	
PLTP	KIF7	

CNR1	IL2RA	
COL6A1	MYH7	
FASLG	LRP2	
KLF2	F2	
MAP1B	CHRNA1	
SLC22A7	RB1	
ALDH1A2	GPT	
F10	STAT5B	
UGDH	HBB	
GUSB	GJA1	
PRKDC	PLCG2	
TKT	IL1A	
VCAN	STIM1	
XDH	PSMB8	
IL23A	MMP9	
NES	ARID1B	
S100A10	IDUA	
UGT2B1	SERPINA6	
COL18A1	HPS1	
SDC4	MT-ND4	
UNG	MIR203A	
ANXA4	ADIPOQ	
SC5D	MAOA	
WNT5A	CP	
AKR7A3	ELANE	
CCL3	MPO	
PTGS1	TGFB3	
TH	RELN	
CCN2	KAT6B	
CX3CL1	FIP1L1	
GSTO1	APOL1	
SLC22A2	UGT1A6	
SYP	UGT1A7	
CEBPB	MIR29A	
CYP7B1	FOXG1	
DNAJA1	SMARCB1	
HSPE1	PMP22	
ABCG1	KIAA0586	
ADIPOQ	MT-TK	
APOA4	SCN11A	
LPIN1	TTN	
FMO1	MAP2K2	
GATA3	DGKE	
NCOA1	ATRIP	
G6PC1	CSF3	
SULT2A1	LRP4	
TGFB3	MIR143	
ACE2	CACNB2	
CYP2B2	NOS3	
PDGFA	FLT3	
S100A4	KCNJ5	
SPP1	UGT1A8	
ABCB1B	CHRNG	
BMP4	SOD1	
FGFR2	NPPB	
PPARGC1A	RASA1	
RARB	DMD	
ANXA5	PIGA	

CTSL	TCTN2	
CYP3A2	XK	
GRIN1	DCTN1	
CREB1	ZAP70	
TGFA	RAD51D	
IGF2	IL5	
INSR	POLR1C	
RARA	NPM1	
CCNG1	SCNN1B	
CYCS	PKD2	
PDGFB	SLC25A4	
AFP	MIR145	
S100A8	CSPP1	
S100A9	MIR126	
INS1	DYNC2H1	
RHOA	BCL2	
CYP3A11	ERCC1	
UGT1A6	REN	
CYP2B10	EDNRB	
EPHX2	MPV17	
GPX2	PITX2	
CHEK2	MIR16-1	
CTNNB1	FH	
E2F1	AIP	
GSTM3	MT-ND6	
IL17A	TK2	
GLUL	SCNN1A	
MGMT	SPRED1	
PLAUR	GH-LCR	
SELE	HPS3	
CYP3A5	HPS5	
FAS	HTR2A	
ABCB1A	IQCB1	
AGT	WIPF1	
ME1	TWIST1	
NLRP3	SRSF2	
TLR2	CFHR1	
CYP3A23-3A1	TAP2	
F2	MMP2	
HSPA5	SCO2	
MMP3	SOS2	
TGFB2	ACTB	
CTSB	MIR181A2	
SCARB1	TMEM237	
CDH2	MPZ	
ATM	DNMT3B	
CP	MYH9	
GSTM2	CYBC1	
KDR	HMOX1	
HMGB1	CDK4	
COL1A2	UGT1A9	
HSPD1	TAFAZZIN	
APOA1	HPRT1	
CYP7A1	IL17F	
SULT1A1	NKX2-5	
ABCA1	SLC2A10	
CYP2B1	SMARCA2	
MMP1	MIR146A	

PRL	NLRP12	
GSTA2	APOA1	
PON1	IL18	
APOE	LTA	
HSPB1	KANSL1	
GSTA1	GFPT1	
SOD1	FN1	
IL10	SPINK1	
CYP2C19	MIR122	
POR	C4A	
MDM2	ICAM1	
TNFSF10	ATRIP-TREX1	
CYP2D6	UGT1A	
ABCC3	MDM2	
ALDH1A1	UGT1A4	
EPHX1	STAT4	
UGT1A1	CPT2	
CXCL10	DNAI1	
IL18	KCNJ10	
MKI67	FCGR2A	
CYP2C9	RAB27A	
CYP2B6	KDM6A	
SOD2	FANCC	
TXNRD1	U2AF1	
CXCL2	CHRNB1	
CYP19A1	ATP13A2	
FN1	EDN3	
GSTM1	CD40	
TLR4	CD79A	
TIMP1	SNTA1	
CDK1	SCNN1G	
GADD45A	UGT1A3	
GCLM	EGF	
KCNH2	COL11A2	
NR3C1	H19	
VIM	KCNE2	
EGR1	PREPL	
IGF1	ATR	
